# Quantifying soil moisture impacts on light use efficiency across biomes

**DOI:** 10.1111/nph.15123

**Published:** 2018-03-31

**Authors:** Benjamin D. Stocker, Jakob Zscheischler, Trevor F. Keenan, I. Colin Prentice, Josep Peñuelas, Sonia I. Seneviratne

**Affiliations:** ^1^ Institute for Atmospheric and Climate Science ETH Zurich Zurich 8092 Switzerland; ^2^ CREAF Cerdanyola del Vallès Catalonia 08193 Spain; ^3^ Earth and Environmental Sciences Area Lawrence Berkeley National Lab Berkeley CA 94709 USA; ^4^ Department of Environmental Science, Policy and Management UC Berkeley Berkeley CA 94720 USA; ^5^ AXA Chair of Biosphere and Climate Impacts Department of Life Sciences Imperial College London Silwood Park Campus London SL5 7PY UK; ^6^ CSIC Global Ecology Unit CREAF‐CSIC‐UAB Bellaterra, Catalonia 08193 Spain

**Keywords:** drought impacts, eddy covariance, gross primary productivity (GPP), light use efficiency, photosynthesis, soil moisture, standardized precipitation index, vapour pressure deficit (VPD)

## Abstract

- Terrestrial primary productivity and carbon cycle impacts of droughts are commonly quantified using vapour pressure deficit (VPD) data and remotely sensed greenness, without accounting for soil moisture. However, soil moisture limitation is known to strongly affect plant physiology.
- Here, we investigate light use efficiency, the ratio of gross primary productivity (GPP) to absorbed light. We derive its fractional reduction due to soil moisture (fLUE), separated from VPD and greenness changes, using artificial neural networks trained on eddy covariance data, multiple soil moisture datasets and remotely sensed greenness.
- This reveals substantial impacts of soil moisture alone that reduce GPP by up to 40% at sites located in sub‐humid, semi‐arid or arid regions. For sites in relatively moist climates, we find, paradoxically, a muted fLUE response to drying soil, but reduced fLUE under wet conditions.
- fLUE identifies substantial drought impacts that are not captured when relying solely on VPD and greenness changes and, when seasonally recurring, are missed by traditional, anomaly‐based drought indices. Counter to common assumptions, fLUE reductions are largest in drought‐deciduous vegetation, including grasslands. Our results highlight the necessity to account for soil moisture limitation in terrestrial primary productivity data products, especially for drought‐related assessments.

## Abstract 2

See also the Commentary on this article by https://doi.org/10.1111/nph.15176.

## Introduction

Water availability limits ecosystem productivity across much of the Earth's surface (Beer *et al*., 2010; Schwalm *et al*., 2010; Seneviratne *et al*., 2010; Ahlström *et al*., 2015). In arid, semi‐arid and Mediterranean ecosystems, limiting water availability is a recurrent phenomenon and governs plant growth and phenology (Reichstein *et al*., 2002). In addition, in temperate, boreal and tropical ecosystems, sporadic prolonged dry periods can lead to water‐limited conditions and can have far‐reaching impacts on ecosystem carbon (C) balance (Ciais *et al*., 2005; Granier *et al*., 2007; Doughty *et al*., 2015) and structure (Orth *et al*., 2016). Here, we investigate ‘droughts’, identified by their impact on vegetation productivity. This corresponds most closely to the definition of ‘agricultural droughts’ (Trenberth *et al*., 2007) and also includes seasonally recurring dry conditions.

Most plants tightly co‐regulate water loss and CO_2_ assimilation with the effect that, under conditions of low soil moisture and high atmospheric water vapour pressure deficit (VPD), stomatal conductance and hence assimilation and transpiration rates are reduced in order to prevent exceedingly low leaf water potentials and resulting plant tissue damage from cavitation (Cowan & Farquhar, 1977; McDowell *et al*., 2008; Sperry & Love, 2015). The CO_2_ assimilation rate at the leaf level, or gross primary productivity (GPP) – its integral at the ecosystem level – is the ‘engine’ of C cycling in terrestrial ecosystems. GPP emerges as the dominant driver of year‐to‐year variations in the global land C balance (Poulter *et al*., 2014; Ahlström *et al*., 2015), and is closely controlled by water availability in the rooting zone across much of the Earth's surface (Beer *et al*., 2010; Ahlström *et al*., 2015).

The effects of dryness on CO_2_ assimilation and light use efficiency (LUE, GPP normalized by absorbed light) are represented in global vegetation models and satellite data‐driven products by accounting for VPD only (Running *et al*., 2004; Beer *et al*., 2010; Best *et al*., 2011; Clark *et al*., 2011), soil moisture only (Knorr & Heimann, 2001; Sitch *et al*., 2003; Stocker *et al*., 2013) or both (Medvigy *et al*., 2009; Zaehle & Friend, 2010; Bonan *et al*., 2014). However, model parametrizations are divergent (Medlyn *et al*., 2016; Rogers *et al*., 2017), and there is a lack of empirical data covering diverse ecosystems (Sulman *et al*., 2016). Furthermore, quantifications of impacts by low soil moisture and high VPD have commonly relied on a priori specified functional relationships (Reichstein, 2003; Leuning *et al*., 2005; Pan *et al*., 2006; Verstraeten *et al*., 2006; Granier *et al*., 2007; Yuan *et al*., 2007; Novick *et al*., 2016). These aspects limit the power of global vegetation models and our ability to monitor terrestrial primary productivity from space.

GPP can generally be formulated as the product of the incident photosynthetically active radiation (PAR), the fraction of absorbed PAR (fAPAR) and LUE (Monteith, 1972):

(Eqn 1) GPP=PAR×fAPAR×LUE

fAPAR is commonly derived from remotely sensed greenness indices and captures first‐order effects on GPP by vegetation cover (Wang *et al*., 2014) and – when reflected in ecosystem structural change – its variation during droughts. Data on fAPAR alone have served as the basis for the identification and quantification of C cycle extreme events (Reichstein *et al*., 2013; Zscheischler *et al*., 2013). However, high VPD and dry soil conditions can lead to severely reduced LUE before becoming manifest in vegetation structure (Garbulsky *et al*., 2010). It is commonly held that this affects mostly evergreen ecosystems (Gamon *et al*., 2016; Walther *et al*., 2016), whereas seasonal GPP variations are well captured by remotely sensed greenness in regions dominated by drought‐deciduous vegetation, in particular grasslands (Rossini *et al*., 2012; Verma *et al*., 2014; Ali *et al*., 2016; Konings *et al*., 2017). Hence, the accurate prediction of variations in LUE and its sensitivity to VPD and soil moisture is essential for the simulation of GPP and C cycle variations in response to interannually varying climate.

Satellite data‐based GPP products are widely used in assessments of global C cycle changes, their interannual variability in recent decades and impacts of droughts (Zhao & Running, 2010; Ballantyne *et al*., 2017; Jung *et al*., 2017; Schwalm *et al*., 2017). These commonly rely on the assumption that VPD and vegetation greenness are correlated with soil moisture and other limitations on vegetation productivity, and should thus suffice for model predictions across a wide range of environmental conditions without accounting for direct information on soil moisture (Field *et al*., 1995; Veroustraete *et al*., 2002; Running *et al*., 2004; Heinsch *et al*., 2006; Fisher *et al*., 2008; Biederman *et al*., 2017). The correlation between soil moisture and VPD arises as a result of the feedback between soil moisture, stomatal conductance and transpiration under dry conditions (Seneviratne *et al*., 2010). However, mechanistic considerations suggest that this coupling deteriorates under very dry conditions (Ruddell & Kumar, 2009). Therefore, it has been argued that the combination of dry soil (low soil moisture) and dry air (high VPD) should be considered for the appropriate modelling of plant responses to drought (Egea *et al*., 2011; Sulman *et al*., 2016; Rogers *et al*., 2017). Although needed for the benchmarking of competing representations in models and to improve data‐based estimates of global GPP and C cycle changes, independent observational constraints for soil moisture effects, additional to VPD, are missing.

Recently, global‐scale, satellite‐based observations of soil moisture based on microwave measurements have become available. However, their representativeness is limited to moisture in upper soil layers, complicating their usability for the estimation of productivity of deeper rooting vegetation (Hirschi *et al*., 2014; Dorigo *et al*., 2017). Other information based on surface reflectance (Xiao *et al*., 2004), the Earth's gravitational field and information from the Gravity Recovery and Climate Experiment (GRACE) mission (Tapley *et al*., 2004; Rodell *et al*., 2009; Humphrey *et al*., 2016), or from alternative remotely sensed vegetation indices (PRI (photochemical reflectance index) (Gamon *et al*., 1992, 2016; Peñuelas *et al*., 1995; Goerner *et al*., 2009; He *et al*., 2016), SiF (sun‐induced fluorescence) (Porcar‐Castell *et al*., 2014) or NIR_V_ (near‐infrared reflectance of terrestrial vegetation) (Badgley *et al*., 2017)), has the potential to provide complementary information relevant for the capture of drought impacts on LUE and GPP. However, the spatial resolution of GRACE is very low (*c*. 10^2^ km) and is affected by other surface water storage, and the complementarity of PRI, SiF and NIR_V_ to greenness indices (normalized difference vegetation index (NDVI) and Enhanced Vegetation Index (EVI)) and how their information is to be used to capture drought impacts remain challenging (He *et al*., 2016; Vicca *et al*., 2016).

Eddy covariance measurements provide data on CO_2_ gas exchange at a high temporal resolution (Baldocchi *et al*., 2001). These data can be used to estimate GPP and to reveal, at scales ranging from hours to years, how ecosystem functioning is affected by the combination of multiple, simultaneously changing drivers. The recently published FLUXNET 2015 dataset provides an unprecedented wealth of flux data, complemented by meteorological variables and soil moisture, measured in parallel. However, these data cannot provide direct information on partial effects by soil moisture. Such effects would ideally be quantified in an experimental setup with and without limiting soil water availability around the flux measurement towers (Beier *et al*., 2012). However, this is generally not feasible because of the relatively large spatial extent of tower footprints and the required resources for controlled conditions at this scale. Furthermore, feedbacks between soil moisture and VPD would confound a separation (Beier *et al*., 2012). Hence, analyses of VPD and soil moisture controls commonly rely on a priori specified functional relationships and model‐based analyses of observational data from unmanipulated sites alone (Granier *et al*., 2007; Novick *et al*., 2016).

Here, we identify soil moisture‐related reductions in LUE and derive their empirical functional relationship from data alone, across sites in the FLUXNET 2015 dataset, covering a wide range of biomes and vegetation types. We make use of *c*. 250 000 site days to empirically estimate the potential light use efficiency (LUE_pot_) under hypothetical, non‐soil moisture‐limited conditions. The ratio of actual over potential LUE (fLUE) reveals the timing and quantifies the magnitude of soil moisture effects, separated from VPD effects and additional to changes in vegetation greenness (fAPAR). This analysis thereby provides an impact‐oriented quantification of droughts.

## Materials and Methods

An extended methods description is available as Supporting Information Methods S1 and as reproducible code (rmarkdown) through https://github.com/stineb/nn_fluxnet2015 (doi: https://doi.org/10.5281/zenodo.1158575). fLUE data are available through doi: https://doi.org/10.5281/zenodo.1158524.

### Approach

We quantify the fractional reduction in LUE due to soil moisture, separated from VPD and greenness effects, as the ratio of actual over potential LUE:

(Eqn 2) $$\text{fLUE}={\text{LUE}}_{\mathrm{act}}/{\text{LUE}}_{\mathrm{pot}}$$

**Figure 1:**
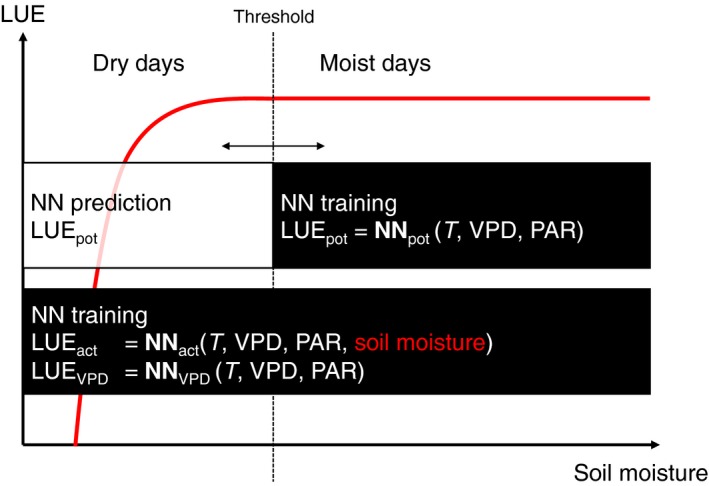
Illustration of the methods for neural network (NN) training. ‘Potential’ light use efficiency (LUE_pot_) is predicted using NN models, trained on the empirical relationship between observed LUE (LUE_obs_) and its predictors, temperature (*T*), vapour pressure deficit (VPD) and photosynthetically active radiation (PAR), during days in which soil moisture is relatively high (‘moist days’). The threshold between moist and dry days is optimized with respect to NN model performance (see the Materials and Methods section). ‘Actual’ LUE (LUE_act_) is derived from NNs using all data and with soil moisture as an additional predictor. LUE_VPD_ is derived from NNs, trained at all data, but without soil moisture as a predictor.

‘Potential’ light use efficiency (LUE_pot_) is predicted using artificial neural networks (NNs, see later), trained on the empirical relationship between observed LUE (LUE_obs_) and its predictors (temperature, VPD and PAR) during days in which soil moisture is relatively high (‘moist days’). All NN training is performed for each site specifically. ‘Actual’ LUE (LUE_act_) is derived from NNs using all data and, in contrast with the NN for LUE_pot_, with soil moisture as an additional predictor (Fig. 1). LUE_obs_ is calculated on the basis of daily total observed GPP_obs_ (GPP_NT_VUT_REF in the FLUXNET 2015 dataset), PAR (based on incoming shortwave radiation, SW_IN_F in FLUXNET 2015) and fAPAR (fraction of absorbed PAR, based on MODIS EVI, extracted for site location) (see Eqn (Eqn 1)). The use of NN‐derived LUE_act_ instead of LUE_obs_ in Eqn (Eqn 2) reduces noise in fLUE as LUE_act_ and LUE_obs_ are affected by similar errors. fLUE distills the effect of accounting for information on soil moisture.

**Figure 2:**
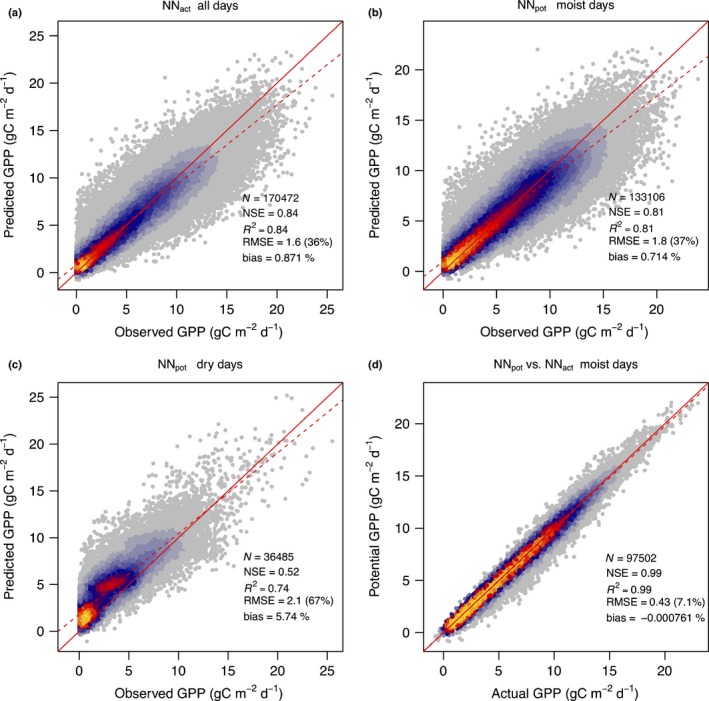
Neural network (NN)‐based predicted vs observed gross primary productivity (GPP). (a) Predicted values are based on the NN model estimating actual light use efficiency, NN_act_, using all input variables (temperature, vapour pressure deficit (VPD), photosynthetically active radiation (PAR), soil moisture) and ‘all days’ data. (b) Predicted values are based on the NN model estimating potential light use efficiency, NN_pot_, trained at data from days above the soil moisture threshold (‘moist days’), using temperature, VPD and PAR as input and evaluated only on ‘moist days’ data. (c) Same as (b) but evaluated on ‘dry days’ data. (d) Predicted values based on NN_pot_ vs predicted values based on NN_act_, evaluated only on ‘moist days’ data. NSE, Nash‐Sutcliffe model efficiency; RMSE, root‐mean‐square error.

**Figure 3:**
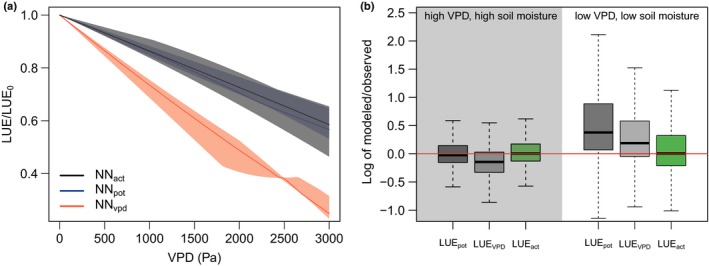
(a) Sensitivity of light use efficiency (LUE) to vapour pressure deficit (VPD), derived from different neural network (NN) setups. The evaluation of sensitivity is performed by holding all predictors, except VPD, at their pre‐drought median (across 20 d preceding drought, identified by fLUE deviation from 1). Shown is the median and the 40–60% quantile range of LUE values, normalized relative to LUE(VPD = 0), pooled across sites for models NN_act_, NN_pot_ and NN_VPD_ (see text and Fig. 1). (b) Distribution of the bias of predicted LUE, based on different NN setups. Biases are evaluated for days with low VPD (below 10% quantile) and low soil moisture (below 0.25 relative soil water content, left) and high VPD (above 90% quantile) and high soil moisture (above 0.75 relative soil water content, right). Boxes represent the interquartile range of values (*Q*_25_, *Q*_75_, logarithm of modelled over observed LUE), whiskers cover *Q*_25_ − 1.5 × (*Q*_75_ − *Q*_25_) to *Q*_75_ + 1.5 × (*Q*_75_ − *Q*_25_). Data are for NN predictions using soil moisture from the SWBM model and represent pooled sites from clusters ‘cDD’ and ‘cGR’ (see ‘Materials and Methods’, ‘Site clustering’)**.**

We limit the NN training to a small number of predictors that are reflective of process understanding regarding the controls on LUE (Prentice *et al*., 2014) and to avoid over‐fitting. Data are split into ‘moist’ and ‘dry’ days, where ‘moist days’ data are used to train NN_pot_ and all data are used to train NN_act_ (Fig. 1). The threshold for splitting is determined by optimal model performance in the face of the trade‐off between the number of data points and including data in which low soil moisture affects fluxes. The criterion applied is the smallest variance in fLUE during moist days of a subset of thresholds in which the difference between LUE_act_ and LUE_pot_ during dry days is highest. The agreement between potential and actual LUE, using the two NN models’ prediction, should be good during ‘moist days’ (high soil moisture). By contrast, LUE_pot_, trained at ‘moist days’ data, is expected to overestimate LUE during days in which soil moisture is low (see Figs 2, 3). With the only difference between NN models being soil moisture as an additional predictor, the ratio fLUE thus indicates the separated effect of soil moisture on LUE. ‘fLUE droughts’ are identified when fLUE falls below a site‐specific threshold. To test the power of VPD as a predictor for LUE, we used an alternative NN setup in which temperature, VPD and PAR are used as predictors (but not soil moisture) and all days (moist and dry) are used for training (LUE_VPD_ = NN_VPD_(*T,* VPD, PAR)). The only difference compared with NN_pot_ is that dry day data are also used for training.

### NN training

Feed‐forward artificial NNs (one hidden layer) are trained (R packages ‘nnet’ (Venables & Ripley, 2002) and ‘caret’ (Kuhn, 2016)) using repeated (five times) five‐fold cross‐validation, where 75% of the data are used for training in each iteration. The learning rate decay rate is set to 0.1 and the number of nodes in the hidden layer is sampled from 4 to 20 (step size 2). The best‐performing NN (by root‐mean‐square error (RMSE)) is selected and the same procedure is repeated five times. In order to reduce scatter in time series, we used the mean across repetitions for further analyses. All predictors are scaled by range to within 0 and 1. NN training is performed for multiple soil moisture datasets (see subsection ‘Soil moisture’) separately. In order to enhance the robustness of NN models under uncertain soil moisture data, we use the mean across the resulting set of fLUE realizations for further analyses.

### Soil moisture

Soil moisture data are based on direct measurements, provided through the FLUXNET 2015 dataset, and five alternative bucket‐type models. Measured soil moisture is provided in units of volume water per volume soil. Where separate data for multiple depths were available, we used them as individual predictors for NN training. As a result of the limited observational soil moisture data availability and mostly unavailable soil moisture data for deep soil layers, we also used simulated soil moisture, provided by alternative, bucket‐type soil water balance models, described later.

#### SPLASH

SPLASH (Davis *et al*., 2017) is based on a Priestley–Taylor formulation for the simulation of evapotranspiration (ET). Two alternative water‐holding capacities (‘bucket depth’) are used: 150 mm (as in Davis *et al*., 2017) and 220 mm (as for the SWBM model, see Orth *et al*., 2013).

#### SWBM

SWBM (Orth *et al*., 2013 *)* uses measured net radiation from local measurements (FLUXNET 2015 data) and generates runoff before the bucket water‐holding capacity (220 mm) is reached (see Eqn 3 in Orth *et al*. (2013), α = 6.4 used here). Similarly, an empirical function down‐scales the fraction of ET to net radiation as a function of soil water content (Eqn 2 in Orth *et al*. (2013), γ = 0.06 used here).

#### ET‐driven bucket

The soil water balance is simulated using precipitation and latent heat flux, measured at the FLUXNET sites. The latent heat flux is converted to mass H_2_O using a constant conversion factor of 2.26476 × 10^6^ J mm^−1^. Latent heat flux data from FLUXNET 2015 (variable LE_F_MDS) are cleaned first if > 80% of the underlying half‐hourly data are gap‐filled, and then gap‐filled using NNs (temperature, PAR, VPD and ET simulated by the SPLASH model as predictors, using R package ‘nnet’ (Venables & Ripley, 2002) and ‘caret’ (Kuhn, 2016), single hidden layer, 20 nodes, 10‐fold cross‐validated). The water‐holding capacity of the ET‐driven buckets is set to 220 mm. Two bucket versions are used. One in which no runoff is generated before the soil water‐holding capacity is reached, and one in which runoff is generated before as in the SWBM model.

All models are driven by observed precipitation, measured at the FLUXNET sites.

### GPP data

Daily data are used from the FLUXNET 2015 Tier 1 dataset, downloaded on 13 November 2016. We use GPP based on the night‐time partitioning method, and the variable u‐star threshold method, named GPP_NT_VUT_REF. In the FLUXNET 2015 dataset, daily values are sums over half‐hourly data. We use only daily values in which < 50% of the respective half‐hourly data are gap‐filled. We further remove data points in which the daytime and night time methods (GPP_DT_VUT_REF and GPP_NT_VUT_REF, resp.) are inconsistent, that is, the upper and lower 2.5% quantiles of the difference between each method's GPP quantification. Finally, we remove all negative daily GPP values.

### Greenness (fAPAR) data

We use MODIS EVI (MOD13Q1, 16 d, 250 m, Collection 5) and tested MODIS fraction of PAR (FPAR) (MOD15A2, 8 d, 1 km) data to quantify fAPAR. As a result of its higher spatial resolution, smaller scatter and smaller tendency to saturate at high values (Fig. S1), EVI is preferred here and all results below are based on analyses with EVI data. Data were downloaded for the pixel surrounding each flux tower location using the ‘MODIStools’ R package. Data were cleaned, eliminating contamination associated with clouds, shadows and snow/ice, and were interpolated to daily values using a Savitzky–Golay smoothing filter (‘signal’ R package) of order 3 and length 31 d. This generally maintains the full seasonal amplitude, crucial for analyses performed here, but does not fully remove noise.

### Site selection

We evaluated fLUE for 135 sites of the total of 166 sites in the FLUXNET Tier 1 dataset, where modelled soil moisture gave consistent results across different models and was consistent with observed soil moisture where available. For 63 sites, the available cleaned data were either too small (< 500 d, 24 sites) or NN results failed performance criteria. Primary criteria, excluding 14 sites, were as follows: mean LUE_act_ during fLUE drought days had to be smaller than LUE_pot_; RMSE of LUE_act_ or LUE_pot_ had to be smaller than 2.8 g C mol^−1^; *R* ^*2*^ of LUE_act_ and LUE_obs_ had to be > 0.5; *R* ^2^ of LUE_pot_ and LUE_obs_ had to be > 0.3. Additional performance criteria, excluding 19 sites, plus six sites excluded by visual inspection, are described in Methods S1. The remaining 71 sites provide 233 369 d of data and were used for subsequent analyses. These are listed in Table 1.

**Table 1:** FLUXNET Tier 1 sites in clusters cDD, cGR, cLS and cNA

Site	Longitude	Latitude	Start	End	IGBP class	Cluster	Reference
AU‐ASM	133.25	−22.28	2010	2013	ENF	cDD	Cleverly (2011)
AU‐DaP	131.32	−14.06	2007	2013	GRA	cDD	Beringer (2013b)
AU‐Fog	131.31	−12.55	2006	2008	WET	cDD	Beringer (2013e)
AU‐Stp	133.35	−17.15	2008	2014	GRA	cDD	NA
SD‐Dem	30.48	13.28	2005	2009	SAV	cDD	Sjöström *et al*. (2008)
SN‐Dhr	−15.43	15.4	2010	2013	SAV	cDD	NA
US‐SRG	−110.83	31.79	2008	2014	GRA	cDD	Biederman *et al*. (2016)
US‐SRM	−110.87	31.82	2004	2014	WSA	cDD	Scott (2016)
US‐Ton	−120.97	38.43	2001	2014	WSA	cDD	Baldocchi (2016a)
US‐Var	−120.95	38.41	2000	2014	GRA	cDD	Baldocchi (2016b)
ZM‐Mon	23.25	−15.44	2000	2009	DBF	cDD	Merbold *et al*. (2011)
AR‐Vir	−56.19	−28.24	2009	2012	ENF	cGR	Posse *et al*. (2016)
AU‐Ade	131.12	−13.08	2007	2009	WSA	cGR	Beringer (2013a)
AU‐DaS	131.39	−14.16	2008	2014	SAV	cGR	Beringer (2013c)
AU‐Dry	132.37	−15.26	2008	2014	SAV	cGR	Beringer (2013d)
AU‐Gin	115.71	−31.38	2011	2014	WSA	cGR	NA
AU‐How	131.15	−12.49	2001	2014	WSA	cGR	Eamus *et al*. (2001)
AU‐Whr	145.03	−36.67	2011	2014	EBF	cGR	Beringer (2013f)
CN‐Qia	115.06	26.74	2003	2005	ENF	cGR	Yu *et al*. (2006)
FR‐LBr	−0.77	44.72	1996	2008	ENF	cGR	Berbigier *et al*. (2001)
FR‐Pue	3.6	43.74	2000	2014	EBF	cGR	Rambal *et al*. (2004)
IT‐Cp2	12.36	41.7	2012	2014	EBF	cGR	Fares & Loreto (2014)
IT‐Cpz	12.38	41.71	1997	2009	EBF	cGR	Garbulsky *et al*. (2008)
IT‐Noe	8.15	40.61	2004	2014	CSH	cGR	Spano *et al*. (2006)
IT‐Ro1	11.93	42.41	2000	2008	DBF	cGR	Rey *et al*. (2002)
IT‐SRo	10.28	43.73	1999	2012	ENF	cGR	Matteucci *et al*. (2015)
AU‐Wom	144.09	−37.42	2010	2012	EBF	cLS	NA
CH‐Oe1	7.73	47.29	2002	2008	GRA	cLS	Ammann *et al*. (2007)
CN‐Cng	123.51	44.59	2007	2010	GRA	cLS	Dong *et al*. (2011)
CZ‐wet	14.77	49.02	2006	2014	WET	cLS	NA
DE‐Akm	13.68	53.87	2009	2014	WET	cLS	NA
DE‐Geb	10.91	51.1	2001	2014	CRO	cLS	Anthoni *et al*. (2004)
DE‐Hai	10.45	51.08	2000	2012	DBF	cLS	Knohl *et al*. (2003)
DK‐Sor	11.64	55.49	1996	2014	DBF	cLS	Pilegaard *et al*. (2001)
FR‐Fon	2.78	48.48	2005	2014	DBF	cLS	Migliavacca *et al*. (2010)
IT‐Col	13.59	41.85	1996	2014	DBF	cLS	Van Dijk & Dolman (2004)
IT‐PT1	9.06	45.2	2002	2004	DBF	cLS	Migliavacca *et al*. (2009b)
IT‐Ren	11.43	46.59	1998	2013	ENF	cLS	Marcolla *et al*. (2005)
IT‐SR2	10.29	43.73	2013	2014	ENF	cLS	Matteucci *et al*. (2015)
IT‐Tor	7.58	45.84	2008	2014	GRA	cLS	Galvagno *et al*. (2013)
NL‐Hor	5.07	52.24	2004	2011	GRA	cLS	Vandermolen *et al*. (2004)
NL‐Loo	5.74	52.17	1996	2013	ENF	cLS	Dolman *et al*. (2002)
RU‐Fyo	32.92	56.46	1998	2014	ENF	cLS	Kurbatova *et al*. (2008)
US‐GLE	−106.24	41.37	2004	2014	ENF	cLS	NA
US‐Me2	−121.56	44.45	2002	2014	ENF	cLS	Sun *et al*. (2004)
US‐MMS	−86.41	39.32	1999	2014	DBF	cLS	Philip (2016)
US‐UMB	−84.71	45.56	2000	2014	DBF	cLS	Gough *et al*. (2008)
US‐UMd	−84.7	45.56	2007	2014	DBF	cLS	Curtis (2016)
US‐WCr	−90.08	45.81	1999	2014	DBF	cLS	Desai (2016a)
BE‐Bra	4.52	51.31	1996	2014	MF	cNA	Carrara *et al*. (2003)
BE‐Vie	6	50.31	1996	2014	MF	cNA	Aubinet *et al*. (2001)
CH‐Fru	8.54	47.12	2005	2014	GRA	cNA	Eugster & Zeeman (2006)
CH‐Lae	8.37	47.48	2004	2014	MF	cNA	Göckede *et al*. (2008)
DE‐Gri	13.51	50.95	2004	2014	GRA	cNA	Gilmanov *et al*. (2007)
DE‐Kli	13.52	50.89	2004	2014	CRO	cNA	Ceschia *et al*. (2010)
DE‐Obe	13.72	50.78	2008	2014	ENF	cNA	Zimmermann *et al*. (2006)
DE‐RuR	6.3	50.62	2011	2014	GRA	cNA	Borchard *et al*. (2015)
DE‐Spw	14.03	51.89	2010	2014	WET	cNA	NA
DE‐Tha	13.57	50.96	1996	2014	ENF	cNA	Grünwald & Bernhofer (2007)
DK‐NuF	−51.39	64.13	2008	2014	WET	cNA	Westergaard‐Nielsen *et al*. (2013)
FI‐Hyy	24.3	61.85	1996	2014	ENF	cNA	Vesala *et al*. (2005)
FI‐Sod	26.64	67.36	2001	2014	ENF	cNA	Suni *et al*. (2003)
IT‐Isp	8.63	45.81	2013	2014	DBF	cNA	Ferréa *et al*. (2012)
IT‐Lav	11.28	45.96	2003	2014	ENF	cNA	Cescatti & Zorer (2003)
IT‐MBo	11.05	46.01	2003	2013	GRA	cNA	Migliavacca *et al*. (2009a)
JP‐SMF	137.08	35.26	2002	2006	MF	cNA	Yamazaki *et al*. (2013)
US‐Ha1	−72.17	42.54	1991	2012	DBF	cNA	Urbanski *et al*. (2007)
US‐Los	−89.98	46.08	2000	2014	WET	cNA	Desai (2016b)
US‐Syv	−89.35	46.24	2001	2014	MF	cNA	Desai (2016b,2016c)
US‐Wi4	−91.17	46.74	2002	2005	ENF	cNA	Noormets *et al*. (2008)

Cluster refers to the clustering of sites according to their greenness response and sensitivity to soil moisture (cDD: drought‐deciduous, 11 sites; cGR: evergreen, 15 sites; cLS: low sensitivity to soil moisture, 23 sites; and cNA: not affected by low soil moisture, 21 sites). Longitude and latitude in decimal degrees. Start and End are the first and last years in which data are available for the respective site. IGBP class is the vegetation class (GRA, grasslands; SAV, savannah; WSA, woody savannah; ENF, evergreen needleleaved forest; EBF, evergreen broadleaved forest; DBF, deciduous broadleaved forest; CSH, closed shrubland; WET, wetland; CRO, cropland; MF, mixed forest). NA, not available.

### Site clustering

First, we identified 21 sites that were not affected by low soil moisture during the period in which measurements were available, that is where available values of relative soil moisture (as a fraction of water‐holding capacity) did not fall below 0.25. This cluster is referred to as cNA (not affected) in the figures below. Second, we identified 23 sites that exhibited a particularly small reduction in fLUE at low soil moisture (cLS, low sensitivity). After removing cNA sites from the 71 sites investigated, cLS sites were identified based on the magnitude of the fLUE reduction with soil moisture approaching zero (termed fLUE_0_, see Fig. S2), and contained sites with fLUE_0_ > 0.8. Third, we used the remaining sites (26) to distinguish between clusters of sites with similar responses in greenness and LUE during droughts. We used a *k*‐means algorithm (R package ‘cluster’ (Maechler *et al*., 2016)) with predefined *k* = 2 (two clusters). Each site formed one observation, with data points given by a vector of length six, containing the median relative reduction of greenness and of fLUE, aggregated across drought events within the respective site and averaged across days −20 − (−1), 0–19 and 20–39 after fLUE drought onset (day 0 is the fLUE drought onset). This separates sites into a cluster with no reduction in greenness and an intermediate reduction in fLUE (cGR, evergreen) and a cluster with a clear greenness reduction, accompanied by a strong reduction in fLUE (cDD, drought‐deciduous).

## Results

### NN performance

Across all sites and days (pooled), the NN‐based GPP predictions following Eqn (Eqn 1) and using LUE_act_ achieve an *R* ^2^ of 0.84 against observed GPP, an RMSE of 1.6 g C m^−2^ d^−1^ and a negligible bias (Fig. 2). A similar performance is achieved by NN_pot_ for the subset of data above the soil moisture threshold (‘moist days’). It should be noted that NN_pot_ refers to the ‘model’, whereas LUE_pot_ refers to its prediction of LUE. By contrast, the NN_pot_‐based prediction of GPP is consistently biased high during days below the soil moisture threshold (‘dry days’). This is a direct consequence of the method and performance criteria (LUE_pot_ > LUE_act_). When comparing LUE_act_ directly with LUE_obs_ = GPP_obs_/(fAPAR_EVI_ × PAR), *R* ^2^ is reduced to 0.7 (Fig. S3). During moist days, the two NN‐based predictions, in which LUE_act_ includes soil moisture as a predictor and LUE_pot_ does not, agree closely (*R* ^2^ = 0.99), indicating that NN_pot_ and NN_act_ capture very similar sensitivities to VPD, temperature and PAR during moist days. This is confirmed by the evaluation of sensitivities of different NN setups to VPD (Fig. 3a).

The sensitivity to VPD warrants particular attention. The NN‐based separation between soil moisture and VPD effects is subject to the accuracy of their sensitivities to partly covarying VPD and soil moisture. We tested this using an additional setup, NN_VPD_, trained at all data, but without soil moisture as a predictor, and evaluated its performance under conditions in which soil moisture and VPD are decoupled, that is, under simultaneously high (low) soil moisture and VPD. In general, NN_VPD_ exhibits a higher sensitivity than NN_act_ and NN_pot_ to VPD (Fig. 3a). Under conditions in which soil moisture is high (non‐limiting) and VPD is high (limiting), the strong sensitivity of NN_VPD_ to VPD leads to an underestimation of LUE_VPD_ compared with LUE_obs_ (Fig. 3b). By contrast, LUE_act_ and LUE_pot_ are both unbiased with respect to LUE_obs_ and hence accurately capture effects of VPD alone. Under conditions of low soil moisture (limiting) and low VPD (non‐limiting), the NN_pot_ model strongly overestimates LUE. Also, LUE_VPD_ is biased high compared with LUE_obs_ under dry soil and moist air conditions. This indicates that information contained in VPD is insufficient to fully capture dryness effects – even with a model that features a relatively high sensitivity to VPD. By contrast, NN_act_ yields unbiased LUE estimates also under these conditions, indicating that its sensitivity to soil moisture alone is accurate.

### Time series

**Figure 4:**
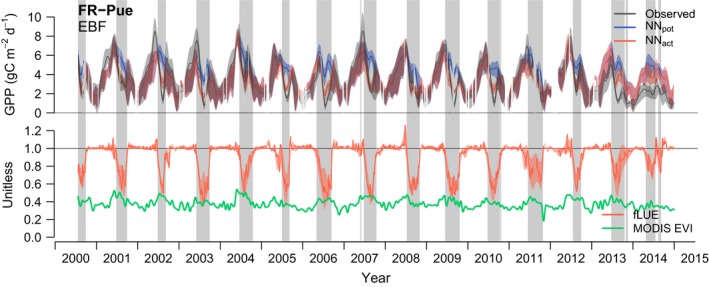
Time series for site ‘FR‐Pue’ (more sites are shown in Supporting Information Fig. S4). Upper panel: time series of observed values and neural network (NN)‐based estimates of gross primary productivity (GPP). Curves are splined daily values with shaded ranges representing splines of minimum and maximum values within 7‐d sliding windows. Lower panel: fractional reduction in light use efficiency due to soil moisture (fLUE) and the fraction of absorbed photosynthetically active radiation (fAPAR) based on MODIS Enhanced Vegetation Index (EVI) data. The shaded range around fLUE represents the splined minimum and maximum fLUE across its quantifications based on different soil moisture datasets, and the solid line is its mean. The site name is given in the upper left corner, together with the vegetation type (EBF, evergreen broadleaved forest). Grey vertical bars illustrate periods identified as ‘droughts’, that is, where fLUE falls below a site‐specific threshold (see the Materials and Methods section).

The evaluation of fLUE reveals substantial soil moisture impacts across a wide range of climatic zones and ecosystem types. Periods of significant fLUE reductions below 1.0 (referred to as ‘fLUE droughts’) are seasonally recurring in dry grasslands (e.g. US‐Var), savannahs (e.g. US‐Ton, AU‐How, AU‐DaS), shrublands (e.g. IT‐Noe), broadleaf evergreen (e.g. FR‐Pue, FR‐LBr) and deciduous (e.g. IT‐Ro1) forests, and occur sporadically in needleleaf evergreen ecosystems in the temperate (e.g. DE‐Tha) and boreal (e.g. FI‐Hyy) zones (Figs 4, S4).

fLUE droughts cover from a few days per year at mesic sites to over 90% of days in desert sites (see also Fig. S5). Impacts of known droughts, for example summer 2003 in Europe, are reflected by particularly strong fLUE reductions, clearly visible at sites with sporadically occurring fLUE droughts (e.g. FR‐LBr and DE‐Tha, Fig. S4). Anomalously high GPP deficits, that is cumulative fLUE deviations from 1, may trigger legacy effects on ecosystem structure via mortality and aboveground primary productivity (Zhang *et al*., 2013; Anderegg *et al*., 2015). We investigated whether high cumulative fLUE deficits are reflected in greenness anomalies, but found no clear relationship between the two (not shown).

### Aligned and aggregated by drought events

**Figure 5:**
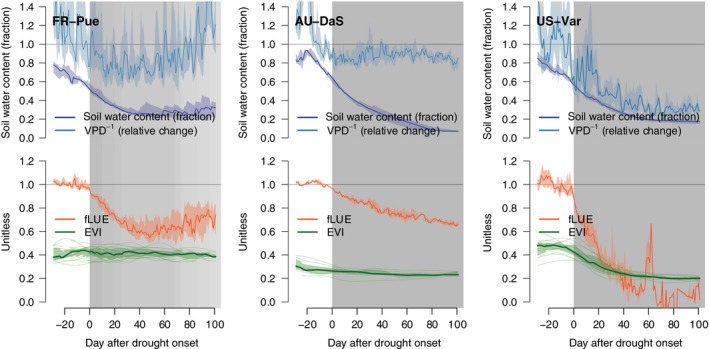
Evolution of soil moisture and vapour pressure deficit (VPD, given in the top panel of each sub‐plot) and fractional reduction in light use efficiency (fLUE, red) and MODIS Enhanced Vegetation Index (EVI) data (second panel, green) throughout drought events. Values shown by VPD ^−1^ (light blue) are calculated by first normalizing values relative to the median of VPD values during 20 d before drought onset and then taking the inverse. Soil moisture values are not normalized, but represent a fraction of water‐holding capacity. Coloured shaded ranges represent the upper and lower quartiles across drought events. The vertical grey shading illustrates the length of individual fLUE drought events. Darker grey shades indicate multiple aligned drought events.

To distill regularities in the co‐evolution of multiple variables during the course of recurring fLUE droughts, all events (grey bands in Fig. 4) per site are aligned by their fLUE drought onset and data are aggregated across drought events. Examples from clusters cGR (FR‐Pue and AU‐DaS) and cDD (US‐Var) are shown in Fig. 5 (further examples in Fig. S7). At all sites, fLUE shows an abrupt transition, whereas soil moisture starts its gradual decline well before the onset of fLUE droughts. This reveals a sharp delineation between a soil moisture‐controlled regime below a given threshold and a regime in which changes in soil moisture do not affect LUE. Although soil moisture follows a very narrow typical course during progressive droughts, VPD exhibits more day‐to‐day variability and does not follow the same pattern of a continued decline during drought events at all sites (Fig. 5). The fLUE reductions during drought periods range from *c*. 10% at mesic sites (DE‐Tha, FI‐Hyy, Fig. S7) to over 90% at the US‐Var grassland site. The course of vegetation greenness during drought periods varies substantially between sites and gives rise to a distinction between ecosystems with similar structural responses to drought (see later).

### Site clustering

**Figure 6:**
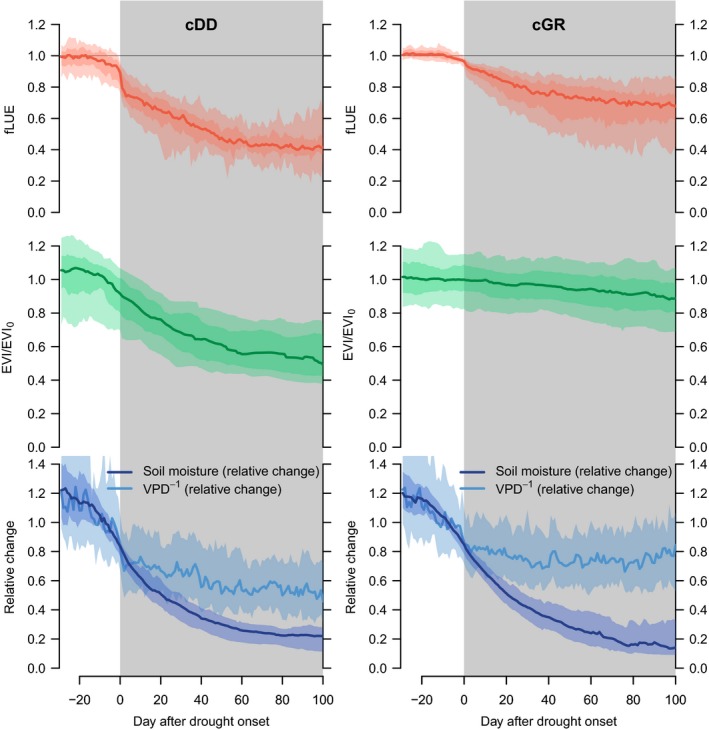
Evolution of variables throughout drought periods, aggregated by clusters cDD (left, drought‐deciduous cluster) and cGR (right, evergreen cluster). Top: fractional reduction in light use efficiency (fLUE). Middle: changes in Enhanced Vegetation Index (EVI), relative to its value before the onset of droughts (median across preceding 20 d). Bottom: changes in soil moisture and vapour pressure deficit (VPD), relative to values before the onset of droughts (median across preceding 20 d). VPD ^−1^ is shown here for a better comparison with soil moisture variations and is calculated as the inverse of the relative VPD change. Data are aligned by the drought onset and aggregated across each event and site in the respective cluster. Bold lines represent the median, and shaded areas represent the upper and lower 10% and 25% quantiles across all events and all sites pooled within a cluster.

After grouping sites not affected by low soil moisture into cluster cNA (‘not affected’, 21 sites) and sites exhibiting a particularly low sensitivity to soil moisture into cluster cLS (‘low sensitivity’, 23 sites, see Materials and Methods), we clustered the remaining 26 sites according to the co‐evolution of fLUE and EVI during droughts. An overview of all sites by clusters is given in Fig. S5. The stark difference in greenness changes is the dominant factor that separates sites into clusters cDD (‘drought‐deciduous’, 11 sites) and cGR (‘evergreen’, 15 sites) (see Fig. 6).

fLUE is reduced most strongly in cDD with values reaching 0.4. This is a 60% reduction in LUE from pre‐drought values due to soil moisture alone. In cluster cGR, fLUE declines to typical values of *c*. 0.7 (30% reduction). In cGR, EVI shows no general response to drought, whereas, in cDD, greenness starts to decline before soil moisture effects on LUE become apparent. Similarly, soil moisture and VPD^−1^ (the inverse of the relative VPD change is shown in Fig. 6) gradually decline well before the onset of fLUE droughts, but start to diverge directly thereafter.

### Functional relationship

**Figure 7:**
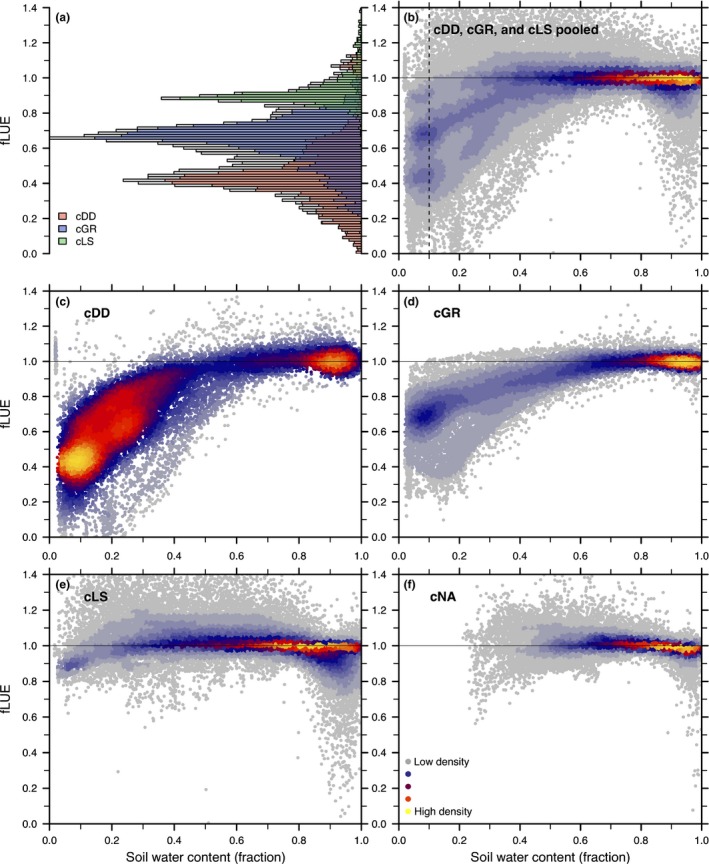
Functional relationship of the fractional reduction in light use efficiency due to soil moisture (fLUE) vs soil moisture by cluster. (a) Distribution of fLUE values at a fractional soil water content below 0.1. Grey bars represent pooled data from drought‐deciduous (cDD), evergreen (cGR) and low sensitivity (cLS) clusters. Coloured bars represent data by clusters. (b) Functional relationship of fLUE vs soil moisture for pooled data from clusters cDD, cGR and cLS. (c–f) Functional relationship by cluster. Colours in the point cloud represent a Kernel Density Estimation (R package ‘lsd’; Schwalb *et al*., 2015) and visualize overlapping points.

The functional relationship between fLUE and soil moisture is analysed by plotting pooled fLUE values against soil moisture (mean across multiple datasets) (Fig. 7). Its general form is similar across clusters, but shows substantial differences in the magnitude of the fLUE reduction with soil moisture approaching zero (fLUE_0_). The distribution of fLUE values at soil moisture below 0.1 from pooled data exhibits three peaks (Fig. 7a). These are associated with distinct fLUE_0_ values within clusters. Reflecting the temporal course shown in Figs 5 and 6, the strongest reduction in fLUE as a function of soil moisture is recorded for cDD, for which most common fLUE_0_ values are *c*. 0.4, but can reach values below 0.1 at sites at which GPP approaches zero, whereas EVI (and FPAR) remain substantially higher (e.g. site US‐Var). The most common magnitude of fLUE_0_ in cGR is *c*. 0.7, but individual sites (e.g. IT‐Noe) show a stronger reduction. By definition, sites in cNA are not affected by very low soil moisture.

Above a relative soil water content of 0.5, effects on LUE are negligible. This is true for all clusters and is consistent with previous studies (Reichstein, 2003; Granier *et al*., 2007). An exception is the reduction in fLUE with soil moisture approaching saturation, as apparent at some sites, mostly in cLS (see also fLUE_1_ column in Fig. S5). This indicates negative effects of very wet soil conditions on GPP. Sites in clusters cDD and cGR show no fLUE reductions at the high end of the soil moisture range. Cluster cLS is, by definition, characterized by small fLUE reductions.

### GPP loss

**Figure 8:**
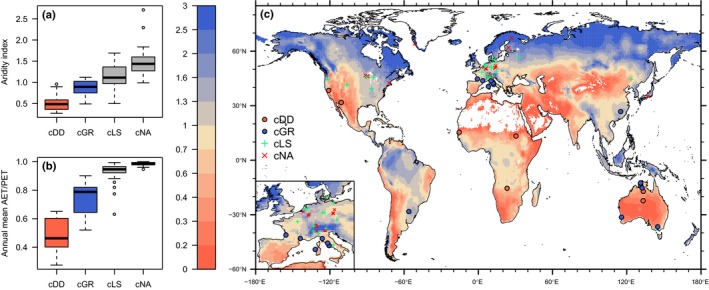
(a) Aridity index by cluster, defined as the ratio of annual precipitation (*P*) over potential evapotranspiration (PET), here calculated for all years in which data were available for the respective sites. Precipitation data are from the FLUXNET 2015 Tier 1 dataset; PET is calculated following the Priestly–Taylor equation, as implemented in the SPLASH model (Davis *et al*., 2017). Clusters are defined based on vegetation responses to drought, with cDD referring to drought‐deciduous, cGR to evergreen, and cLS to vegetation with low sensitivity to low soil moisture. Sites in cNA are not affected by low soil moisture. (b) Annual mean actual evapotranspiration (AET) over PET by cluster. AET and PET are calculated using the SPLASH model (Davis *et al*., 2017). (c) Distribution of sites and clusters across the globe. Colours of land areas represent the aridity index (*P*/PET) with data from (Greve *et al*., 2014). In (a,b), boxes represent the interquartile range of values (*Q*_25_, *Q*_75_,), whiskers cover *Q*_25_ − 1.5 × (*Q*_75_ − *Q*_25_) to *Q*_75_ + 1.5 × (*Q*_75_ − *Q*_25_).

Clusters identified by drought responses exhibit a clear relationship to climate (Fig. 8). Aridity, quantified by precipitation/potential evapotranspiration (*P*/PET) (ratio of annual totals) or the mean of daily actual evapotranspiration (AET)/PET, is systematically related to the association of sites and clusters. This ranges from cDD at the arid end of the spectrum to cGR, cLS and cNA at the moist end.

**Figure 9:**
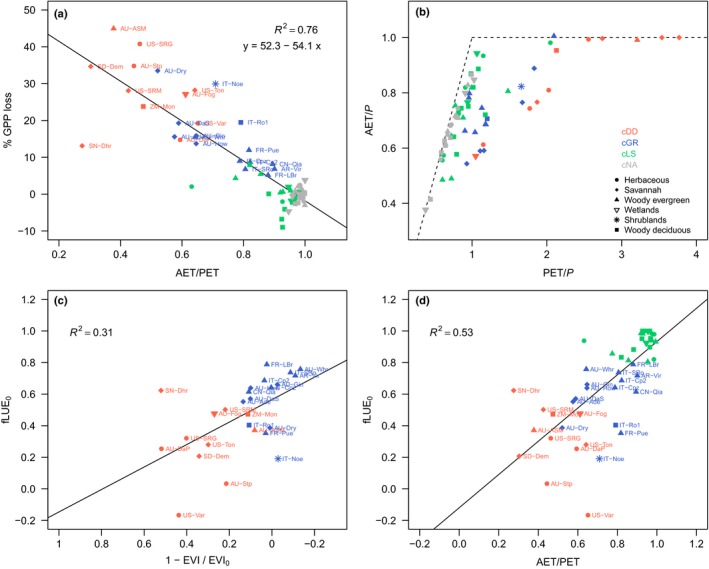
(a) Average annual gross primary productivity (GPP) loss due to soil moisture effects vs the annual average ratio of actual over potential evapotranspiration (AET/PET). (b) Sites in Budyko space (*P* is annual total precipitation). (c) Maximum reduction in the fractional reduction in light use efficiency (fLUE) with soil moisture approaching zero (fLUE_0_) vs greenness change. 1 − EVI/EVI_0_ quantifies the degree of drought‐deciduousness of the vegetation (EVI (Enhanced Vegetation Index) quantifies vegetation greenness, MODIS data), quantified as the relative reduction in greenness during fLUE droughts. Values of 1 − EVI/EVI_0_ = 0 represent no response in greenness during fLUE drought events. The black line represents a linear fit to values from sites in clusters cDD and cGR. (d) fLUE_0_ vs AET/PET. The black line represents a linear fit to values from sites in clusters cDD, cGR and cLS. Clusters are defined based on vegetation responses to drought, with cDD referring to drought‐deciduous, cGR to evergreen, and cLS to vegetation with low sensitivity to low soil moisture. Sites in cNA are not affected by low soil moisture. Symbols represent vegetation types, colours represent clusters (see legend in b).

The magnitude of additional, soil moisture‐related reductions in annual GPP follows the same pattern (Fig. 9). We find that separate effects of soil moisture reduce annual GPP by up to *c*. 40% each year, and that the magnitude of annual GPP reductions scales linearly with the annual mean ratio of AET/PET (Fig. 9a). Annual GPP loss due to low soil moisture is 15–45% at sites in cluster cDD, located in arid and semi‐arid regions (AET/PET < 0.6), and 5–35% at sites in cGR, located in regions of intermediate aridity (0.6 < AET/PET < 0.9, Fig. 9a). At relatively humid sites (AET/PET > 0.9), soil moisture‐limited conditions are sporadic and average annual GPP loss due to soil moisture is relatively small, but highly variable between years (Fig. S4).

### Relationships to water table depth (WTD) and soil properties

To test whether the low fLUE sensitivity to soil moisture in cLS is related to hydrological settings, we extracted WTD values for each site from a global dataset provided at a resolution of 1 km (Fan *et al*., 2013) and an alternative dataset provided at a 0.1° (*c*. 10 km) resolution (de Graaf *et al*., 2015). However, WTD from neither dataset showed any predictive power in explaining the variations in the maximum reduction in fLUE (fLUE_0_, adjusted *R* ^2^ = −0.02 using data by Fan *et al*. (2013) and adjusted *R* ^2^ = −0.01 using data by de Graaf *et al*. (2015)). Similarly, information on soil drainage conditions and available water content, extracted from the Harmonized World Soil Database (Shangguan *et al*., 2014) showed no power in explaining the patterns in the functional relationship between soil moisture and fLUE between clusters.

## Discussion

The quantification of fLUE reveals the threshold, duration and magnitude of soil moisture limitation on GPP and is independent of the use of modelling assumptions or other a priori‐specified functional relationships. Instead, it relies on empirical patterns identified by machine learning and benefits from an unprecedented wealth of data, accessible through the FLUXNET 2015 data release.

NNs, as applied here, cannot account for lagged relationships between predictors and target variables. By targeting LUE, we eliminate effects of ecosystem structural change, which responds more slowly than leaf‐level parameters that determine LUE (stomatal and mesophyll conductance, maximum assimilation rate). Nevertheless, a remaining fraction of variability (see Fig. 2) may not be explainable by the daily environmental forcing data used here, but instead relates to measurement imprecision and biotic responses (Richardson *et al*., 2007). The latter may also be induced by a shift in vegetation composition during the period of flux measurements, if not captured by greenness data (Ahmed *et al*., 2017).

### Relationships to drought indices

**Figure 10:**
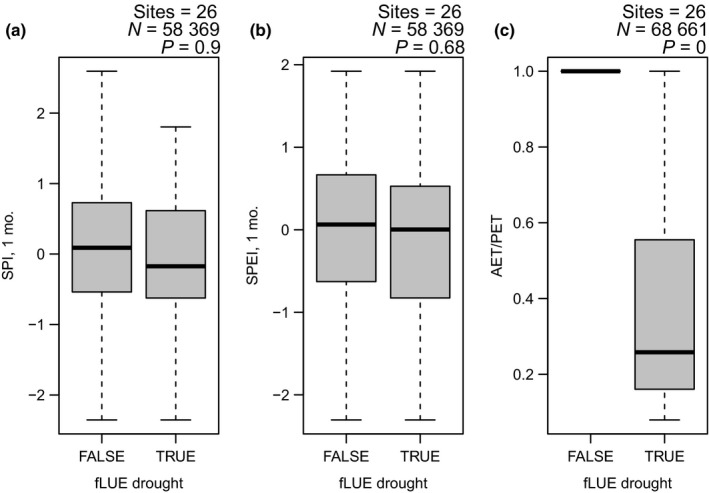
(a) Standardized precipitation–evapotranspiration index (SPEI, 1 month timescale), (b) standardized precipitation index (SPI, 1 month timescale) and (c) the ratio of actual over potential evapotranspiration (AET/PET) during droughts and non‐drought periods, as identified by the fractional reduction in light use efficiency (fLUE). SPI and SPEI were calculated using the R package ‘spei’ based on monthly total precipitation data from WATCH‐WFDEI (0.5°) (Weedon *et al*., 2014) extracted at site locations, covering the years 1990–2013, and (for SPEI) potential evapotranspiration based on Thornthwaite (1948). AET/PET was calculated using precipitation measured at FLUXNET and potential and actual evapotranspiration from the SPLASH model (Davis *et al*., 2017). The box for fLUE = FALSE is collapsed as all AET/PET values in respective days are 1.0. *N* is the total number of daily data points and *P* is the *P* value from an unpaired *t*‐test. Boxes represent the interquartile range of values (*Q*_25_, *Q*_75_) and whiskers cover *Q*_25_ − 1.5 × (*Q*_75_ − *Q*_25_) to *Q*_75_ + 1.5 × (*Q*_75_ − *Q*_25_).

The quantification of fLUE provides an impact‐oriented identification of droughts, related to ‘agricultural droughts’ (Trenberth *et al*., 2007). In climates with seasonally recurring water‐limited conditions, traditional statistical approaches based on anomalies relative to a mean seasonal cycle do not necessarily capture limiting conditions during the dry season (Zscheischler *et al*., 2014). This affects drought assessments based on widely used drought indices (Palmer drought severity index (PDSI), standardized precipitation index (SPI) and standardized precipitation–evapotranspiration index (SPEI)) (Schwalm *et al*., 2017), and other anomaly‐based approaches (Schwalm *et al*., 2010). The approach followed here does not rely on anomaly statistics, but is based on how the relationship between absorbed light and GPP changes, and thereby captures drought effects that operate through physiological mechanisms of water stress. It also identifies regularly recurring water‐stressed conditions (fLUE droughts) which are not captured by SPI or SPEI (Fig. 10). This highlights that climate anomaly‐based drought indices are not directly indicative of plant water stress and thus have limited power for the identification of drought impacts. By contrast, average daily AET/PET is more directly reflective of drought impacts on vegetation productivity.

### Additional GPP loss

We identified soil moisture‐related losses in annual GPP of up to 40% (Fig. 9a). These are additional to effects by dry air (VPD) and drought‐induced loss of photosynthetically active tissue, reflected by reduced greenness. Multiple studies have indicated that remote sensing‐based GPP estimates tend to be biased high under dry conditions (Leuning *et al*., 2005; Turner *et al*., 2005; Pan *et al*., 2006; Verstraeten *et al*., 2006; Mu *et al*., 2007; Maselli *et al*., 2009). The results shown here suggest that soil moisture data will be crucial to resolve this bias. Semi‐arid regions exert a dominant control on the interannual terrestrial C balance and atmospheric CO_2_ growth rate (Poulter *et al*., 2014; Ahlström *et al*., 2015). The substantial and annually recurring soil moisture‐related GPP reductions found at almost all sites in the intermediate to dry part of a global aridity spectrum indicate that the resolution of drought‐related biases in global datasets is important for the accurate monitoring of vegetation activity and C cycle variability. It remains to be shown whether the accurate accounting for drought effects improves the weak performance of remote sensing‐based GPP models in the simulation of interannual variability in semi‐arid regions (Biederman *et al*., 2017).

### Functional relationship for global predictions

The relationship between LUE, VPD and soil moisture, and its generality across different biomes and vegetation types, is needed to inform remote sensing‐based GPP products and as a benchmark for Earth System Models. The evaluation of the data from all pooled sites suggests three distinct groups of sites with values of the maximum fLUE reduction at very low soil moisture (fLUE_0_) clustering at *c*. 0.4, 0.7 and 0.9 (Fig. 7a). The prediction of this functional relationship and fLUE_0_ is key for the accurate modelling of soil moisture effects.

Independent of the clustering of fLUE_0_ values, we found stark differences in phenological responses (greenness change) to drought across sites. This greenness response‐based separation of sites coincides largely with the clusters of fLUE_0_ values in Fig. 7(a). We found that the degree of drought‐deciduousness of the vegetation explains *c*. 30% of the variation in maximum fLUE reductions (fLUE_0_) within clusters cDD and cGR (Fig. 9c). Aridity (annual average AET/PET) also emerges as a good predictor of fLUE_0_ variations and explains *c*. 50% of its variation within clusters cDD, cGR and cLS (Fig. 9d). Greenness changes and aridity may be extracted from global datasets for the prediction of fLUE_0_ and the modelling of soil moisture effects on GPP.

### cDD and fAPAR data

We found that sites with the strongest fLUE reduction are simultaneously characterized by a clear drought phenology (cluster cDD). These sites are located in the driest climates (Fig. 8), are characterized by particularly low ratios of AET/*P* (Fig. 9b) and consist predominantly of grasslands and savannas (Fig. S5). This is consistent with the particularly strong soil moisture control found in arid and semi‐arid regions (Seneviratne *et al*., 2010; He *et al*., 2016; Nicolai‐Shaw *et al*., 2017).

Remotely sensed greenness is often used to estimate vegetation productivity, and relationships are commonly assumed to be strongest in drought‐deciduous vegetation, particularly in grasslands (Gamon *et al*., 1995; Goerner *et al*., 2009; Rossini *et al*., 2012; Verma *et al*., 2014; Ali *et al*., 2016; Konings *et al*., 2017). The parallel reduction in fLUE and EVI recorded at sites in cluster cDD highlights that the information contained in remotely sensed optical greenness indices does not capture the full extent of drought impacts on GPP. Consistent with previous studies that noted limited information in greenness data for the prediction of GPP (Goerner *et al*., 2009; He *et al*., 2016; Biederman *et al*., 2017), we found here that the relative reduction in EVI is smaller than the relative reduction in GPP. In parallel with phenological changes, LUE is strongly reduced, reflecting a correlation between LUE and greenness noted earlier (Sims *et al*., 2006). Hence, the often assumed direct relationship between relative changes in vegetation greenness and productivity in grasslands and other drought‐deciduous ecosystems (Gamon *et al*., 1995; Goerner *et al*., 2009) implies an underestimation of drought impacts on GPP. fLUE shows an even stronger decline during droughts in drought‐deciduous vegetation than in evergreen forest ecosystems.

The analysis presented here is centred around the prediction of LUE, whereby observational LUE, used as a target for NN training, is derived from GPP and remotely sensed fAPAR using Eqn (Eqn 1). This implies a linear relationship between fAPAR data and GPP, and the magnitude of the derived LUE variations during droughts is sensitive to the magnitude of simultaneous relative fAPAR variations. We used MODIS EVI data here to represent fAPAR for its high spatial resolution and relatively low scatter compared with MODIS FPAR, and note that absolute EVI values are generally lower than FPAR, implying higher absolute LUE values. However, our finding of strong LUE reductions in drought‐deciduous ecosystems is robust against the use of different greenness data products. We tested this by alternatively using MODIS FPAR data (not shown). The robustness of the derived relative LUE changes is a result of the linear relationship between EVI and MODIS FPAR across a wide range of values (Fig. S1). However, EVI tends to saturate less than FPAR and NDVI at high values (Huete *et al*., 2010), and should thus imply even smaller relative LUE variations and soil moisture impacts. Whether EVI is affected by significant PAR absorption by non‐photosynthetically active tissue, and thereby underestimates fAPAR reductions in seasonally ‘brown’ vegetation, remains to be addressed.

### cGR and plant strategies

We found that sites with intermediate fLUE reductions are simultaneously characterized by evergreen, mostly woody, vegetation. The respective sites are located at intermediate aridity (Fig. 8). The separation of clusters along the aridity spectrum and in Budyko space (Budyko, 1974; Williams *et al*., 2012) (Fig. 9b) suggests alternative successful plant strategies, governed by water availability and its seasonality. Drought adaptation to maintain intact structure (e.g. deep rooting, adaptation to low leaf water potentials) and protection against LUE reductions and drought damage of green tissue are costly (van der Molen *et al*., 2011), and appear to be a successful plant strategy only at intermediate aridity, accessible mostly to woody vegetation. The dominance of short‐lived, drought‐deciduous vegetation indicates that the prevention of tissue damage under conditions with extensive soil moisture‐limited periods outweighs the costs of rebuilding senesced tissue. In particular, a low ratio of annual average AET/*P* (Fig. 9b) is indicative of a strong seasonality with asynchronicity of precipitation and radiation and limited surface water storage capacity (Milly, 1994; Potter *et al*., 2005; Williams *et al*., 2012), and appears to favour drought‐deciduous vegetation, for example grasslands. This is in contrast with the results of Williams *et al*. (2012), who found high AET/*P* values in grasslands. It should be noted, however, that both AET and PET used here are model‐derived and do not capture additional factors that may influence AET over different vegetation types (different surface–atmosphere coupling or drought sensitivity of stomatal conductance).

### cLS, soil moisture data and WTD

Sites in cLS are generally located in intermediate to wet climates (Fig. 8), maintain a relatively high LUE during dry conditions and we derived only slight reductions related to soil moisture. At the same time, fLUE appears to be negatively affected by very wet conditions (Fig. 7). Together, this may indicate particular local hydrological conditions that enable plants to access water during prolonged periods without precipitation and that inhibit effective soil drainage and promote water‐logged, anaerobic soil conditions after high rainfall. GPP and stomatal conductance have been reported previously to respond negatively within a few days after the onset of water‐logging in laboratory experiments (Terazawa *et al*., 1992; Terazawa & Kikuzawa, 1994). In addition, at the ecosystem scale, persistent water‐logging in a boreal forest has been found to reduce surface conductance, ET and GPP over years (Ohta *et al*., 2014). Reduced LUE under very wet conditions across multiple sites, as found here, suggests that this phenomenon may be common in wet climates.

The soil moisture data used here are representative of the topsoil. Information on WTD across FLUXNET sites is not generally accessible, and whether plants access the saturated zone and are thus capable of withstanding dry conditions in the topsoil without effects on LUE is not generally known, and respective information is mostly lacking in published site descriptions. The possibility of important effects by access to groundwater on ecosystem fluxes and drought responses has been discussed previously (Reichstein *et al*., 2002). We could not find any relationship between fLUE sensitivity and WTD (Fan *et al*., 2013; Shangguan *et al*., 2014; de Graaf *et al*., 2015). In view of the scale and nature of the WTD data used here (1 and 10 km, model‐based), it may be worthwhile to revisit these relationships using actual, site‐specific data.

### Soil moisture vs VPD

VPD and soil moisture are correlated at weekly to monthly timescales (Sulman *et al*., 2016). Also, at daily timescales, a correlation emerges, at least under dry soil conditions (Fig. S6). This underlies the use of only VPD data as surrogate for dryness in global GPP data products, and makes it difficult to unambiguously attribute respective predictive power in a machine‐learning context. We addressed this by assessing whether the sensitivities of different NN setups are accurate and lead to unbiased predictions under conditions in which only one of the two is expected to limit plant productivity. Our analysis confirms that NN_act_ and NN_pot_ pick up the same and appropriate sensitivity to VPD, and that NN_act_ accurately captures the effects of low soil moisture, also when VPD is not limiting.

This separation of effects is enabled by the fact that training data include days in which only one of the two factors limits LUE. This reflects the high day‐to‐day variability in VPD and the known decoupling of VPD and soil moisture at short timescales (Seneviratne *et al*., 2010; Sulman *et al*., 2016). Mechanistically, this arises because VPD is not only affected by soil moisture, but also by the prevailing atmospheric advection and entrainment from the boundary layer (Raupach, 1991; Betts & Ball, 1995). Under wet conditions, soil moisture is not limiting for ET (Seneviratne *et al*., 2010) and thus does not control VPD. A correlation of VPD and soil moisture emerges under intermediately dry conditions. However, VPD and soil moisture become decoupled under very dry conditions and progressive fLUE droughts (Fig. 6). At this stage, soil moisture is depleted, and so its variations have a declining impact on changes in ET and no longer control VPD. Again, atmospheric advection dominates VPD. This is summarized in Fig. S8 and explains why information contained in VPD is not sufficient to fully capture drought effects and to explain the variability in LUE across the full dryness spectrum. These findings are consistent with Ruddell & Kumar (2009).

In conclusion, we show that accounting for soil moisture effects, in addition to VPD, is critical for the estimation of vegetation productivity across the globe and to quantify drought impacts. The general form of the functional relationship between LUE and soil moisture is uniform across contrasting ecosystems and climates, but the magnitude of the maximum LUE reductions is variable and related to shifting plant phenological strategies across the aridity gradient. Evergreen vegetation achieves higher LUE than drought‐deciduous vegetation during dry conditions, but is restricted to zones of intermediate aridity. Newly available global remote sensing‐based soil moisture datasets (Al Bitar *et al*., 2016; Dorigo *et al*., 2017) or alternative vegetation indices (PRI, chlorophyll‐carotenoid index (CCI), SiF) will be useful to provide critical additional information for global GPP estimates. Our results indicate that local hydrological conditions are important for understanding drought impacts on vegetation productivity. The provision of information on WTD should thus be made a high priority for future FLUXNET data distributions.

## Author contributions

B.D.S., J.Z., T.F.K., S.I.S., I.C.P. and J.P. designed the research. B.D.S. performed the research and analysed the data. B.D.S. wrote the manuscript in collaboration with all co‐authors.

## Supporting information

Please note: Wiley Blackwell are not responsible for the content or functionality of any Supporting Information supplied by the authors. Any queries (other than missing material) should be directed to the *New Phytologist* Central Office.

**Fig. S1** MODIS FPAR vs MODIS EVI data.
**Fig. S2** Functional relationship of the fractional reduction in light use efficiency (fLUE) and soil moisture.
**Fig. S3** Neural network‐based predicted vs observed light use efficiency (LUE).
**Fig. S4** Overview of sites by cluster.
**Fig. S5** Coevolution of ecosystem state variables throughout droughts.
**Fig. S6** Time series for different sites.
**Fig. S7** Relationship between vapour pressure deficit (VPD) and soil moisture.
**Fig. S8** Conceptual relationship between vapour pressure deficit (VPD) and soil moisture (SM).

**Methods S1** Extended methods description.
