# Supplementary material for: Quantifying soil moisture impacts on light use efficiency across biomes
Source: New Phytol. 2018 Mar 31;218(4):1430–49. doi: 10.1111/nph.15123 (PMC5969272; doi:10.1111/nph.15123)
Supplement: Supplementary file 1 — Fig. S1 MODIS FPAR vs MODIS EVI data. Fig. S2 Functional relationship of the fractional reduction in light use efficiency (fLUE) and soil moisture. Fig. S3 Neural network‐based predicted vs observed light use efficiency (LUE). Fig. S4 Overview of sites by cluster. Fig. S5 Coevolution of ecosystem state variables throughout droughts. Fig. S6 Time series for different sites. Fig. S7 Relationship between vapour pressure deficit (VPD) and soil moisture. Fig. S8 Conceptual relationship between vapour pressure deficit (VPD) and soil moisture (SM). [file NPH-218-1430-s001.pdf]

New Phytologist

New Phytologist Supporting Information

Article title: Quantifying soil moisture impacts on light use efficiency across biomes

Authors: Benjamin D. Stocker, Jakob Zscheischler, Trevor F. Keenan, I. Colin Prentice, Josep Peñuelas, and Sonia I. Seneviratne

Article acceptance date: 10 February 2018

The following Supporting Information is available for this article:

Fig. S1 *MODIS FPAR versus MODIS EVI data.*

Fig. S2 *Functional relationship of the fractional reduction in light use efficiency (fLUE) and soil moisture.*

Fig. S3 *Neural network-based predicted versus observed light use efficiency (LUE).*

Fig. S4 *Overview of sites by cluster.*

Fig. S5 Coevolution of ecosystem state variables throughout droughts.

Fig. S6 *Time series for different sites.*

Fig. S7 *Relationship between vapour pressure deficit (VPD) and soil moisture.*

Fig. S8 *Conceptual relationship between vapour pressure deficit (VPD) and soil moisture (SM).*

Methods S1 Extended methods description.

Figures

S1 FPAR versus EVI

Fig. S1: *MODIS FPAR versus MODIS EVI data. Colors in the point cloud represent a Kernel Density Estimation (R package "LSD" (Schwalb et al., 2015)) and visualise overlapping points.*

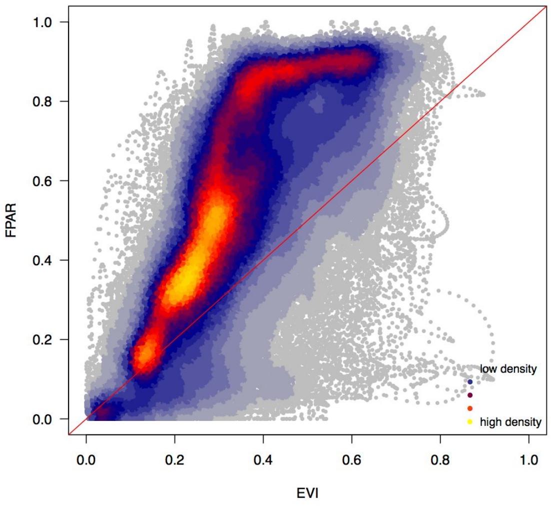

S2 Functional form by site

Fig. S2: *Functional relationship of the fractional reduction in light use efficiency (fLUE) and soil moisture for three typical sites for clusters 1-3. Blue points represent individual days' data. Boxplots represent the distribution of fLUE values within soil moisture bins (20% quantiles), red points are medians within bins, red lines are quadratic fit functions to medians within bins. $fLUE_0$ is defined as the value of the quadratic fit function for soil moisture = 0.*

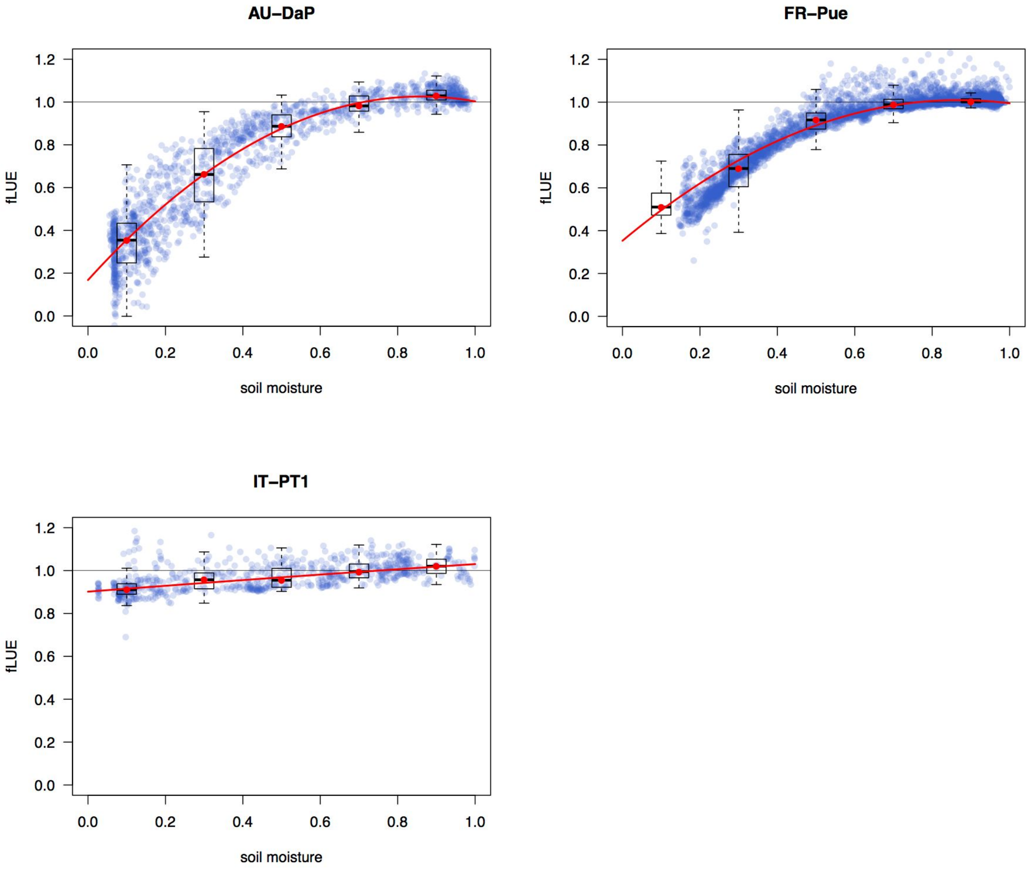

S3 NN performance

Fig. S3: *Neural network-based predicted versus observed light use efficiency (LUE). Observed LUE is calculated as $GPP_{obs}/(fAPAR_{EVI} * PAR_{obs})$, where $fAPAR_{EVI}$ is the fraction of absorbed photosynthetically active radiation, quantified from MODIS EVI data, and PAR_{obs} is the observed photosynthetically active radiation from the FLUXNET 2015 dataset. (a) Predicted values are based on the neural network model estimating actual light use efficiency, NN_{act}, using all input variables (temperature, vapour pressure deficit (VPD), photosynthetically active radiation (PAR), soil moisture) and all days data. (b) Predicted values are based on the neural network model estimating potential light use efficiency, NN_{pot}, trained at data from days above the soil moisture threshold ("moist days"), using temperature, VPD, and PAR as input and evaluated only on moist days' data. (c) same as (b) but evaluated on dry days data . (d) Predicted values based on NN_{pot} versus predicted values based on NN_{act}, evaluated only on moist days data.*

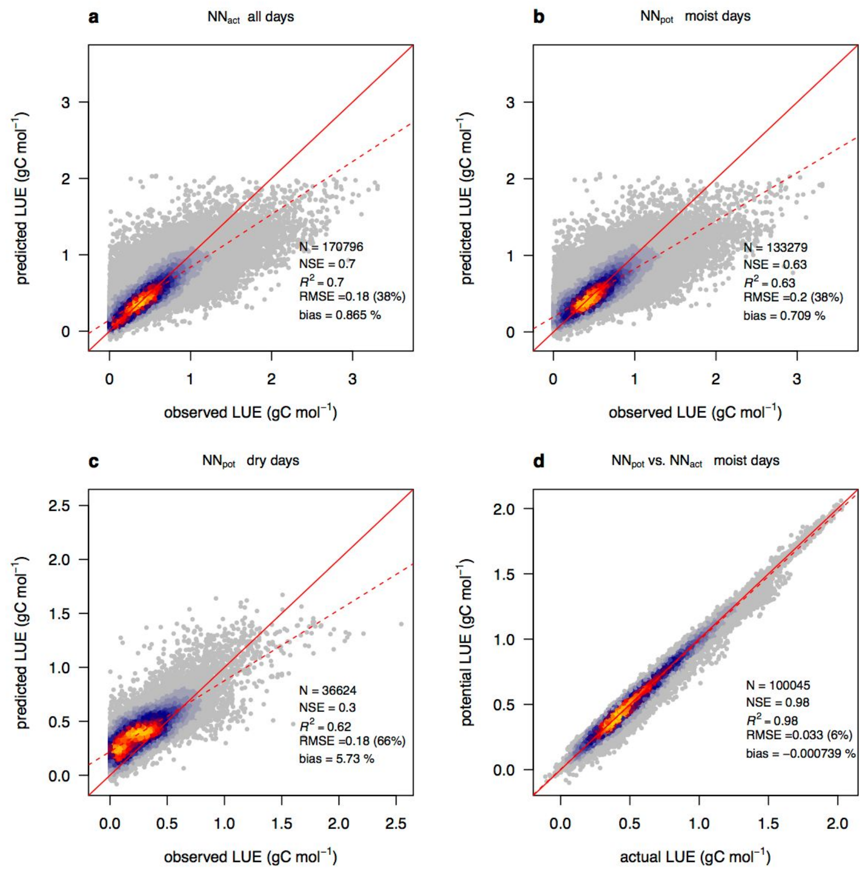

S4 Cluster overview

Fig. S4: *Overview of sites by cluster. ΔGPP (%): percentage mean annual reduction of gross primary productivity due to soil moisture only, calculated based on the fractional reduction in light use efficiency (fLUE), fAPAR and PAR.* ΔGPP_{dr} *(%): same but during drought periods only. 'dr' (%): percentage of days classified as drought based on fLUE.* $fLUE_0$ *and* $fLUE_1$*: median fLUE value within the lower and upper soil moisture quartiles. AI: mean annual aridity index (precipitation over potential evapotranspiration). AET/PET: mean annual ratio of actual over potential evapotranspiration.* WTD_{FMM} *and* WTD_{DG} *(m): water table depth extracted from global datasets by (Fan et al., 2013) and (de Graaf et al., 2015). '*$drain_{HWSD}$*': drainage class from the Harmonized World Soil Databalse, HWSD (1=very poor, 6=somewhat excessive), data by (Shangguan et al., 2014).* AWC_{HWSD}*: available water capacity (mm/m), data by (Shangguan et al., 2014).* veg_{IGBP}*: vegetation class by the International Geosphere-Biosphere Programme (IGBP) classification (GRA=grasslands, SAV=savannah, WSA=woody savannah, ENF=evergreen needleleaved forest, EBF=evergreen broadleaved forest, DBF=deciduous broadleaved forest, CSH=closed shrubland, WET=wetland, CRO=cropland, MF=mixed forest).*

New Phytologist

cDD

	ΔGPP (%)	ΔGPP_{dr} (%)	dr (%)	$fLUE_0$	$fLUE_1$	ΔEVI (%)	AI	AET/PET	WTD_{FMM}	WTD_{DG}	$drain_{HWSD}$	AWC_{HWSD}	veg_{IGBP}
AU–ASM	45	59	75	0.55	NA	8.1	0.35	0.38	17	35	3	50	ENF
AU–DaP	15	35	65	0.42	1	52	0.9	0.59	3.8	11	3	50	GRA
AU–Fog	27	44	65	0.53	0.99	27	0.96	0.61	0	7.7	2	125	WET
AU–Stp	35	52	74	0.27	1.1	21	0.52	0.44	1.2	3.6	2	150	GRA
SD–Dem	35	52	78	0.41	NA	34	0.28	0.3	13	24	6	150	SAV
SN–Dhr	13	26	69	0.7	1.1	52	0.27	0.28	11	5.4	6	100	SAV
US–SRG	41	47	94	0.44	1	40	0.4	0.46	101	123	4	150	GRA
US–SRM	28	46	57	0.63	1.2	22	0.37	0.43	106	80	4	150	WSA
US–Ton	28	44	62	0.47	0.98	30	0.57	0.64	34	2.4	4	100	WSA
US–Var	19	51	45	0.092	1	44	0.6	0.65	25	2.4	4	100	GRA
ZM–Mon	24	48	56	0.56	1	11	0.48	0.47	46	4	6	100	DBF

cGR

	ΔGPP (%)	ΔGPP_{dr} (%)	dr (%)	$fLUE_0$	$fLUE_1$	ΔEVI (%)	AI	AET/PET	WTD_{FMM}	WTD_{DG}	$drain_{HWSD}$	AWC_{HWSD}	veg_{IGBP}
AR–Vir	6.8	13	44	0.76	1	−11	1.1	0.9	6.3	5.6	2	150	ENF
AU–Ade	16	26	71	0.62	1	13	0.88	0.58	12	17	2	150	WSA
AU–DaS	19	27	75	0.65	1	10	0.91	0.59	18	11	3	50	SAV
AU–Dry	33	42	82	0.52	1	0.91	0.63	0.52	2.5	11	4	100	SAV
AU–Gin	16	24	62	0.74	1	−2.6	0.55	0.65	10	4.9	6	100	WSA
AU–How	14	21	72	0.71	1	10	1.1	0.65	7.4	0.41	4	150	WSA
AU–Whr	15	19	76	0.8	1.1	−13	0.49	0.64	27	8.3	4	150	EBF
CN–Qia	8.1	17	54	0.69	1	11	1.1	0.89	27	12	4	150	ENF
FR–LBr	5.2	13	26	0.85	0.98	2.4	1.1	0.88	8.8	5.2	6	100	ENF
FR–Pue	12	29	35	0.51	1	2.9	0.99	0.82	35	9.2	3	15	EBF
IT–Cp2	8.7	18	42	0.75	1	3.5	0.89	0.82	5.3	0.52	5	15	EBF
IT–Cpz	9	19	37	0.74	0.99	0.33	0.88	0.79	5.1	0.52	2	100	EBF
IT–Noe	30	45	57	0.37	1	−2.9	0.62	0.71	23	NA	3	50	CSH
IT–Ro1	19	35	37	0.56	0.99	11	0.87	0.79	12	13	3	50	DBF
IT–SRo	6.8	18	27	0.82	0.99	−8.7	0.99	0.81	5.5	NA	5	15	ENF

New Phytologist

cLS

	ΔGPP (%)	ΔGPP_{dr} (%)	dr (%)	$fLUE_0$	$fLUE_1$	ΔEVI (%)	AI	AET/PET	WTD_{FMM}	WTD_{DG}	$drain_{HWSD}$	AWC_{HWSD}	veg_{IGBP}
AU–Wom	0.61	4.5	0.12	0.98	1	NA	1.2	0.93	65	45	4	150	EBF
CH–Oe1	0.91	9.7	8.2	0.86	1	NA	1.7	0.98	0	30	2	150	GRA
CN–Cng	2	−18	0.94	0.96	1	NA	0.5	0.63	0	2.9	4	150	GRA
CZ–wet	−1.8	12	1.3	1	0.96	NA	0.94	0.96	0	18	2	150	WET
DE–Akm	2	20	2.4	0.97	0.91	NA	1.1	0.95	0.1	1.2	1	150	WET
DE–Geb	−2	20	9.5	1.1	1	NA	0.9	0.93	2.5	10	4	150	CRO
DE–Hai	−1.1	47	3.5	1	0.82	NA	1.2	0.97	46	14	4	150	DBF
DK–Sor	−0.58	13	4.5	0.94	0.97	NA	1.4	0.96	9.3	3.2	5	100	DBF
FR–Fon	−9	51	0.17	1.3	0.88	NA	0.99	0.93	23	4.4	4	50	DBF
IT–Col	0.074	37	2.8	0.96	0.81	NA	1.3	0.88	13	91	3	50	DBF
IT–PT1	8	16	23	0.85	1	NA	0.91	0.82	1.3	4.7	2	150	DBF
IT–Ren	0.24	4.1	5.1	0.94	1	NA	1.3	0.99	56	99	5	50	ENF
IT–SR2	5.4	11	35	0.84	1	NA	1.4	0.86	5.5	NA	5	15	ENF
IT–Tor	0.52	3.7	12	0.99	0.96	NA	1.1	0.97	24	286	3	15	GRA
NL–Hor	−0.23	19	0.89	1	0.98	NA	1.7	0.99	0.37	0.67	1	150	GRA
NL–Loo	0.4	4.9	0.73	0.96	0.98	NA	1.5	0.98	7	1.9	6	100	ENF
RU–Fyo	0.94	13	10	0.87	0.96	NA	1	0.95	25	17	4	150	ENF
US–GLE	0.41	3.7	2.3	1	0.99	NA	1.7	0.92	66	164	4	100	ENF
US–Me2	4.3	11	36	0.92	0.84	NA	0.72	0.77	51	89	5	100	ENF
US–MMS	−2.2	47	1.4	1.1	0.87	NA	1.3	0.96	48	8.3	4	150	DBF
US–UMB	−4.1	44	2	1.1	0.85	NA	0.93	0.93	22	4.8	4	150	DBF
US–UMd	−6.8	57	1.6	1	0.76	NA	1.1	0.92	27	4.8	4	150	DBF
US–WCr	−1.4	22	6.8	1.1	0.89	NA	1.1	0.96	19	30	6	50	DBF

cNA

	ΔGPP (%)	ΔGPP_{dr} (%)	dr (%)	$fLUE_0$	$fLUE_1$	ΔEVI (%)	AI	AET/PET	WTD_{FMM}	WTD_{DG}	$drain_{HWSD}$	AWC_{HWSD}	veg_{IGBP}
BE–Bra	−2.6	NaN	0	NA	0.98	NA	1.3	0.97	6	1.4	2	100	MF
BE–Vie	−2.4	NaN	0	NA	0.98	NA	1.5	0.98	24	48	4	50	MF
CH–Fru	−0.87	11	0.68	NA	0.98	NA	1.8	0.99	17	37	2	150	GRA
CH–Lae	0.41	5.4	4	NA	0.99	NA	1.7	1	40	25	3	15	MF
DE–Gri	0.23	3.2	1.1	NA	1	NA	1.4	0.99	17	30	4	100	GRA
DE–Kli	2.5	9	33	NA	0.98	NA	1.3	0.98	45	30	4	100	CRO
DE–Obe	−0.4	9.9	0.49	NA	0.98	NA	1.6	0.99	79	63	4	50	ENF
DE–RuR	−2	NaN	0	NA	0.98	NA	1.4	1	13	44	4	50	GRA
DE–Spw	−4.7	25	1.1	NA	0.95	NA	0.99	0.95	7.7	2.6	2	150	WET
DE–Tha	2.1	9.9	17	NA	0.97	NA	1.3	0.98	47	30	4	100	ENF
DK–NuF	3.8	9.5	26	NA	1	NA	2.7	1	NA	NA	4	125	WET
FI–Hyy	1.5	11	17	NA	0.99	NA	1.4	0.99	28	11	1	150	ENF
FI–Sod	−3	NaN	0	NA	0.9	NA	1.5	1	9.1	15	1	150	ENF
IT–Isp	−0.86	NaN	0	NA	0.99	NA	2.3	0.98	4.3	21	4	150	DBF
IT–Lav	0.42	7.3	0.52	NA	0.98	NA	1.6	0.99	60	169	3	50	ENF
IT–MBo	0.1	6.7	3.2	NA	0.98	NA	1.3	0.98	15	83	3	50	GRA
JP–SMF	0.15	4.5	2	NA	1	NA	1.7	1	80	1.8	4	150	MF
US–Ha1	−1.1	23	2.3	NA	0.96	NA	1.6	0.99	65	23	6	50	DBF
US–Los	−0.55	17	2.1	NA	0.95	NA	1.1	0.96	0	31	6	50	WET
US–Syv	−0.17	7.9	0.085	NA	0.98	NA	1.1	0.96	9.3	35	6	50	MF
US–Wi4	0.84	4	7.8	NA	1	NA	1.1	0.97	16	16	4	150	ENF

S5 Aligned plots

Fig. S5: Coevolution of ecosystem state variables throughout droughts. Shown are *soil moisture and vapour pressure deficit (VPD, top panel of each sub-plot) and fractional reduction in light use efficiency (fLUE, red) and vegetation greenness, quantified by MODIS EVI (second panel, green). Values shown by VPD^{-1} (light blue) are calculated as the inverse of normalised values relative to the median of VPD values during 20 days before drought onset. Colored shaded ranges represent the upper and lower quartiles across drought events. The vertical grey shading illustrates the length of individual fLUE drought events.*

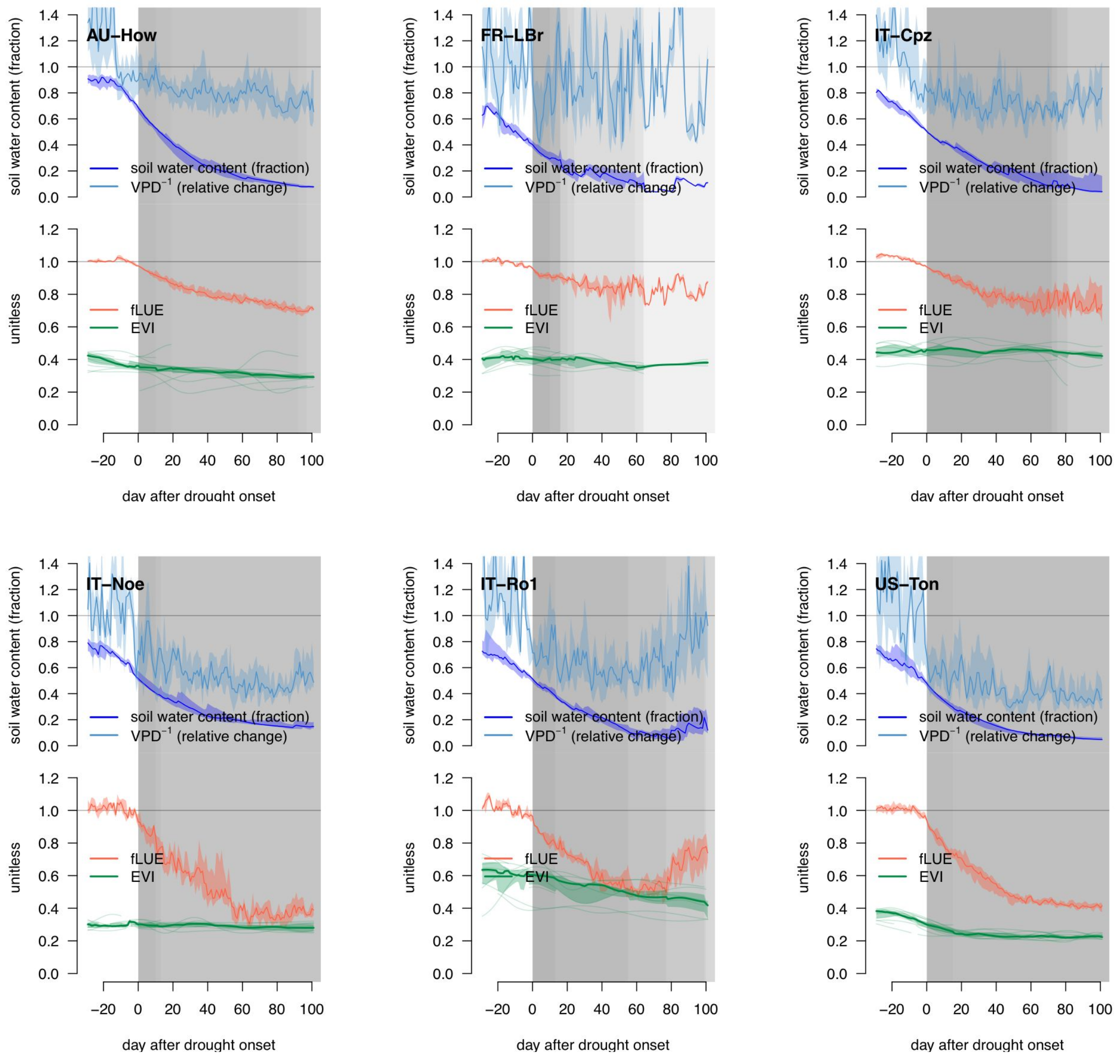

New Phytologist

S6 Time series

Fig. S6: *Time series for different sites. The site name is given in the upper left corner, along with vegetation type (GRA=grasslands, SAV=savannah, WSA=woody savannah, ENF=evergreen needle-leaved forest, EBF=evergreen broadleaved forest, DBF=deciduous broadleaved forest, CSH=closed shrubland). Top panel: Time series of observed values and neural network-based estimates of gross primary productivity (GPP). Curves are splined daily values with shaded ranges representing splines of minimum and maximum values within 7-days sliding windows. Bottom panel: fractional reduction in light use efficiency due to soil moisture (fLUE) and the fraction of absorbed photosynthetically active radiation (fAPAR) based on MODIS EVI data. The shaded range around fLUE represents the splined minimum and maximum fLUE across its quantifications based on different soil moisture datasets and the solid line its mean. Grey vertical bars illustrate periods identified as 'droughts', i.e. where fLUE falls below a site-specific threshold (see Methods).*

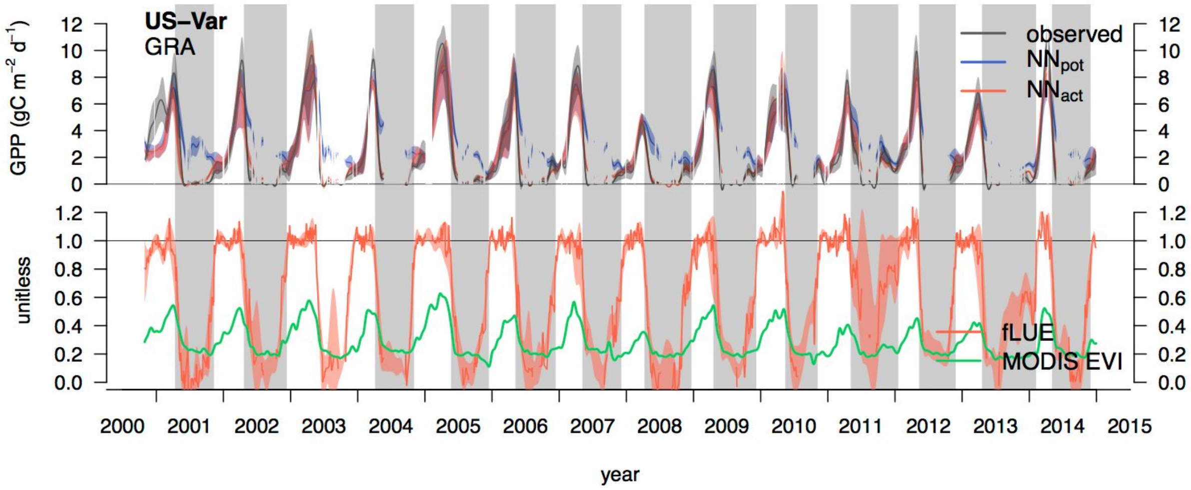

Fig. S6 *(continued*

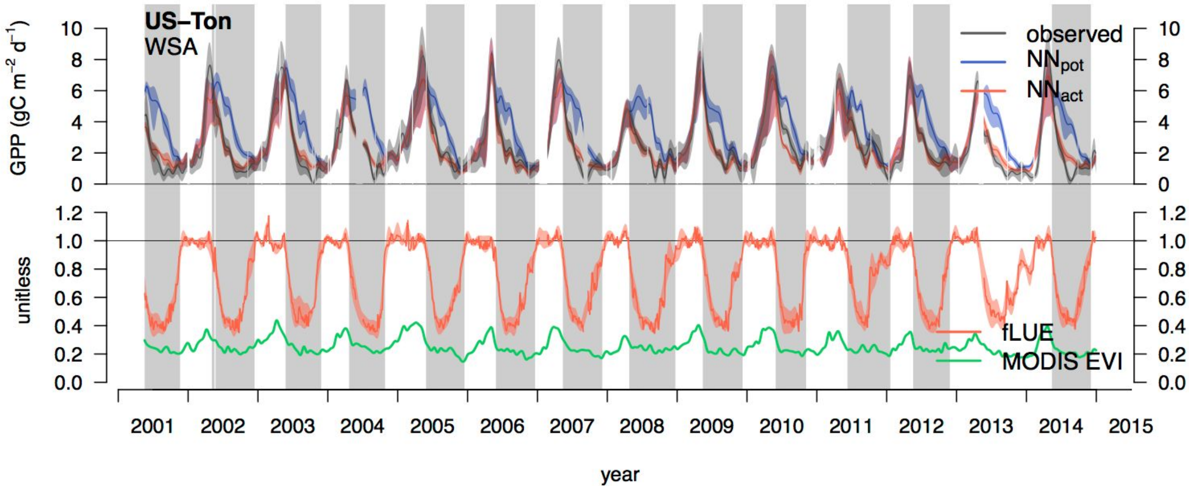

Fig. S6 *(continued)*

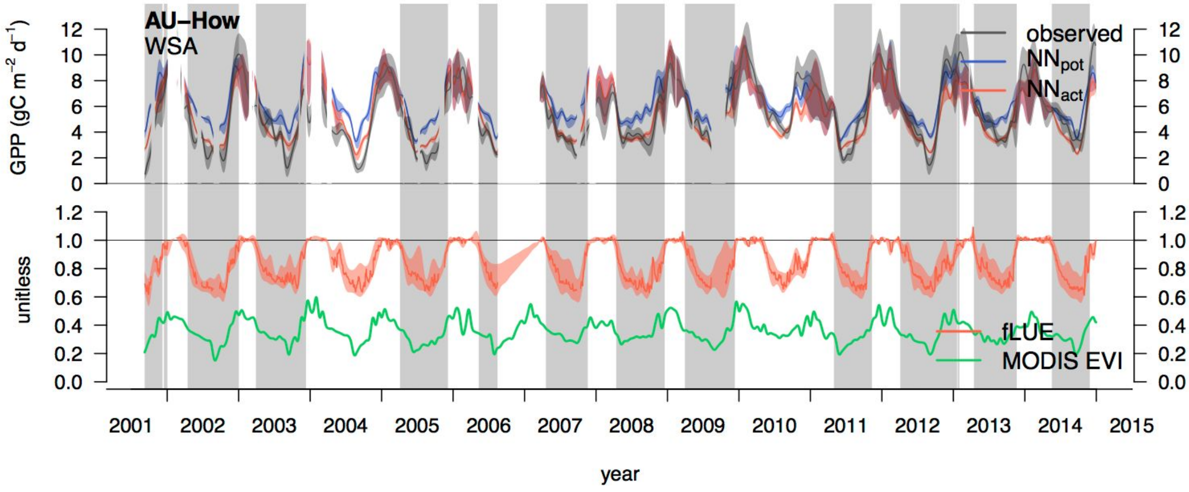

Fig. S6 (continued)

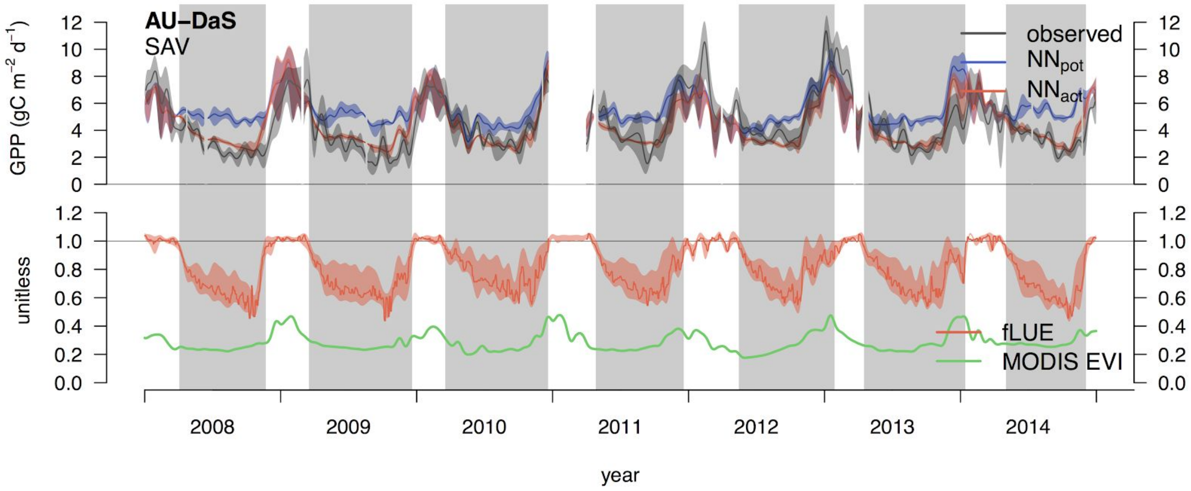

Fig. S6 (continued)

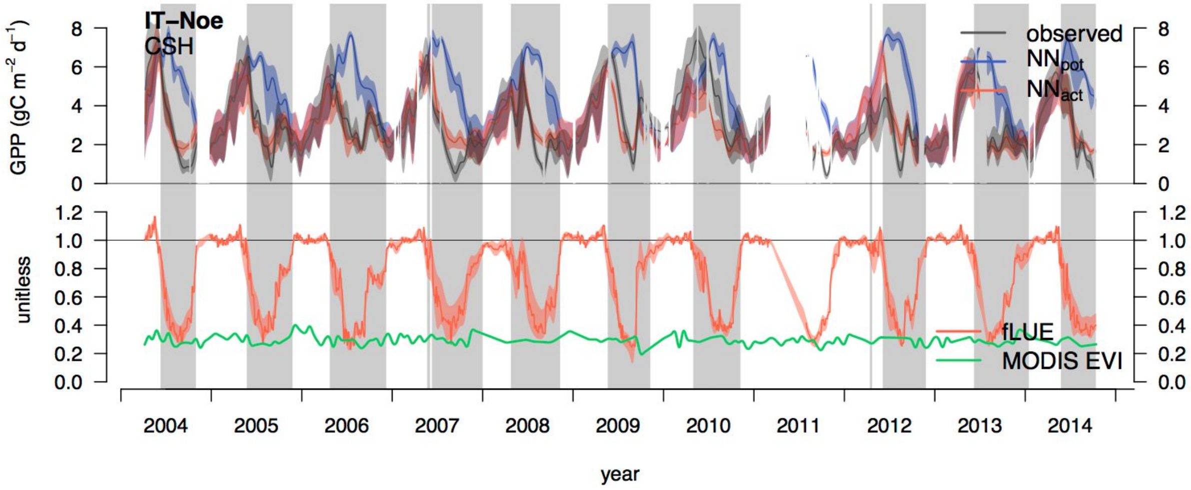

Fig. S6 *(continued)*

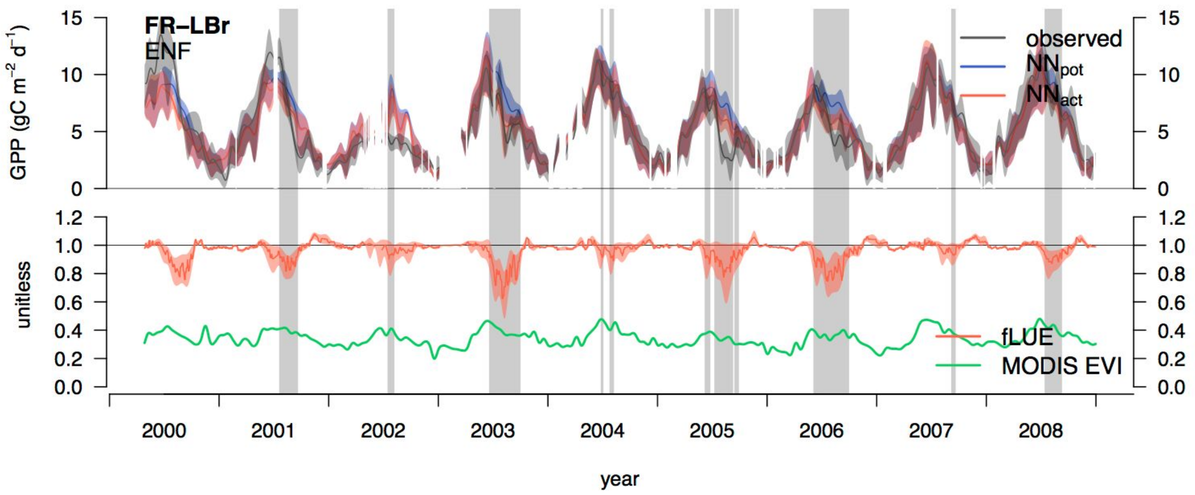

Fig. S6 *(continued)*

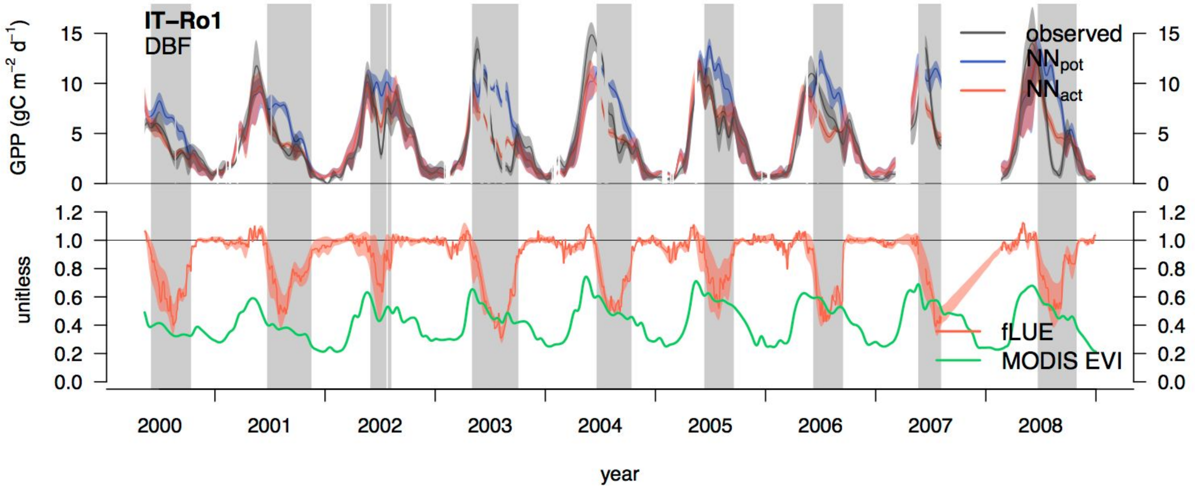

Fig. S6 *(continued)*

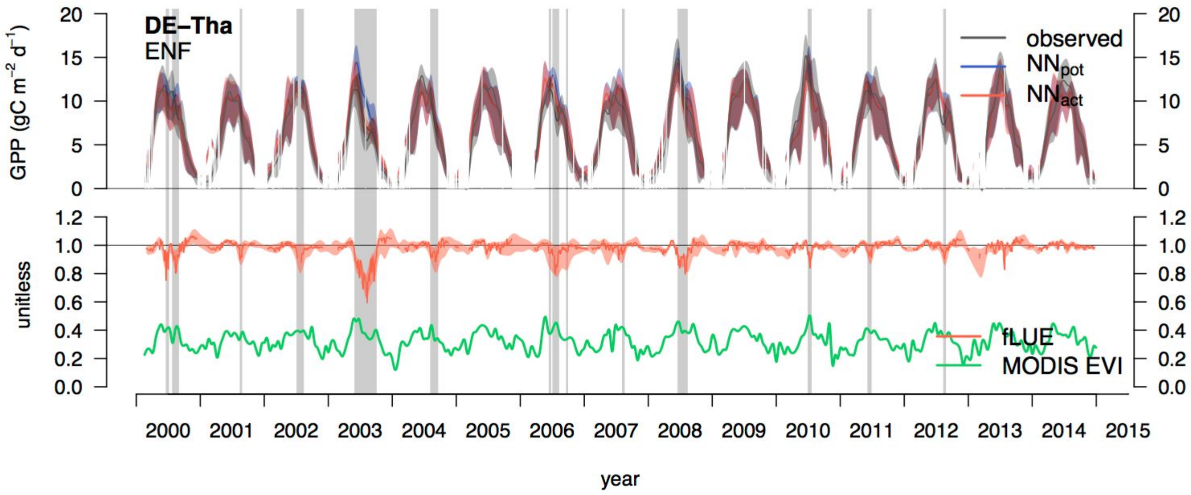

Fig. S6 *(continued)*

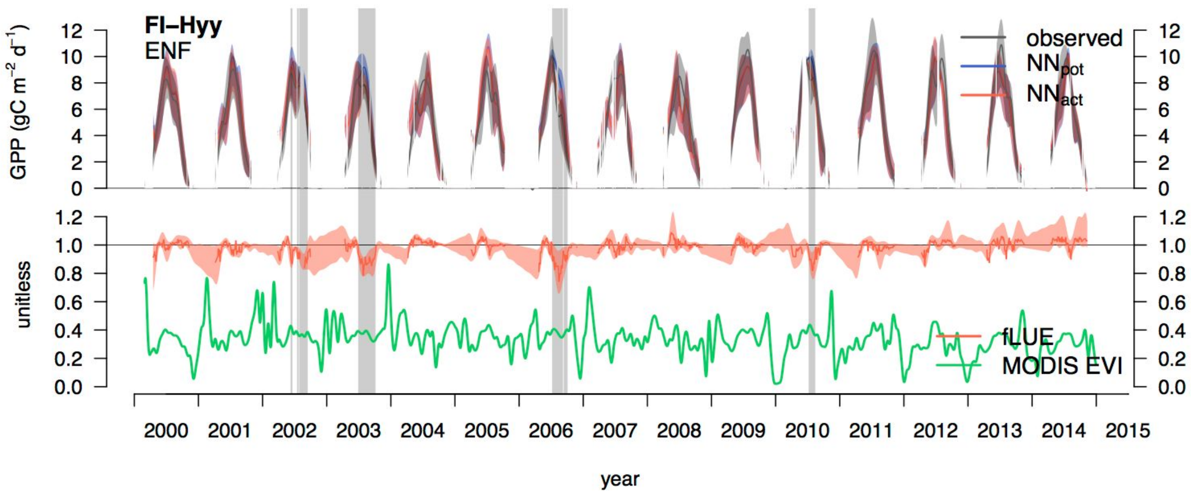

New Phytologist

S7 VPD - soil moisture correlation

Fig. S7: *Relationship between vapour pressure deficit (VPD) and soil moisture. (left) VPD versus soil moisture. (right) VPD during moist and dry days. Boxes represent the interquartile range of values (Q_{25}, Q_{75}), whiskers cover Q_{25}-1.5×(Q_{75}-Q_{25}) to Q_{75}+1.5×(Q_{75}-Q_{25}).*

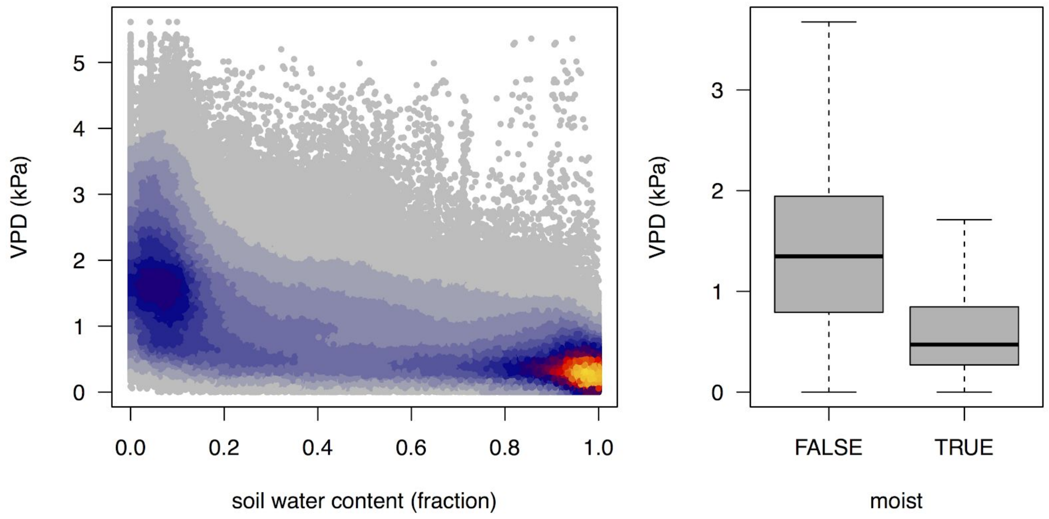

S8 Soil moisture control on VPD

Fig. S8: *Conceptual relationship between vapour pressure deficit (VPD) and soil moisture (SM). ET is evapotranspiration.*

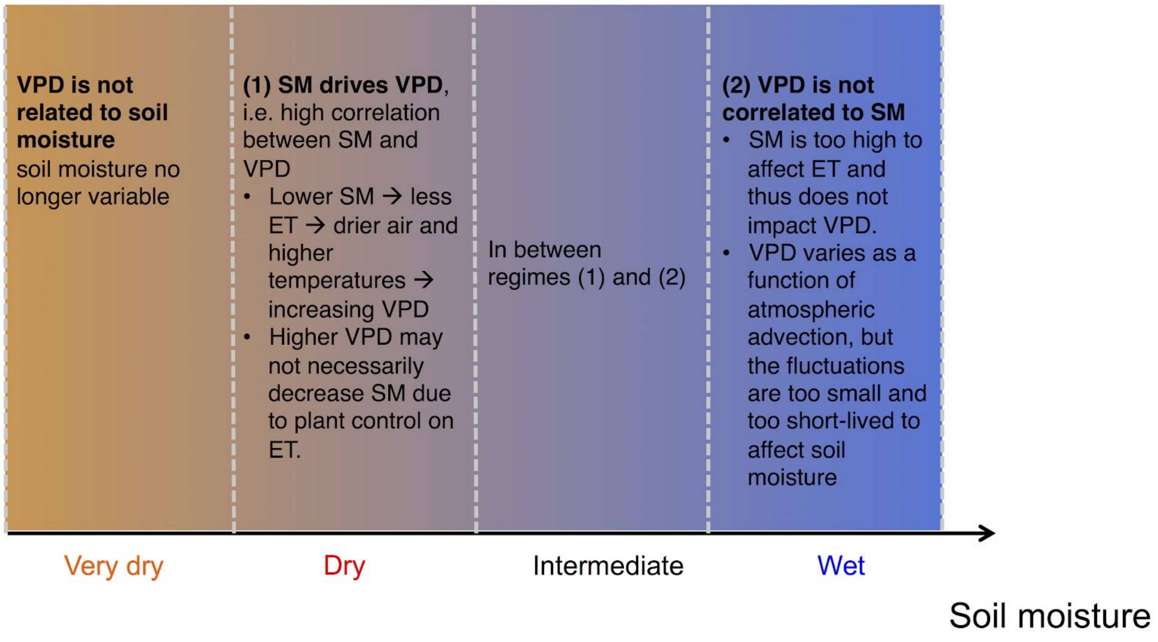

Methods

Methods S1 Extended methods description.

File uploaded separately.

References

Fan Y, Li H, Miguez-Macho G. **2013**. Global patterns of groundwater table depth. *Science* **339**: 940–943.

de Graaf IEM, Sutanudjaja EH, van Beek LPH, Bierkens MFP. **2015**. A high-resolution global-scale groundwater model. *Hydrology and Earth System Sciences* **19**: 823–837.

Schwalb B, Tresch A, Torkler P, Duemcke S, Demel C. **2015**. *LSD: Lots of Superior Depictions*. https://CRAN.R-project.org/package=LSD.

Shangguan W, Dai Y, Duan Q, Liu B, Yuan H. **2014**. A global soil data set for earth system modeling. *Journal of Advances in Modeling Earth Systems* **6**: 249–263.
