# Supplementary material for: Quantifying soil moisture impacts on light use efficiency across biomes
Source: New Phytol. 2018 Mar 31;218(4):1430–49. doi: 10.1111/nph.15123 (PMC5969272; doi:10.1111/nph.15123)
Supplement: Supplementary file 2 — Methods S1 Extended methods description. [file NPH-218-1430-s002.pdf]

Extended Methods to *Quantifying soil moisture impacts on light use efficiency across biomes*

Benjamin D. Stocker, Jakob Zscheischler, Trevor F. Keenan, I. Colin Prentice, Josep Penuelas, and Sonia I. Seneviratne

Contents

Overview . . . 1
IP information . . . 1
Approach . . . 1
Neural network training . . . 2
Process sequence . . . 4
References . . . 29

Overview

Article acceptance date: 10 February 2018

This R-Markdown document provides step-by-step instructions for executing the analysis and producing figures presented in the paper *Quantifying soil moisture impacts on light use efficiency across biomes* by Stocker et al. (2018). The starting point of the present collection of scripts is the data file `./data/modobs_fluxnet2015_s11_s12_s13_with_SWC_v3.Rdata` which contains all the data used for the neural network (NN) training and analysis. Further information about data processing and creating the data file is given below (section 'Data processing'). Using RMarkdown and open access code available through github (https://github.com/stineb/nn_fluxnet2015) this is supposed to allow for full reproducibility of published results - from publicly accessible data files to published figures.

IP information

All code in this repository is free software. It may be redistributed and/or modified under the terms of the GNU General Public License as published by the Free Software Foundation, version 3. Present code is distributed in the hope that it will be useful, but WITHOUT ANY WARRANTY; without even the implied warranty of MERCHANTABILITY or FITNESS FOR A PARTICULAR PURPOSE. See the GNU General Public License (file `./LICENSE`) for more details.

Copyright (C) 2017 Benjamin D. Stocker

Approach

We quantify the fractional reduction in light use efficiency due to soil moisture, separated from VPD and greenness effects as the ratio of actual versus potential LUE:

$$\text{fLUE} = \text{LUE}_{\text{act}} \; / \; \text{LUE}_{\text{pot}}$$

"Potential"" light use efficiency (LUE_{pot}) is predicted using artificial neural networks (NN, see below), trained on the empirical relationship between observed LUE (LUE_{obs}) and its predictors temperature, VPD, and PAR during days where soil moisture is not limiting ("moist days"). All NN training is done for each site specifically. "Actual" LUE (LUE_{act}) is derived from NNs using all data and, in contrast to the NN for LUE_{pot}, with soil moisture as an additional predictor (see Fig. S1).

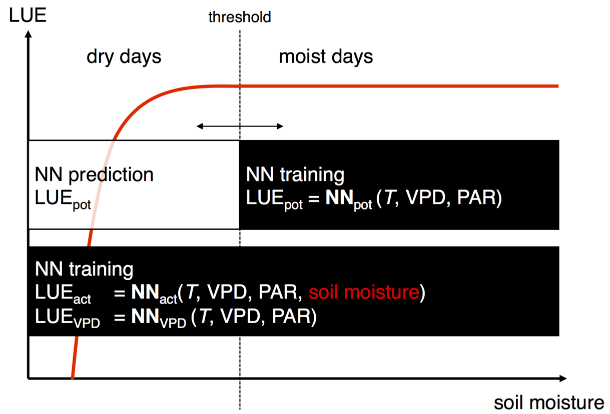

Figure 1: *Illustration of the method for splitting data for NN training into moist and dry days data.*

LUE_{obs} is calculated based on daily total observed GPP (GPP_{obs}), PAR, and fAPAR, measured at each site. We refer to the alternative NN models used for predicting LUE_{act} and LUE_{pot} as NN_{act} and NN_{pot}, respectively.

$$\text{LUE}_{\text{obs}} = \frac{\text{GPP}_{\text{FLX}}}{\text{fAPAR}_{\text{EVI}} \cdot \text{PAR}_{\text{FLX}}} \simeq \begin{cases} \text{NN}_{\text{pot}}(T_{\text{FLX}}, \text{VPD}_{\text{FLX}}, \text{PAR}_{\text{FLX}}), & (T_{\text{FLX}}, \text{VPD}_{\text{FLX}}, \text{PAR}_{\text{FLX}}) \in \text{moist days} \\ \text{NN}_{\text{act}}(T_{\text{FLX}}, \text{VPD}_{\text{FLX}}, \text{PAR}_{\text{FLX}}, \theta), & (T_{\text{FLX}}, \text{VPD}_{\text{FLX}}, \text{PAR}_{\text{FLX}}) \in \text{all days} \end{cases}$$

LUE_{obs} is used as the target variable for NN_{act} and NN_{pot}. Predictor (input) variables are temperature (T), vapour pressure deficit (VPD), and the photon flux density (PAR). Their subscripts refer to the data source with FLX referring to the FLUXNET 2015 dataset and EVI to MODIS EVI. Further information about data sources and processing is given below (section Data processing).

We limit the NN training to a small number of predictors that are reflective of process understanding regarding the controls on LUE (I. C. Prentice et al. 2014) and to avoid over-fitting. The agreement between potential and actual LUE, using the two NN models' prediction, should be good during "moist days" (high soil moisture). In contrast, the potential LUE from the "moist days" NN model is expected to overestimate LUE during days of low soil moisture (see Fig. S2 and S3). With the only difference between NN models being soil moisture as an additional predictor, the ratio fLUE thus indicates the separated effect of soil moisture on LUE. fLUE droughts are identified when fLUE falls below a site-specific threshold (see Process sequence, step 4).

Neural network training

Functions used

Neural networks (1 hidden layer) are trained (R packages `nnet` and `caret`) using repeated (5 times) five-fold cross-validation where 75% of data for training in each iteration. The learning rate decay rate is set to 0.1 and the number nodes in the hidden layer is sampled from 4 to 20 (step 2). The best-performing NN (by RMSE) is selected, the same procedure is repeated five times, and the mean across repetitions is used for further analyses.

The following code snippets are used by function `profile_soilm_neuralnet()` and functions called therein (see below for a full description of the sequence of commands executed to produce results).

1. Data is scaled to within zero and one.

```
preprocessParams <- preProcess( data, method=c("range") )
```

2. Do a 5-fold (argument `number=5`) cross-validation, repeated 5 times (argument `repeats=5`), for the NN training and selecting optimal number of nodes in the NN-model (only one hidden layer in the NN). In each iteration, 75% of the data is used for training and 25% for testing (argument `p=0.75`).

```
traincotrlParams <- trainControl( method="repeatedcv",
                                  number=5, repeats=5, verboseIter=FALSE, p=0.75
                                  )
```

3. Train the NN. The decay of the learning rate parameter is set to 0.1 (This generally yields best results according to additional tests). The number of nodes is sampled from four to twenty in steps of two.

```
tune_grid <- expand.grid( .decay = c(0.1), .size = seq(4,20,2) )
nn <- train(
            lue_obs_evi ~ ppdf + temp + vpd,
            data      = data,
            method    = "nnet",
            linout    = TRUE,
            tuneGrid  = tune_grid,
            preProc   = preprocessParams,
            trControl = traincotrlParams,
            trace     = FALSE
            )
```

This procedure is repeated five times and the mean across individual NN predictions is used for further analysis.

Data splitting into moist and dry days

The threshold for splitting training data into "moist" and "dry" days, where "moist" days' data is used to train NNpot and all data is used to train NNact (see Fig. S1), is determined by optimal performance in the face of the trade-off between the number of data points and including data where low soil moisture affects fluxes. The threshold selection algorithm can be described as follows:

1. Select the five best soil moisture thresholds, for which the difference in the median of the overpredictions (`$ratio`) of the "good days" model during bad days is highest. This is implemented in `profile_soilm_neuralnet.R` as follows:

```
n_best <- 5
diff_good_bad <- data.frame( soilm_threshold = c(), diff = c() )
for (isoilm_trh in soilm_thrsh_avl){
  ## add row to statistics data frame
  addrow <-  data.frame(
                        soilm_threshold = isoilm_trh,
                        diff = median(  filter( df_bad, soilm_threshold==isoilm_trh )$ratio,
                                        na.rm=TRUE
                                      )
                               - median(  filter( df_good, soilm_threshold==isoilm_trh )$ratio,
                                          na.rm=TRUE
                                        )
                        )
  diff_good_bad <- rbind( diff_good_bad, addrow )
}

list_best_soilm_trh <- diff_good_bad$soilm_threshold[
```

```
                          order(-diff_good_bad$diff)][1:min(n_best,nrow(diff_good_bad))
                          ]
```

2. Among the five best thresholds selected by step 1, chose the threshold for which the variance of fLUE during "good days" is minimal.

This is done for each soil moisture dataset separately. This is implemented by function `profile_soilm_neuralnet()` (see Section 'Profiling soil moisture threshold').

Process sequence

The following provides a documentation and instruction to reproduce results presented in the paper. The starting point is the Rdata file `modobs_fluxnet2015_s11_s12_s13_with_SWC_v3.Rdata` where original data downloaded from different sources is stored in a standardised way. Provided is the documentation of NN training, fLUE calculation, drought event detection, NN quality checking, clustering, and plotting (Steps 1-9).

1. Requirements and environment setup

Install required R packages. The execution of the scripts provided here requires the following R packages. Install them if missing (Sorry if some are missing - It's hard to keep the overview):

```
list.of.packages <- c(  "dplyr", "tidyr", "broom", "caret", "nnet",
                        "minpack.lm", "LSD", "zoo", "Metrics", "abind",
                        "caTools", "cgwtools", "hydroGOF", "cluster",
                        "lattice", "ggplot2", "stats", "knitr", "SPEI"
                        )
new.packages <- list.of.packages[!(list.of.packages %in% installed.packages()[,"Package"])]
if( length(new.packages) ) install.packages(new.packages)
```

Create sub-directories

Define working directory. Define the path variable used throughout multiple scripts and functions. `myhome` is used only by functions where argument `testprofile` is `FALSE`, i.e. when evaluating all analyses for all sites (batch execution). Change `myhome` as an absolute path by hand, defining your local parent directory of the present working directory, i.e. where the the repository has been pulled into:

```
workingdir <- getwd()
print( paste("working directory directory:", workingdir ) )
```

```
## [1] "working directory directory: /Users/benjaminstocker/alphadata01/bstocker/nn_fluxnet2015"
```

```
myhome <- "/alphadata01/bstocker/"
```

Get additional utility functions.

```
source("utils_fluxnet2015.R")
```

2. Data processing

The data file `./data/modobs_fluxnet2015_s11_s12_s13_with_SWC_v3.Rdata` contains for each site all the data used for the NN training and all subsequent analyses. The scripts used to process original, downloaded data are not provided here but steps are described below.

Download and load the data file:

```r
load( "./data/modobs_fluxnet2015_s11_s12_s13_with_SWC_v3.Rdata" )
```

The data is organised as a nested list of dataframes. Inside fluxnet$, there is a list for daily (`ddf`), monthly (`mdf`), and annual data (`adf`):

```r
str(fluxnet$`AR-SLu`, max.level = 1)
```

```
## List of 6
##  $ ddf     :List of 7
##  $ ddf_stat:List of 3
##  $ mdf     :List of 4
##  $ mdf_stat:List of 3
##  $ adf     :List of 4
##  $ adf_stat:List of 3
```

Daily data (`ddf`) is used here. This contains the following set of data frames:

```r
str(fluxnet$`AR-SLu`$ddf, max.level = 1)
```

```
## List of 7
##  $ s11         :'data.frame':    1095 obs. of  10 variables:
##  $ s12         :'data.frame':    1095 obs. of  10 variables:
##  $ s13         :'data.frame':    1095 obs. of  10 variables:
##  $ obs         :'data.frame':    1095 obs. of  11 variables:
##  $ inp         :'data.frame':    1095 obs. of  10 variables:
##  $ swc_obs     :'data.frame':    1095 obs. of  5 variables:
##  $ swc_by_etobs:'data.frame':    1095 obs. of  7 variables:
```

- `s11`: contains P-model and SPLASH outputs of simulation s11 (water holding capacity is 220 mm).
- `s12`: contains P-model and SWBM outputs of simulation s12.
- `s13`: contains P-model and SWBM outputs of simulation s13 (water holding capacity is 150 mm).
- `obs`: contains GPP and ET data from FLUXNET 2015 dataset.
- `inp`: climate and greenness (input) data from the FLUXNET 2015 dataset and MODIS EVI and FPAR.
- `swc_obs`: observational soil moisture data from the FLUXNET 2015 dataset.
- `swc_by_etobs`: soil moisture data driven by observational ET.

Soil moisture data

Soil moisture data is based direct measurements, provided through the FLUXNET 2015 dataset, and five alternative bucket-type models. Measured soil moisture is provided in units of volume water per volume soil. We scaled data by range to within 0 and 1. Due to limited observational soil moisture data availability and mostly unavailable soil moisture data for deep soil layers, we also used simulated soil moisture, provided by alternative, bucket-type soil water balance models.

- **SPLASH** (Davis et al. 2017) is based on a Priestley-Taylor formulation for simulating evapotranspiration. Two alternative water holding capacities ("bucket depth") are used, 150 mm (as in (Davis et al. 2017)) and 220 mm (as for the SWBM model, see (Orth, Koster, and Seneviratne 2013)). In the datasets used here, these are referred to as `soilm_splash150`, and `soilm_splash220`.
- **SWBM** (Orth, Koster, and Seneviratne 2013) uses measured net radiation from local measurements (FLUXNET 2015 data) and generates runoff already before the bucket water holding capacity (220 mm) is reached (see Eq. 3 in (Orth, Koster, and Seneviratne 2013), α=6.4 used here). Similarly, an empirical function down-scales the fraction of evapotranspiration to net radiation as a function of soil water content (Eq. 2 in (Orth, Koster, and Seneviratne 2013), γ=0.06 used here). In the datasets used here, this is termed `soilm_swbm`.
- **ET-driven bucket**. The soil water balance is simulated using precipitation and latent heat flux, measured at the FLUXNET sites. The latent heat flux is converted to mass H_2O using a constant conversion factor of 2.26476×10^6 J mm^{-1}. Latent heat flux data from FLUXNET 2015 (variable

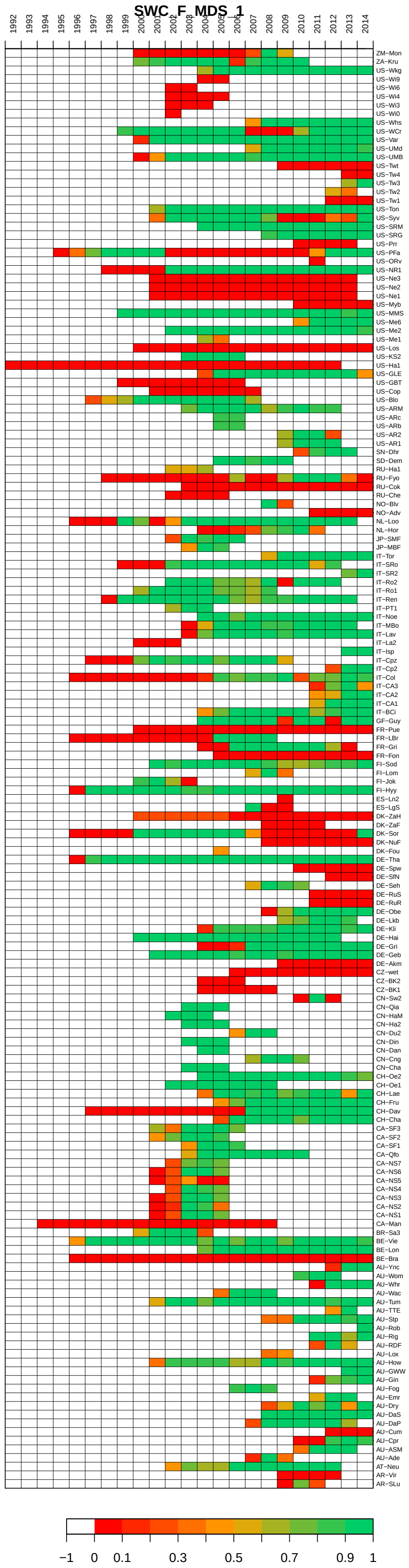

Figure 2: *Soil moisture data availability*

LE_F_MDS) is cleaned first if more than 80% of the underlying half-hourly data is gap-filled, then gap-filled using neural networks (temperature, PAR, VPD, and ET simulated by the SPLASH model as predictors, using R package `nnet` and `caret`, single hidden layer, 20 nodes, 10-fold cross-validated). The water-holding capacity of the ET-driven buckets is set to 220 mm. Two bucket versions are used. One where no runoff is generated before the soil water holding capacity is reached (`soilm_etobs`), and one where runoff is generated before as in the SWBM model (see above, termed `soilm_etobs_ob`).

All models are driven by observed precipitation, measured at the FLUXNET sites.

The following creates a plot showing alternative soil moisture time series (observations and models) for one site ('US-Var').

```
if (!file.exists("fig/soilm")) system("mkdir -p fig/soilm")
source("plot_soilm_fluxnet.R")
plot_soilm_fluxnet( "US-Var", fluxnet[[ "US-Var" ]]$ddf, makepdf=FALSE )
```

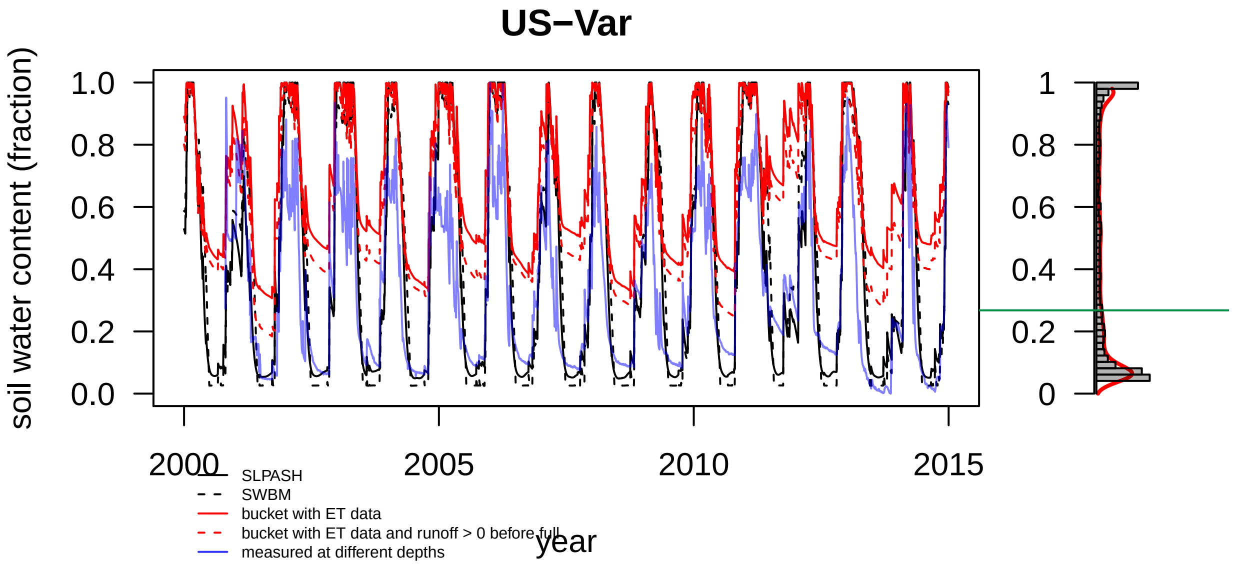

To produce respective plots for all sites (PDFs stored in `./fig/soilm/`), do:

```
system("mkdir -p ./fig/soilm/")
for (sitename in ls(fluxnet)){
  plot_soilm_fluxnet( sitename, fluxnet[[ sitename ]]$ddf, makepdf=TRUE )
}
```

GPP

Daily data are used from the FLUXNET 2015 Tier 1 dataset, downloaded on 13. November, 2016. We use GPP based on the Nighttime Partitioning, and the Variable U-Star Threshold method, named `GPP_NT_VUT_REF`. In the FLUXNET 2015 dataset, daily values are sums over half-hourly data. We use only daily values where less than 50% of respective half-hourly data is gap-filled. We further removed data points where the daytime and nighttime methods (`GPP_DT_VUT_REF` and `GPP_NT_VUT_REF`, resp.) are inconsistent. I.e., the upper and lower 2.5% quantile of the difference between each method's GPP quantification. Finally, we removed all negative daily GPP values.

This is implemented by the following function:

```
clean_fluxnet_gpp <- function( gpp_nt, gpp_dt, qflag_nt, qflag_dt, cutoff=0.80 ){
  ##--------------------------------------------------------------------
  ## Cleans daily data.
  ## gpp_nt: based on nighttime flux decomposition ("NT")
```

```
  ## gpp_dt: based on daytime flux decomposition ("DT")
  ##---------------------------------------------------------------------

  ## Remove data points that are based on too much gap-filled data in the underlying half-hourly data
  gpp_nt[ which(qflag_nt < cutoff) ] <- NA  ## based on fraction of data based on gap-filled half-hourl
  gpp_dt[ which(qflag_dt < cutoff) ] <- NA  ## based on fraction of data based on gap-filled half-hourl

  ## Remove data points where the two flux decompositions are inconsistent,
  ## i.e. where the residual of their regression is above the 97.5% or below the 2.5% quantile.
  res  <- gpp_nt - gpp_dt
  q025 <- quantile( res, probs = 0.025, na.rm=TRUE )
  q975 <- quantile( res, probs = 0.975, na.rm=TRUE )

  gpp_nt[ res > q975 | res < q025  ] <- NA
  gpp_dt[ res > q975 | res < q025  ] <- NA

  ## remove negative GPP
  gpp_nt[ which(gpp_nt<0) ] <- NA
  gpp_dt[ which(gpp_dt<0) ] <- NA

  return( list( gpp_nt=gpp_nt, gpp_dt=gpp_dt ) )
}
```

Greenness data (fAPAR)

We use MODIS EVI (MOD13Q1, 16 days, 250 m) and MODIS FPAR (MOD15A2, 8 days, 1 km) to quantify fAPAR. Due to its higher spatial resolution, smaller scatter and smaller tendency to saturate at high values (see Fig. S1), EVI is generally preferred and all results shown in the paper are based on analyses with EVI data. Results based on FPAR and NDVI data is shown in the SI. Data was downloaded for 81 pixels surrounding the flux tower location (coordinates from FLUXNET 2015) using the `MODISTools` R package. The center pixel's information was used unless quality flags indicated cloudiness or missing data. Data was interpolated to daily values using a Savitzky-Golay smoothing filter (`signal` R package) of order 3 and length 31 days. This generally maintains the full seasonal amplitude but does not fully remove noise. We used the mean seasonal cycle to extend the EVI time series back to years before MODIS EVI data is available (before 2000).

3. Profiling soil moisture threshold

Purpose and outputs

The function `profile_soilm_neuralnet()` samples soil moisture thresholds (0.05-0.60 in steps of 0.05) for separating NN training data into "moist days" and "dry days" data. For each threshold and site, steps described in Section 'Neural network training' are executed. The best-performing threshold is selected as described in Section 'Data splitting into moist and dry days'.

The function also derives various performance metrics and creates diagnostic plots that are saved into the following files and directories (where is the 6-digit FLUXNET site code, e.g. 'FR-Pue'):

- `./data/profile_lue_obs_nn_<sitename>.Rdata`
- `./fig_nn_fluxnet2015/ratio_vs_threshold/`: Illustrates by how much the "good days model" overestimates LUE during bad days, depending on the chosen soil moisture threshold.
- `plot_rsq_vs_smtrh/plot_rsq_vs_smtrh_lue_obs_evi_<soilmoisturedata>_<sitename>.pdf"`: Plots the R^2 of the good days model's prediction of LUE (during "good days").

All NN predictions and performance metrics are saved in `.Rdata` files:

- `profile_lue_obs_evi_nn_<sitename>.Rdata` : Contains NN predictions for each soil moisture threshold and each of the 5 repetitions, as well as respective performance metrics (RMSE and R^2) for each threshold and information about which threshold performs best (see below: Selection of best-performing soil moisture threshold).
- `profile_light_lue_obs_evi_nn_<sitename>.Rdata` : Contains only summary information (performance metrics and information about best-performing threshold) and the the `nnet` object (trained NN model).

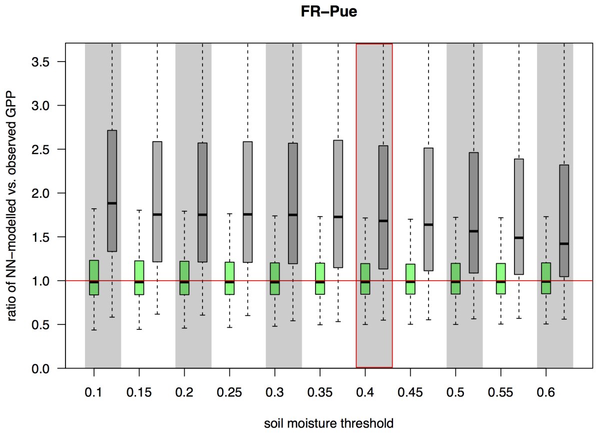

Figure 3: *Ratio of NN-modelled over observed GPP during "dry days", evaluated for each soil moisture threshold. The grey boxes represent predictions based on* NN_{pot}*, the green boxes represent the predictions based on (*NN_{act}*). The red box illustrates the selected soil moisture threshold (0.4 in this case).*

Additional data processing and cleaning

Light use efficiency is calculated and outliers (outside 3 times the interquartile range) are removed:

```
remove_outliers <- function( vec, coef=1.5 ) {
  ## use the command boxplot.stats()$out which use the Tukey's method
  ## to identify the outliers ranged above and below the <coef`>*IQR.
  outlier <- boxplot.stats( vec, coef=coef )$out
  vec[ which( is.element( vec, outlier ) ) ] <- NA
  return( vec )
}
data <- data %>% mutate( lue_obs_evi  = remove_outliers( gpp_obs / ( ppfd * evi  ), coef=3.0 ) )
```

Soil moisture data is normalised to vary between 0 and 1, where 1 is the maximum value in the time series (generally equal to the maximum water holding cacpacity parameter in the respective model, but for the observational data, we don't know the maximum water holding capacity). This is applied to make values from different models and observations comparable on the same scale. Example for soil moisture model output data `soilm_splash150`:

```
data <- data %>% mutate( soilm_splash150 = soilm_splash150 / max( soilm_splash150, na.rm=TRUE ) )
```

Tests have shown that relationships between predictors and observed LUE are noisy during winter in temperate and boreal ecosystems. Therefore we limit data used for NN training to days where average temperature is above 5 degrees Celsius. In `profile_soilm_neuralnet.R`, see

```r
df_nona <- filter( df_nona, temp > 5.0 )
```

The following just removes NA values from the dataframe used for NN training:

```r
df_nona <- cleandata_nn( df_nona, nam_target )
```

Example execution

The code below executes the profiling by soil moisture using MODIS EVI data (argument `nam_target="lue_obs_evi"`). Alternatively, use MODIS FPAR data (`nam_target="lue_obs_fpar"`). If the argument `makepdf=TRUE`, this creates diagnostic plots in subdirectory `./fig_nn_fluxnet2015`, otherwise plots are printed to screen. To speed up things, only one NN is trained only with soil moisture data from one dataset (`varnams_swc=c("soilm_swbm")`), only one repetition for averaging (`nrep=2`), and reduced numbers of soil moisture thresholds sampled (`soilm_threshold=seq( 0.45, 0.55, 0.05 )`). Still, this takes a while - be patient. For the published study, we sampled the a wider range of thresholds (`soilm_threshold = seq( 0.1, 0.60, 0.05 )`) and all available soil moisture datasets (`varnams_swc = c( "soilm_splash150", "soilm_splash220", "soilm_swbm", "soilm_etobs", "soilm_etobs_ob" )`).

```r
source('profile_soilm_neuralnet.R')
profile_soilm_neuralnet(
  "FR-Pue",
  nam_target="lue_obs_evi",
  outdir="./data/profile/",
  soilm_threshold=seq( 0.45, 0.55, 0.05 ), ## reduced range to speed up things
  varnams_swc=c("soilm_swbm"),  ## only SWBM soil moisture to speed up things
  packages="nnet",
  nrep=2,
  makepdf=FALSE,
  use_weights=FALSE,
  overwrite_profile=TRUE
  )
```

The full data from the profiling is now stored in the file `./data/profile/profile_evi_nn_FR-Pue.Rdata`. For example, the best-performing soil moisture threshold (soil water contant as afraction) for dataset `soilm_swbm` is:

```r
load("./data/profile/profile_light_lue_obs_evi_nn_FR-Pue.Rdata")
print( paste( "best-performing soil moisture threshold:", profile_nn_light$`FR-Pue`$soilm_swbm$nnet$bes
```

```
## [1] "best-performing soil moisture threshold: 0.55"
```

Complete batch execution

The profiling function described above is executed for 135 sites out of all 166 in the FLUXNET Tier 1 dataset, where modelled soil moisture gave consistent results across different models and was consistent with observed soil moisture where available (observational soil moisture availability is shown above). This step of site exclusion was done by visual judgment based on the comparison of soil moisture time series for each site (Figures created above, see Section '2. Data processing, Soil moisture' and stored in `./fig/soilm/`). The table below shows the judment results (only first 7 rows are shown. Set `n=166` to see the full table).

Table 1: **Table**: Data usability based on soil moisture, **1** = Observational data available for multiple depths. Use observational data for NN. **2** = Observational data incomplete or inconsistent with ET bucket. ET bucket consistent with SPLASH bucket. Use ET bucket for NN. **3** = Observational data incomplete or inconsistent with ET bucket. ET bucket inconsistent with SPLASH bucket. Use SPLASH or SWBM bucket for NN. **0** = No consistent data.

mysitename	code
AR-SLu	2
AR-Vir	2
AT-Neu	2
AU-Ade	1
AU-ASM	1
AU-Cpr	3
AU-Cum	3

Due to its high computational demand when evaluating the profiling function for all 135 sites ("batch execution"), it can be executed in parallel for each site on a High Performance Computing cluster (We used the Imperial College London, HPC, server *CX1*). Note, that in this case, profile files are written into (and subsequently read from) `paste( myhome, "data/nn_fluxnet/profile/")` (see argument `testprofile` in various functions and scripts).

To do the batch execution, first, create a text file containing the list of sites for which profiling is to be done:

```
do.sites <- dplyr::filter( siteinfo, code!=0 )$mysitename
fileConn <- file("sitelist_doprofile_fluxnet2015.txt")
writeLines( do.sites, fileConn )
close(fileConn)
```

Then, in the terminal, enter

```
./submit_profile_nn_ALL.sh
```

This creates a job submission file for each site from `submit_profile_nn_XXXXX.sh` and submits it to the cluster by `qsub`. Note that the job submission file may have to be adjusted for execution on other clusters than Imperial HPC CX1.

4. Derive fLUE and identify drought periods

Purpose and steps

This step is implemented by function `nn_fVAR_fluxnet()`. It does the following:

1. Profile file (`./data/profile/profile_light_lue_obs_evi_nn_<sitename>.Rdata`) is read (if `testprofile==TRUE`) and data corresponding to the best performing soil moisture threshold is extracted from its large nested list.
2. Calculate fLUE time series for each soil moisture dataset and repetition and take the mean across repetitions. Code bit:

```
fvar <- apply(
  profile_nn[[ sitename ]][[ isoilm_data ]]$nnet$var_nn_all /
  profile_nn[[ sitename ]][[ isoilm_data ]]$nnet[[
    paste( "smtrh_", as.character( isoilm_trh ), sep="" )
    ]]$var_nn_bad,
```

```
  1, FUN=mean
  )
```

3. Remove outliers in fLUE (outside 3 times the interquartile range and greater than 1 or negative)

```
remove_outliers_fXX <- function( vec, coef=1.5 ) {
  ## use the command boxplot.stats()$out which use the Tukey's method
  ## to identify the outliers ranged above and below the <coef>*IQR.
  outlier <- boxplot.stats( vec, coef=coef )$out
  outlier <- outlier[ which( outlier>1.0 | outlier<0.0 ) ]
  vec[ which( is.element( vec, outlier ) ) ] <- NA
  return( vec )
}
df_nona$fvar <- remove_outliers_fXX( df_nona$fvar, coef=3.0 )
```

4. Take the mean across soil moisture datasets. Code bit:

```
nice$fvar <- apply( fvar_by_smdata, 1, FUN=mean, na.rm=TRUE )
```

5. Get fLUE cutoff to define threshold for fLUE droughts. The cutoff is calculated as the lower 5% quantile of a hypothetical symetrical distribution, taken as "mirrored" values in fLUE above 1. This is also illustrated by the histogram in the figure that is created below when executing `nn_fVAR_fluxnet()`.

```
positive <- nice$fvar[ which(nice$fvar>=1.0) ]
even <- c( positive, 1.0-(positive-1.0) )
cutoff <- quantile( even, 0.05, na.rm=TRUE )

## consider only instances where fvar falls below 0.97 as drought
cutoff <- min( 0.97, cutoff )
```

6. Interpolate missing values in fLUE

```
nice$fvar_filled <- approx( nice$year_dec, nice$fvar, xout=nice$year_dec )$y
```

7. Smooth fLUE data by taking running median across five days. The function `niceify()` is defined in file `niceify.R` and adds rows with NA values back to dataframes where these rows were removed before.

```
idxs <- which( !is.na(nice$fvar_filled) )
tmp <- data.frame( year_dec=nice$year_dec[idxs], fvar_smooth=runmed( nice$fvar_filled[idxs], 5 ) )
nice$fvar_smooth <- niceify( tmp, nice )$fvar_smooth
```

8. Interpolate missing values in smoothed time series.

```
nice$fvar_smooth_filled <- approx( nice$year_dec, nice$fvar_smooth, xout=nice$year_dec )$y
```

9. Classify days as fLUE drought days based on smoothed and interpolated time series. This adds a binary vector `is_drought_byvar` to the dataframe we're working with here (`nice`).

```
nice$is_drought_byvar <- ( nice$fvar_smooth_filled < cutoff )
```

10. Identify drought events as periods with consecutive drought days with a minimum length of 10 days. The function `get_consecutive()` (file `get_consecutive.R`) creates the dataframe `droughts` that stores, for each such drought event, the index (with respect to vector `nice$is_drought_byvar`) of its start and its length.

```
droughts <- get_consecutive(
                            nice$is_drought_byvar,
                            leng_threshold = 10,
                            do_merge       = FALSE
```

```
                              )
```

11. Prune and trim drought events:

- If the smoothed (and non-interpolated) fLUE vector (`nice$fvar_smooth`) has NA values at the end of a drought event, reduce the length of the event so that to last non-NA value.
- If the smoothed fLUE vector has NA values at the start of a drought event, drop the event.
- Drop drought event, if its mean soil moisture during the event is above the 75% quantile of all soil moisture values in this site's time series.
- Prune drought events by the quality of NN-modelled versus observed LUE during the drought period. I.e., if the RMSE of 0.9 * LUE_{act} is greater than the RMSE of LUE_{pot}.
- Prune drought if LUE_{pot} multiplied by PAR and fAPAR is below 5% of its maximum value. This removes erroneously classified droughts during winter in seasonally cold ecosystems. This is implemented by:

```
out <- prune_droughts(
                       droughts,
                       nice$is_drought_byvar,
                       nice$fvar_smooth,
                       nice$fvar_smooth_filled,
                       mod_pot    = nice$var_nn_pot,
                       mod_act    = nice$var_nn_act,
                       obs        = nice[[ nam_target ]],
                       soilm      = soilm,
                       soilm_mod  = soilm_mod,
                       apar       = nice$ppfd * nice[[ fapar_data ]]
                       )
```

Example execution

The function `nn_fVAR_fluxnet()` implements all steps outlined above and produces four figures, included below. The first shows a histogram of fLUE values (red), the hypothetical symetrical distribution (blue), and fLUE cutoff to delineate fLUE droughts as a red vertical line. The subsequent figures are modelled versus observed LUE of NN_{pot} during moist days, NN_{act} during all days and NN_{act} versus NN_{pot} during moist days.

```
source("nn_fVAR_fluxnet2015.R")
nn_fVAR_fluxnet( "FR-Pue", nam_target="lue_obs_evi", use_weights=FALSE, makepdf=FALSE, testprofile=TRUE
```

```
## Loading required package: dplyr

##
## Attaching package: 'dplyr'

## The following objects are masked from 'package:stats':
##
##     filter, lag

## The following objects are masked from 'package:base':
##
##     intersect, setdiff, setequal, union

## Loading required package: abind

## [1] "loading profile file ./data/profile/profile_light_lue_obs_evi_nn_FR-Pue.Rdata"
## [1] "=============================================="
## [1] "train NN at lue_obs_evi for FR-Pue ..."
## [1] "Using soil moisture data source: soilm_swbm"
## [1] "Using soil moisture threshold: 0.55"
```

```
## [1] "reading from profile data..."
## [1] "... done."
## [1] "aggregate across soil moisture datasets ..."
## [1] "done ..."
## [1] "get cutoff ..."
## [1] "done ..."

## [1] "fill and smooth ..."
## [1] "done ..."
## [1] "get drought events ..."
## [1] "number of events before pruning: 16"
## [1] "number of events after trimming: 16"
## [1] "number of events after pruning by soil moisture: 16"

## Loading required package: hydroGOF

## Loading required package: zoo

##
## Attaching package: 'zoo'

## The following objects are masked from 'package:base':
##
##     as.Date, as.Date.numeric

## [1] "number of events after pruning by mod vs obs: 15"
## [1] "number of events after pruning by GPP level: 15"
## [1] "number of events after pruning: 15"

##
## Attaching package: 'Metrics'

## The following objects are masked from 'package:hydroGOF':
##
##     mae, mse, rmse
```

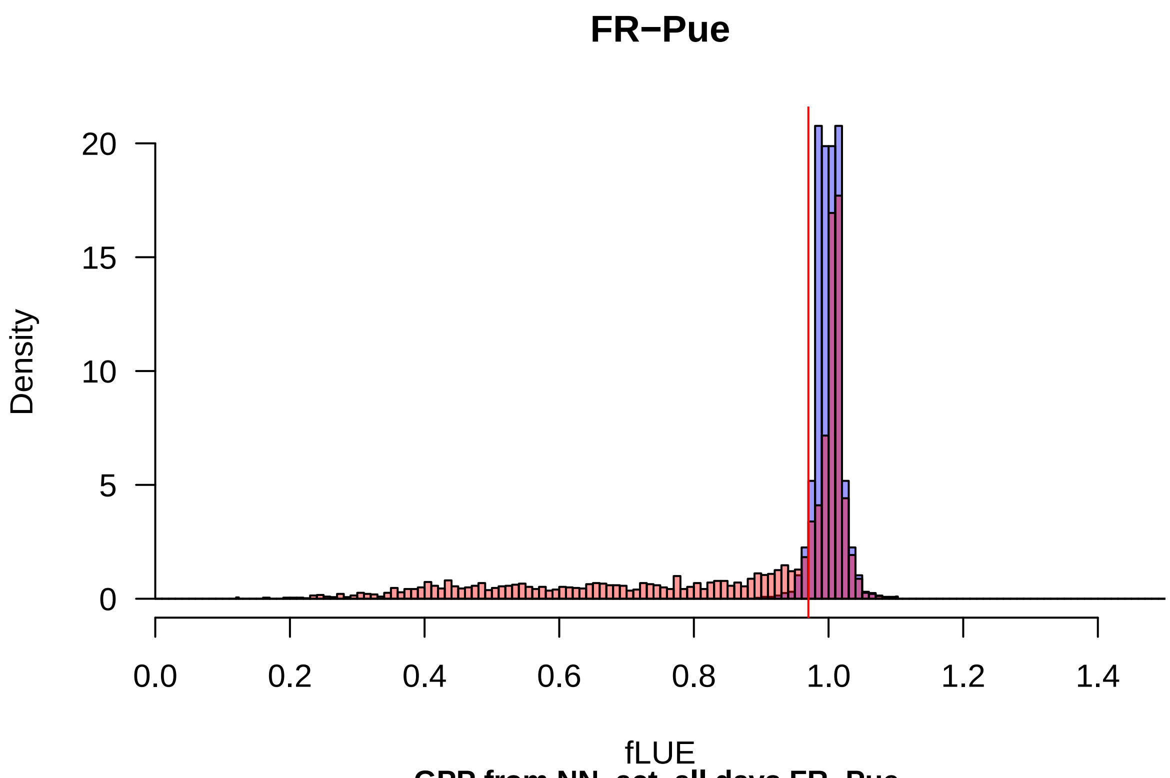
FR−Pue
Density
fLUE
0
5
10
15
20
0.0
0.2
0.4
0.6
0.8
1.0
1.2
1.4

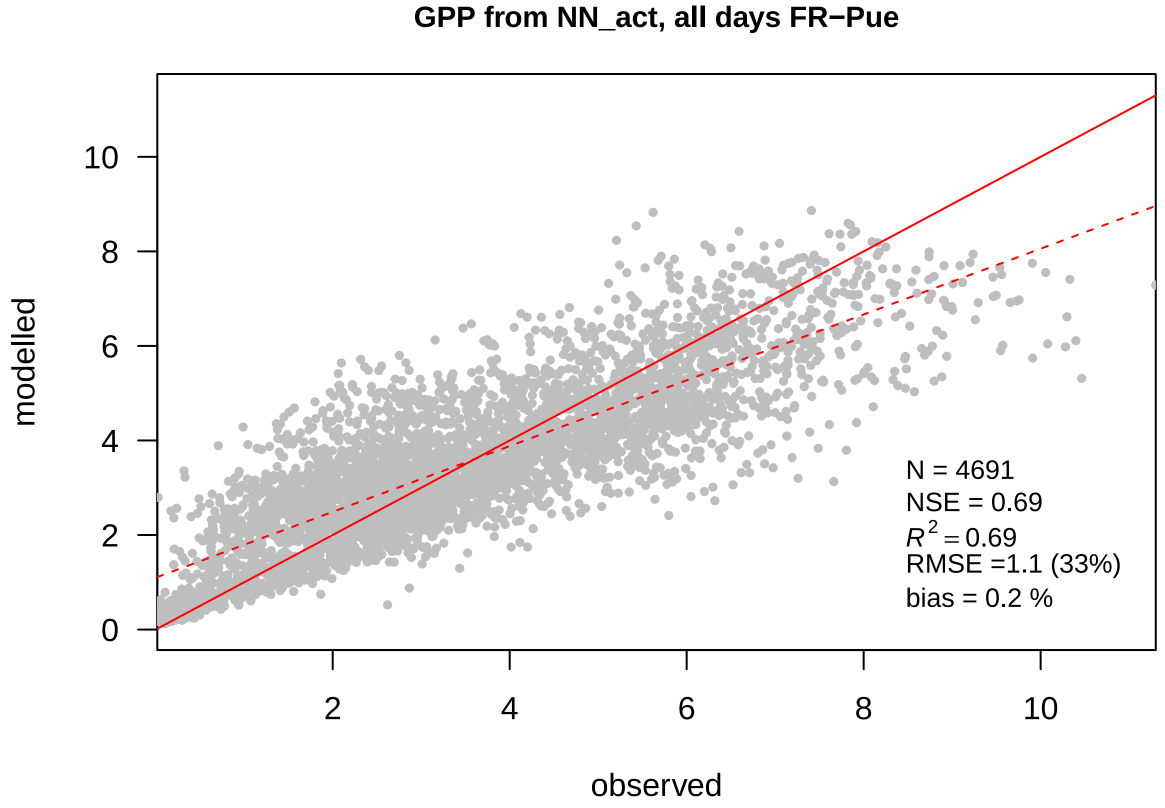
GPP from NN_act, all days FR−Pue
modelled
observed
0
2
4
6
8
10
2
4
6
8
10
N = 4691
NSE = 0.69
$R^2 = 0.69$
RMSE =1.1 (33%)
bias = 0.2 %

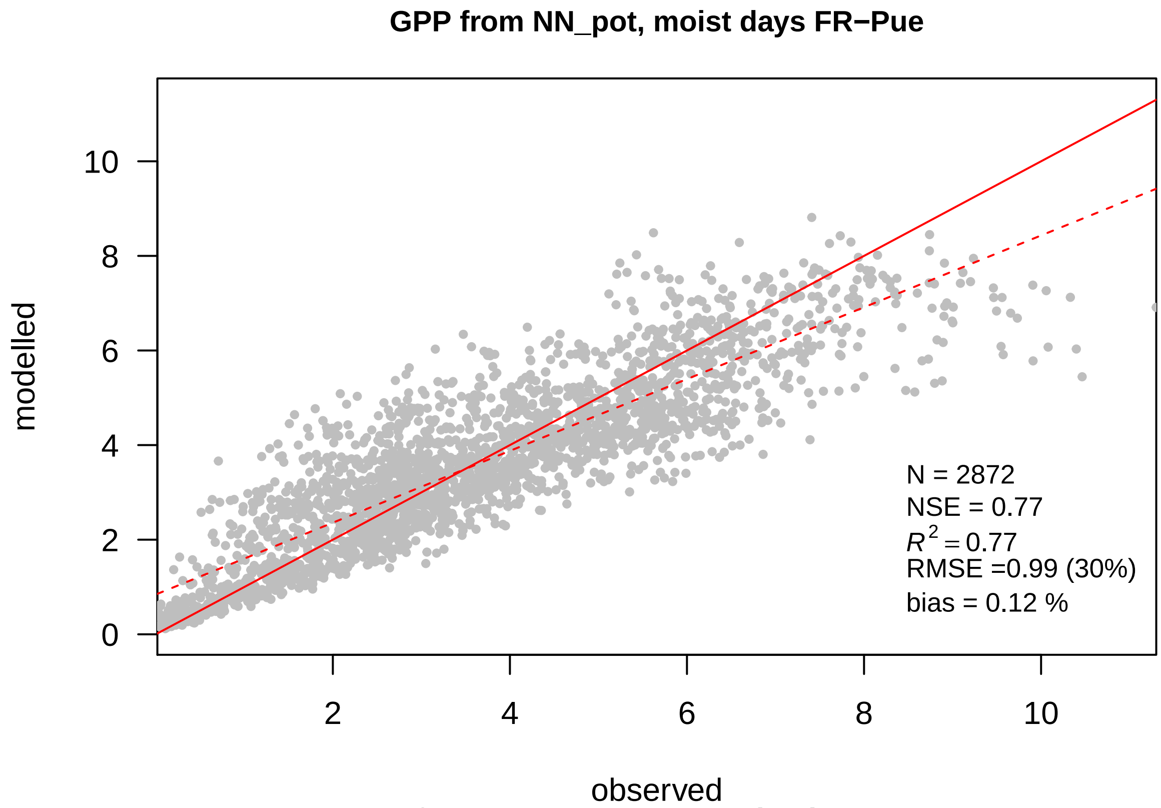

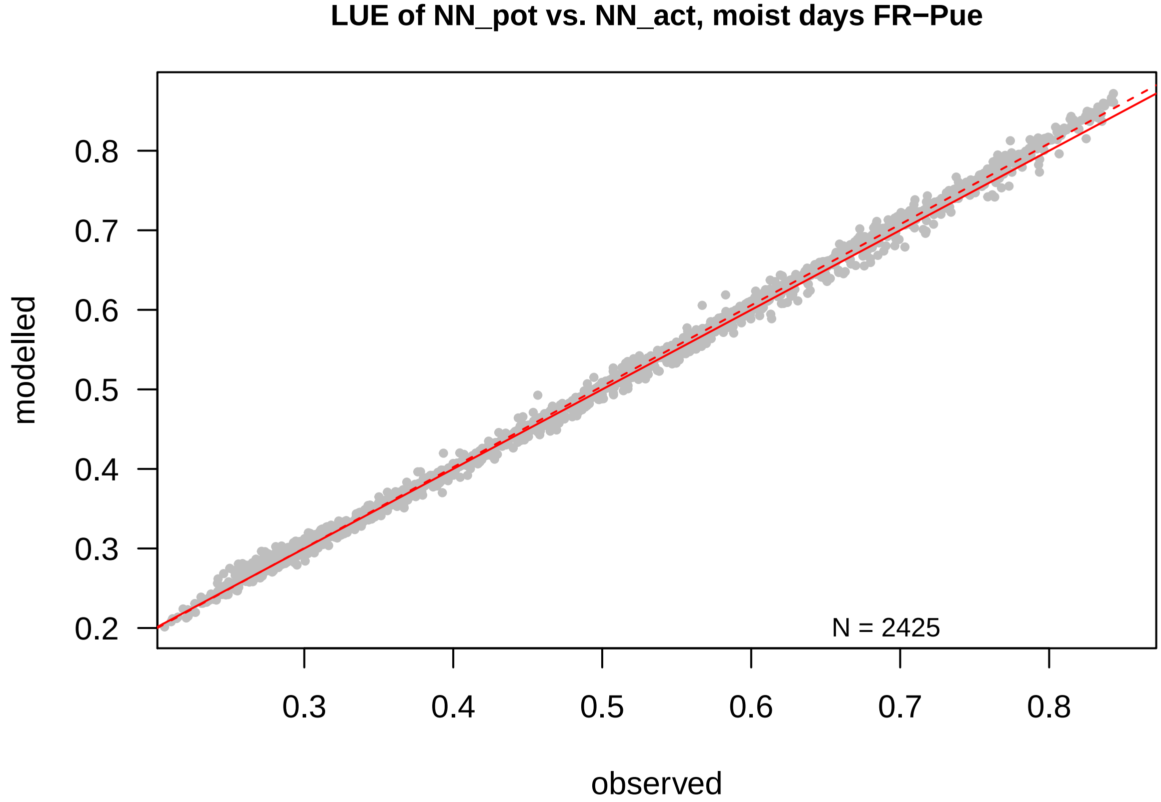


```
## [1] "writing file with fLUE and all other data into: data/nn_fluxnet2015_FR-Pue_lue_obs_evi.Rdata"
```

Complete batch execution

Parallel execution on a HPC cluster of `nn_fVAR_fluxnet()` for each site separately is implemented by `submit_fVAR_nn_ALL.sh`, `submit_fVAR_nn_XXXXXX.sh` and `execute_nn_fVAR_fluxnet2015.R`. Submit all site-specific jobs (site list in file `sitelist_doprofile_fluxnet2015.txt`, as created above) to cluster by entering the following command in the terminal:

```
./submit_fVAR_nn_ALL.sh
```

5. Quality check

Sites are excluded from further analysis based on data length and performance statistics of the NN model goodness:

1. if the number of days' data used for NN training (after data cleaning, see above) is below 500.
2. if the mean of LUE_{act} during fLUE drought days is greater than that of LUE_{pot}
3. if the RMSE is greater than 2.8 or R^2 is smaller than 0.5 for LUE_{act} compared with LUE_{obs}.
4. if the percentage RMSE (pRMSE) is greater than 80% or R^2 is smaller than 0.3 for LUE_{pot} compared with LUE_{obs} during non-fLUE drought days only. pRMSE is calculated by function `analyse_modobs()` (script `analyse_modobs.R`) as RMSE (root mean square error) divided by the mean of observationsand multiplied by 100.
5. if LUE_{act} has an R^2 of less than 0.17 during fLUE drought days.
6. if the R^2 of LUE_{act} versus LUE_{pot} during non-fLUE drought days is below 0.5 or their RMSE is above 1.6.

Sites excluded upon visual inspection and for which no quantitative criteria are found, are: CN-Din, CZ-BK1, IT-CA1, IT-CA3, IT-Ro2, and US-Me6.

This is implemented by function `nn_getfail_fluxnet()` which returns a dataframe (one row) with all the performance metrics for this site.

```r
source("function_nn_getfail_fluxnet2015.R")
out <- nn_getfail_fluxnet( "FR-Pue", nam_target="lue_obs_evi",
                           use_weights=FALSE, use_fapar=FALSE,
                           testprofile=TRUE, makepdf=FALSE, verbose=TRUE
                           )
```

```
## [1] "reading file ./data/fvar/nn_fluxnet2015_FR-Pue_lue_obs_evi.Rdata"
```

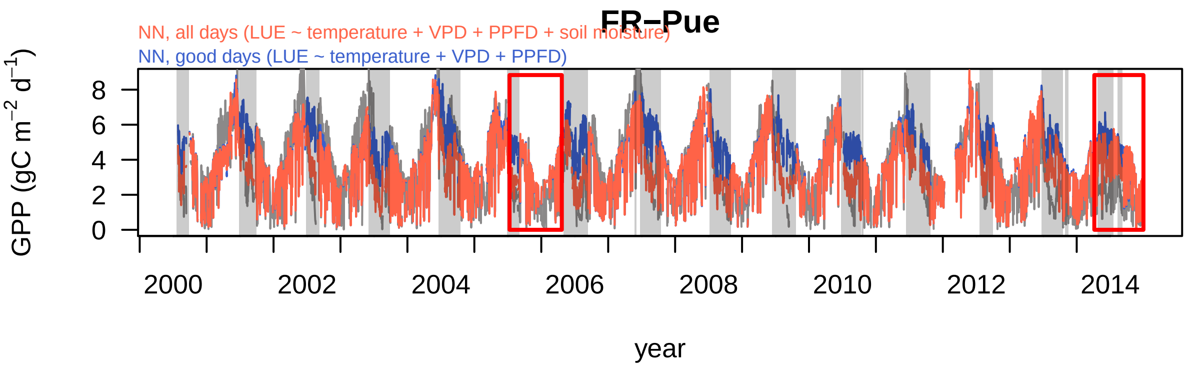

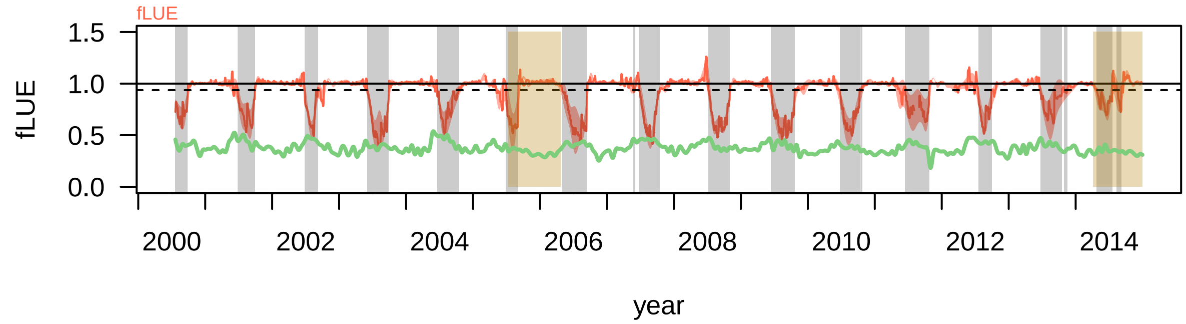

The column `successcodes` provides the outcome of the quality check as a code.

```
print( dplyr::select( out, mysitename, successcode ) )
```

```
##   mysitename successcode
## 1     FR-Pue           1
```

successcode	meaning
1	success, droughts identified
2	success, no droughts identified (fLUE drought days make up less than 2% of all days.)
3	failed quality check

Batch quality check

Up to this point, all steps are carried out for one single example site and outputs are written into and read from `./data`. In order to enable further analyses and make the study fully reproducible, we provide all site-specific "intermediate" files created by function `nn_fVAR_fluxnet()` that stores data, including fLUE. This avoids having to do the batch execution of `profile_soilm_neuralnet()` and `nn_fVAR_fluxnet()`. Data can be downloaded and extracted as follows (uncomment these lines in file `knit_nn_fluxnet2015.Rmd`):

Once data is downloaded and extracted, get the success code and performance statistics for all sites in one dataframe and write it to the file `./successcodes.csv`.

```
source( "nn_getfail_fluxnet2015.R" )
```

```
## [1] "getting failure info for all sites ..."

## [1] "... done"
## [1] "Force exclusion of the following sites: "
## [1] "CN-Din" "CZ-BK1" "IT-CA1" "IT-CA3" "IT-Ro2" "US-Me6"
## [1] "number of sites with code 1: 36"
```

```
## [1] "number of sites with code 2: 35"
## [1] "number of sites with code 3: 63"
## [1] "number of sites with code 0: 32"
```

Out of the 135 sites for which the profiling was carried out (consistent soil moisture time series), 76 sites have a success code of 1 or 2, i.e. the NN-based method to quantify fLUE and identify fLUE droughts worked acceptably. These 76 sites are used for all subsequent analyses and are finally assigned to the four clusters.

6. Evaluations of full time series by site

Statistics for overview table

Create a table that stores all the statistics evaluated for each site and that is finally used to create plot S5 of the paper. It is created here and complemented by additional evaluations in subsequent steps.

```
source( "get_overview_L1.R" )
```

```
## [1] "Getting data for overview table for all sites ..."
## [1] "... done."
## [1] "Saving to file data/overview_data_fluxnet2015_L1.Rdata"
```

```
load( "data/overview_data_fluxnet2015_L1.Rdata" )
knitr::kable( head( select(overview, mysitename, lon, lat, elv, year_start, year_end, years_data, class
```

Table 3: **Table**: First seven rows and first nine columns of overview table.

mysitename	lon	lat	elv	year_start	year_end	years_data	classid
AR-SLu	-66.4598	-33.4648	887	2009	2011	3	MF
AR-Vir	-56.1886	-28.2395	74	2009	2012	4	ENF
AT-Neu	11.3175	47.1167	970	2002	2012	11	GRA
AU-Ade	131.1178	-13.0769	188	2007	2009	3	WSA
AU-ASM	133.2490	-22.2830	615	2010	2013	4	ENF
AU-Cpr	140.5891	-34.0021	51	2010	2014	5	SAV
AU-Cum	150.7225	-33.6133	107	2012	2014	3	EBF

Fit functional form: fLUE versus soil moisture

Fit a quadratic function to the relationship between fLUE and soil moisture for each site. This creates figures in `./fig_nn_fluxnet2015/fvar_vs_soilm/`, and stores data for all sites in `data/fvar_vs_soilm.Rdata` (medians of fLUE by soil moisture 20%-quantiles) and `data/fitparams.Rdata` (fit parameters of quadratic fit function). This also complements the overview table, stored as `data/overview_data_fluxnet2015_L2.Rdata`. The figures included below are three examples of the fit figures created for all sites (see Figure S2 in SI of paper).

```
system( "mkdir -p ./fig_nn_fluxnet2015/fvar_vs_soilm" )
source( "fit_fvar_vs_soilm_nn_fluxnet2015.R" )
```

```
## [1] "Fitting functional relationship for all sites ..."
```

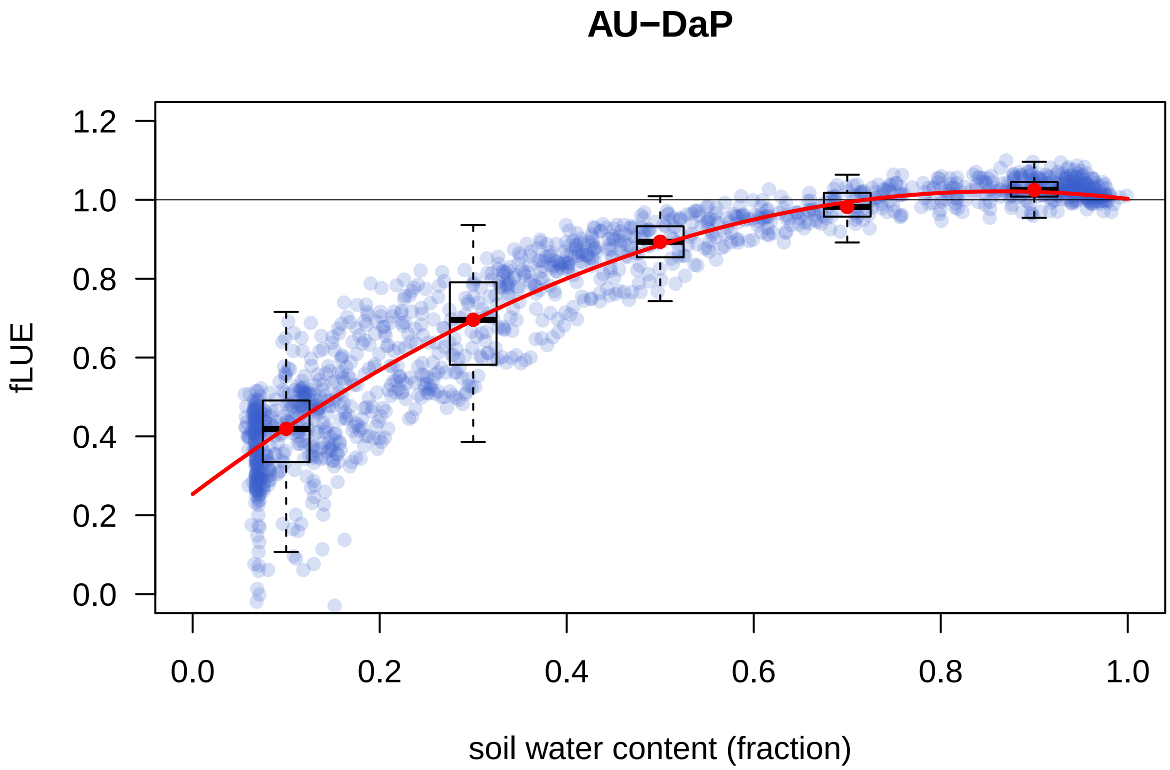
AU−DaP
fLUE
1.2
1.0
0.8
0.6
0.4
0.2
0.0
0.0
0.2
0.4
0.6
0.8
1.0
soil water content (fraction)

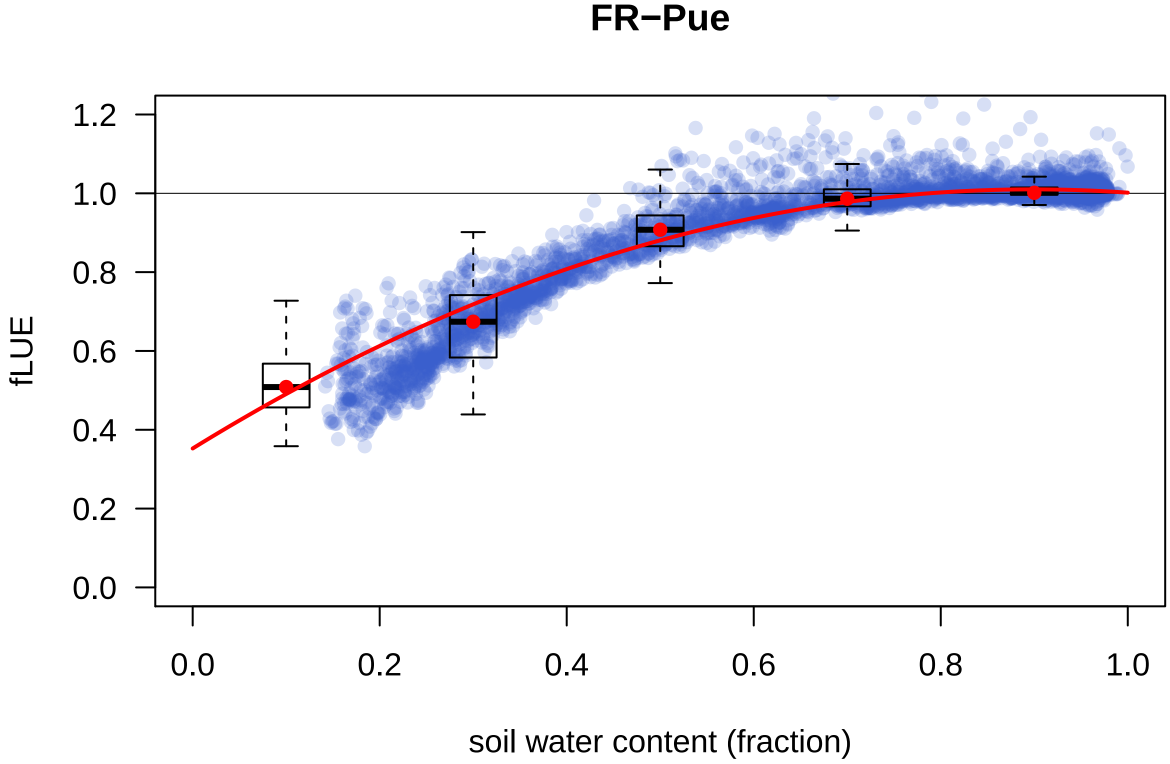
FR−Pue
fLUE
1.2
1.0
0.8
0.6
0.4
0.2
0.0
0.0
0.2
0.4
0.6
0.8
1.0
soil water content (fraction)

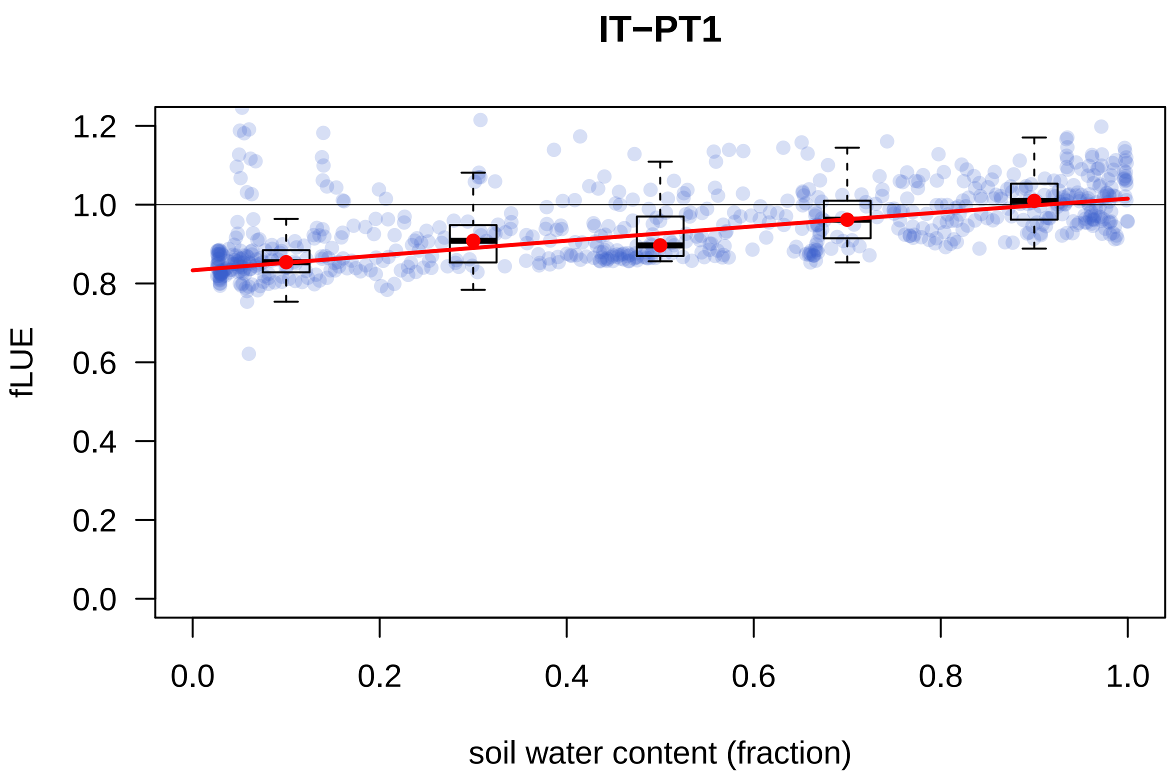


```
## [1] "... done."
```

Aggregate data across sites

This aggregates site-specific data into one big dataframe holding all sites' data (used for additional plotting) as `./data/nice_agg_lue_obs_evi.Rdata`

```
source( "aggregate_nn_fluxnet2015.R" )
```

Create plots

This creates the time series plot, Fig. 1 in the manuscript.

```
source( "plot_bysite_nn_fluxnet2015.R" )
plot_bysite_nn_fluxnet2015( "FR-Pue", nam_target="lue_obs_evi",
                            use_fapar=FALSE, use_weights=FALSE,
                            makepdf=FALSE
                            )
```

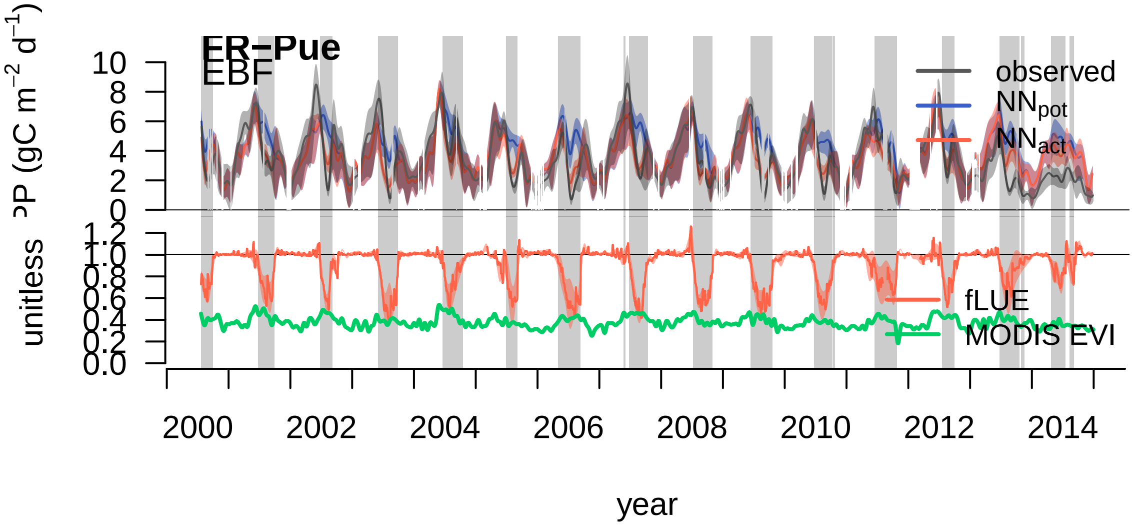

To get plots for all sites, do

```
system( "mkdir -p ./fig_nn_fluxnet2015/panel_potentialgpp/" )
source( "execute_plot_bysite_nn_fluxnet2015.R" )
```

```
## [1] "creating time series plots for all sites ..."
## [1] "... done."
```

7. Evaluations of aligned variables by site

Reshape data

First, for each site separately, time series of variables stored in `nice` (file `nn_fluxnet2015_<sitname>_lue_obs_evi.Rdata`) are "cut" and aligned by the onset of fLUE droughts. Thereby, droughts can be characterised in terms of the common (aggregated across drought events) co-evolution of different variables. This creates a new data array `data_alg_dry` with dimensions (dday, var, inst), where 'dday' is the number of days after drought onset, `var` are the variables (columns) in `nice`, and `inst` is the drought instance. All arrays are stored as `./data/aligned_<sitename>.Rdata`.

Second, `data_alg_dry` is re-expanded to a dataframe (2D) `df_dday` and data is binned with respect to `dday`. This step is also done for dataframes with MODIS GPP and FLUXCOM MTE data (`df_dday_modis` , `df_dday_mte`) and all are saved in files (`./data/aligned_<sitename>.Rdata`, `./data/aligned_mte_<sitename>.Rdata`, and `./data/aligned_modis_<sitename>.Rdata`)

Third, `df_dday` is aggregated (median and upper and lower quartiles) with respect to `dday`, leading to dataframe `df_dday_aggbydday` and saved in `data/df_dday_aggbydday_<sitename>.Rdata`.

The function `reshape_align_nn_fluxnet2015()` executes above steps and returns a list containing the dataframes `df_dday` and `df_dday_aggbydday`:

```
source( "reshape_align_nn_fluxnet2015.R" )
out <- reshape_align_nn_fluxnet2015( "FR-Pue" )
```

```
## Loading required package: cgwtools
```

```
print( "content of returned list:" ); ls( out )
```

```
## [1] "content of returned list:"
## [1] "df_dday"           "df_dday_aggbydday"
```

The script `execute_reshape_align_nn_fluxnet2015()` executes this function for all sites where fLUE droughts are identified (`successcode==1`) and combines dataframes into combined large dataframes containing all sites' data, saved in `data/data_aligned_agg.Rdata`:

- `df_dday_agg`
- `df_dday_aggbydday_agg`

```
source( "execute_reshape_align_nn_fluxnet2015.R" )
```

```
## [1] "aligning data for all sites ..."
## [1] "... done."
## [1] "saving to file: data/data_aligned_agg.Rdata"
```

Create plots

Panel plots for soil moisture, VPD, fLUE and EVI, all aligned by the fLUE drought onset and aggregated across drought events (Fig. 2 in paper main text), are created by the function `plot_aligned_nn_fluxnet2015()`:

```
source( "plot_aligned_nn_fluxnet2015.R" )
plot_aligned_nn_fluxnet2015( "FR-Pue", nam_target="lue_obs_evi",
                            use_fapar=FALSE, use_weights=FALSE, makepdf=FALSE
                            )
```

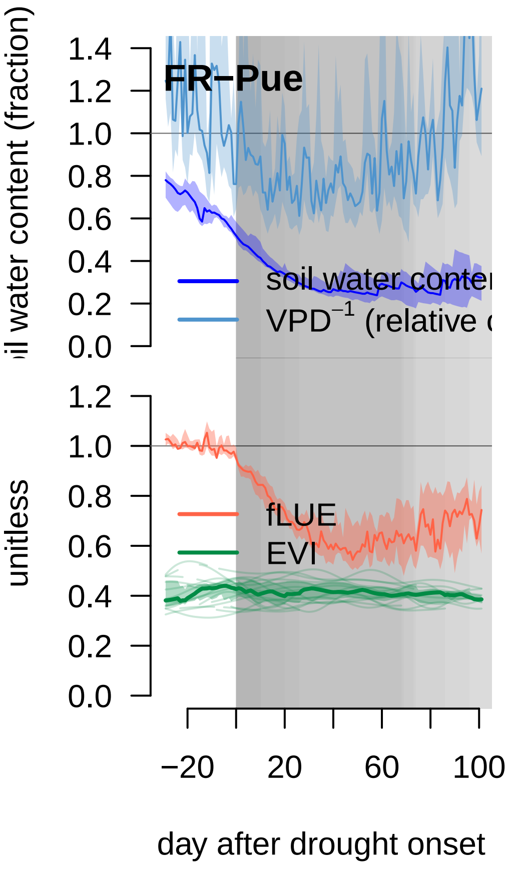

Create panel plots for all sites and save to PDFs by doing:

```
source( "execute_plot_aligned_nn_fluxnet2015.R" )
```

```
## [1] "create aligned plots for all sites ..."

## [1] "... done."
```

8. Clustering sites

Step 1

In step 1, sites are grouped into clusters cLS and cNA:

- **cluster cNA**: Sites not affected by very low soil moisture, i.e. where no data is available for soil moisture below 0.5 (relative soil water content).
- **cluster cLS**: Sites where the fLUE is not sensitive to soil moisture. This is determined based on the fitted functional form between soil moisture and fLUE (quadratic function) and the y-axis cutoff thereof (referred to as $fLUE_0$, see Section 'Fit functional form: fLUE versus soil moisture'). The distribution of $fLUE_0$ values (by site) is shown in the figure below. Cluster 3 are sites with high $fLUE_0$, generally above 0.8 (green bars in histogram plotted below).

This uses data from files `data/fvar_vs_soilm.Rdata` and `data/fitparams.Rdata`, created by script `fit_fvar_vs_soilm_nn_fluxnet2015.R` and complements overview table, creating `data/overview_data_fluxnet2015_L4.R`

```
source("cluster_step1_nn_fluxnet2015.R")
```

```
## Warning: package 'ggplot2' was built under R version 3.3.2

## [1] "Total number of sites in group 4 (no low soil moisture): 21"

## Warning in left_join_impl(x, y, by$x, by$y, suffix$x, suffix$y): joining
## factor and character vector, coercing into character vector
```

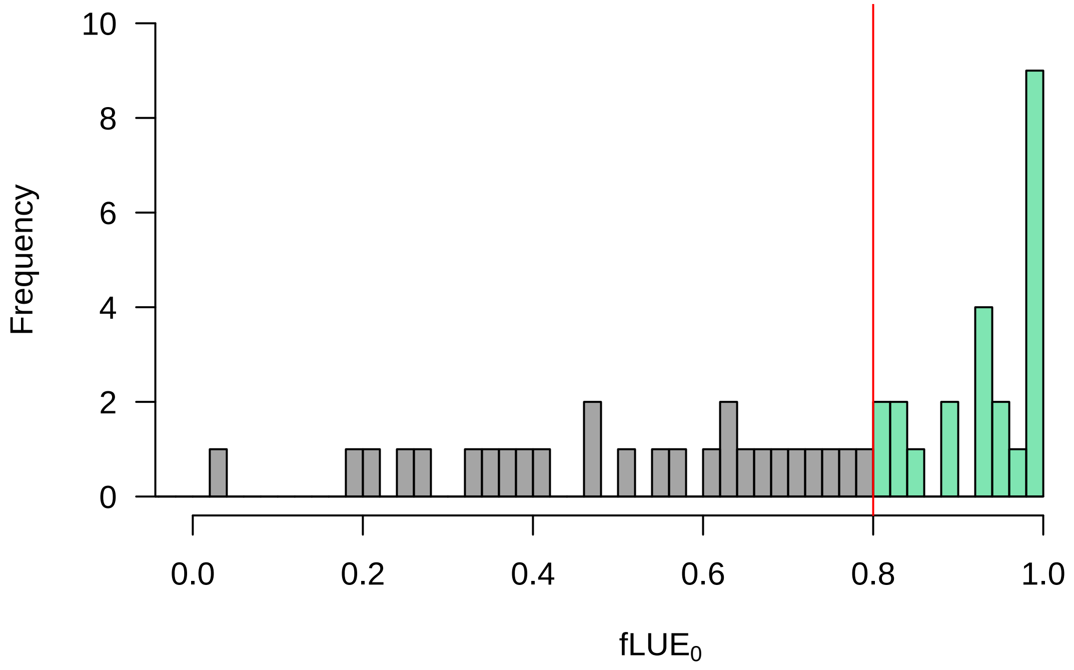


```
## [1] "Total number of sites in group 3 (low fLUE sensitivity): 23"
```

Step 2

Remaining sites are grouped by their parallel greenness and fLUE response during droughts.

- **cluster cGR**: No (or very small) greenness change and modest fLUE change
- **cluster cDD**: Pronounced greenness change and strong fLUE decline

This is based on aligned and binned data, as illustrated in the figure below, where the lines represent medians of data aligned by drought events, boxes represent values binned into 20 day periods (1 before and 2 after

drought onset), green dots are medians of binned EVI data, normalised with respect to the level in bin 1 (EVI/EVI_0) and red dots are medians of binned fLUE data.

```
if (!file.exists("fig_nn_fluxnet2015/aligned_binned"))
  system("mkdir fig_nn_fluxnet2015/aligned_binned")
source("get_aggresponse_binned.R")
out <- get_aggresponse_binned( "FR-Pue", makepdf = FALSE )
```

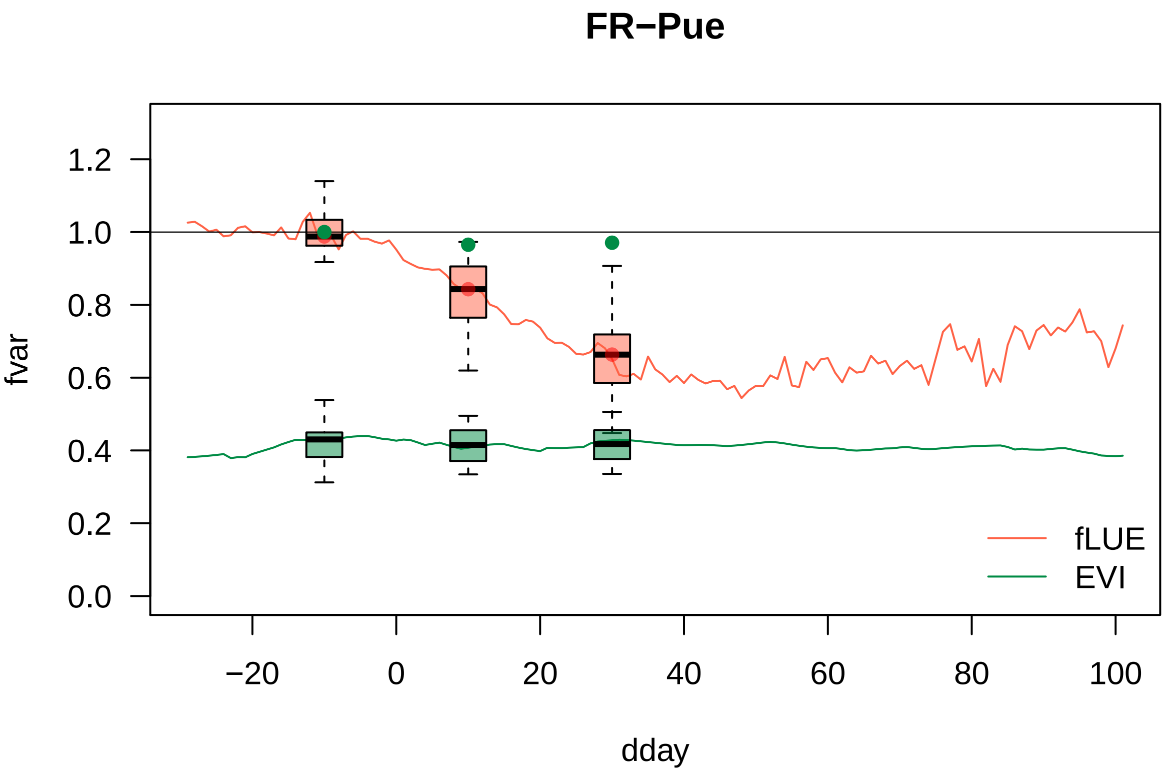

The clustering into clusters cDD and cGR is based on the medians of binned data ('fapar_agg' and 'fvar_agg' in code, dots in above figure for one site) and is implemented using the `kmeans()` function from the 'stats' R package:

```
## Combine variables based on which clustering is done into a single array 'mega'
mega <- cbind( fapar_agg, fvar_agg )
set.seed(1)
outkmeans <- stats::kmeans( mega, 2 )
```

The script `cluster_step2_nn_fluxnet2015.R` executes both the collecting of binned and aligned data for all sites and the clustering. It complements the overview table and creates `data/overview_data_fluxnet2015_L5.Rdata`.

```
source("cluster_step2_nn_fluxnet2015.R")
```

```
## [1] "total number of sites left for remaining clusters: 26"

## [1] "The following figure shows the dependence of the total within-cluster sum of squared difference
```

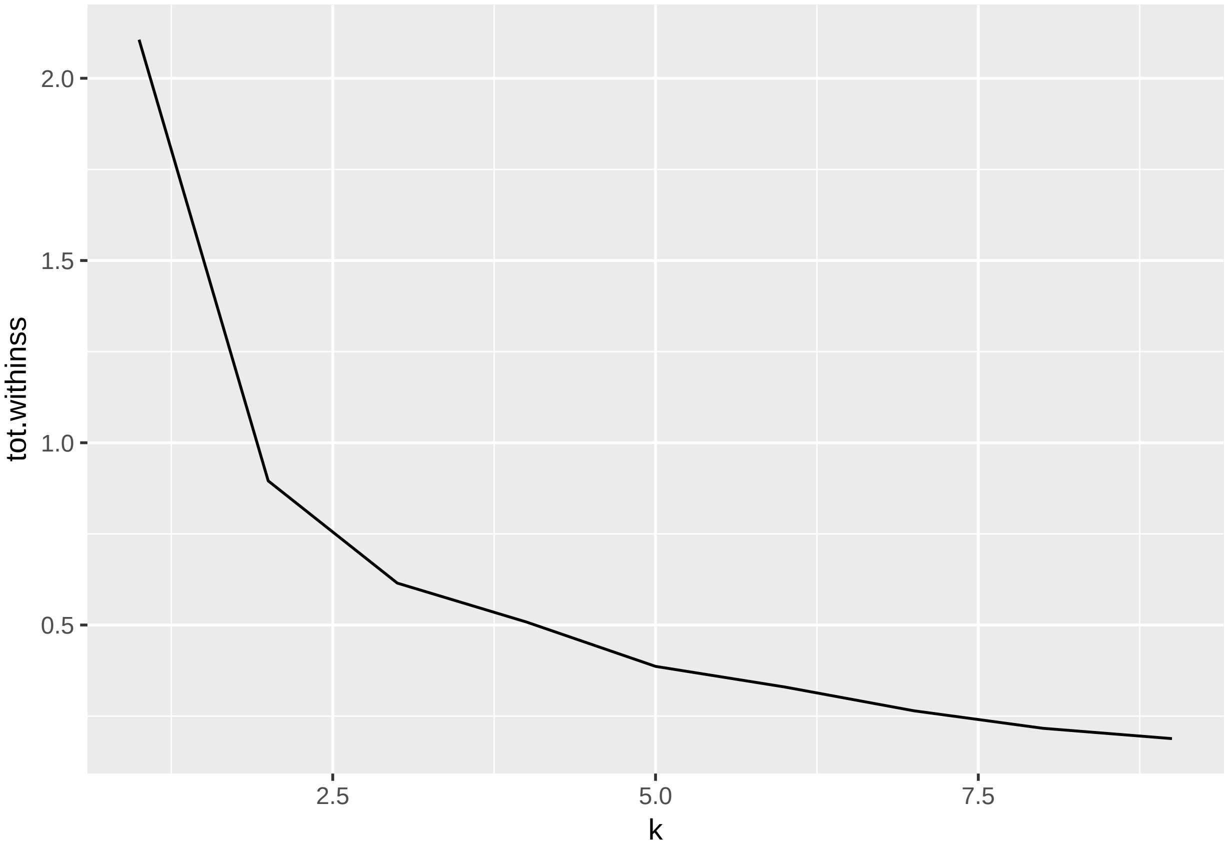


```
## [1] "The following figure shows a histogram of the greenness changes (median of third bin, representi
```

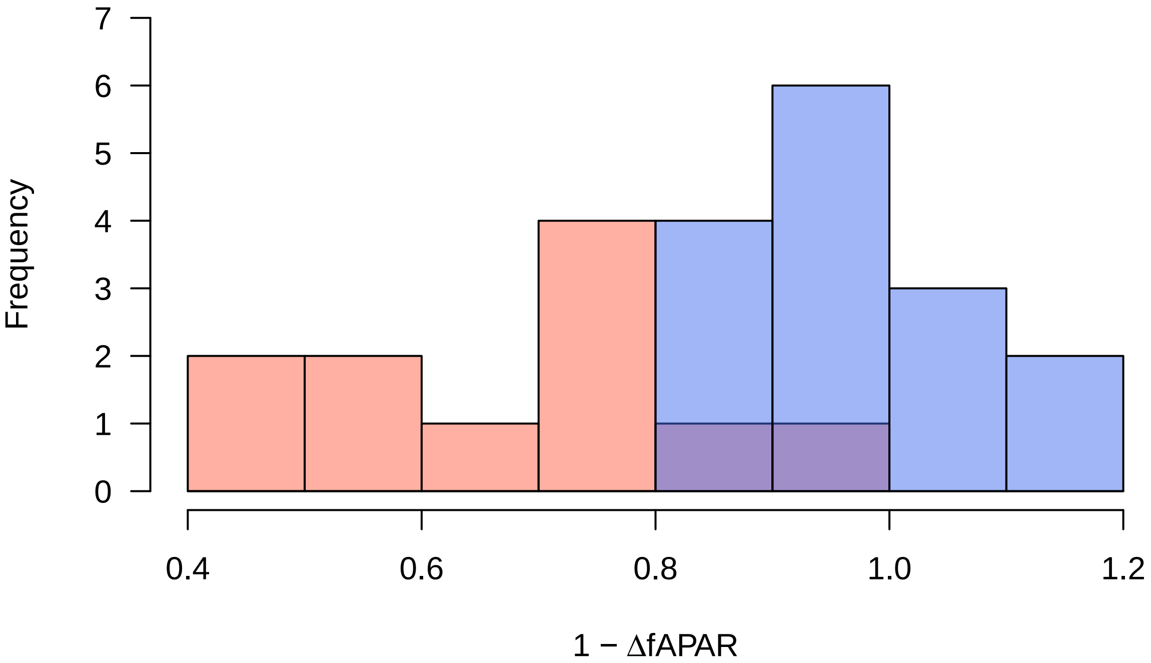

9. Create figures

At this point, all analyses are completed. Remaining steps create (remaining) figures used in the paper.

Evaluate pooled data

The script `plot_agg_nn_fluxnet2015.R` produces the following figures:

- `fig_nn_fluxnet2015/boxplot_fluedrought_vs_alpha_spi_spei_1mo.pdf`: Fig. 8 in paper. Boxplots for SPI, SPEI, and AET/PET distributions during fLUE droughts and non-droughts.
- `fig_nn_fluxnet2015/modobs/modobs_gpp_rct_ALL_FROMNICE.pdf`: Fig. 2 in paper. Modelled versus observed scatterplots, evaluating NN performance based on GPP.
- `fig_nn_fluxnet2015/modobs/modobs_lue_rct_ALL_FROMNICE.pdf`: Fig. S3 in Supplementary Information. Modelled versus observed scatterplots, evaluating NN performance based on LUE
- `fig_nn_fluxnet2015/fpar_vs_evi.pdf`: Fig. S1 in Supplementary Information. Scatterplot of MODIS FPAR versus EVI.

```
source("plot_agg_nn_fluxnet2015.R")
```

```
## [1] "plotting fig_nn_fluxnet2015/boxplot_fluedrought_vs_alpha_spi_spei_1mo.pdf"

## [1] "plotting mod vs obs for GPP in nice_agg: fig_nn_fluxnet2015/modobs/modobs_gpp_rct_ALL_FROMNICE.

## [1] "plotting mod vs obs for LUE in nice_agg: fig_nn_fluxnet2015/modobs/modobs_lue_rct_ALL_FROMNICE.

## [1] "plotting  fig_nn_fluxnet2015/fpar_vs_evi.pdf"
```

Evaluate pooled data by clusters

The script `plot_bycluster_nn_fluxnet2015.R` produces the following figures:

- `./fig_nn_fluxnet2015/vpd_vs_soilm/vpd_vs_soilm_lue_obs_evi_ALL.pdf`: Fig. S6 in Supplementary Information. Scatterplot of VPD versus soil moisture and boxplot of VPD distribution during dry and moist days.
- `fig_nn_fluxnet2015/fvar_vs_soilm/fvar_vs_soilm_agg_ALL.pdf`: Fig. 7 in the main text of the paper. Functional form of fLUE versus soil moisture, by cluster.
- `fig_nn_fluxnet2015/aligned_cluster/aligned_cluster_3rows.pdf`: Fig. 3 in the main text of the paper. fLUE and EVI/EVI_0 during fLUE droughts for clusters 1 and 2.

```
source("plot_bycluster_nn_fluxnet2015.R")
```

```
## [1] "plotting ./fig_nn_fluxnet2015/vpd_vs_soilm/vpd_vs_soilm_lue_obs_evi_ALL.pdf"

## [1] "plotting fig_nn_fluxnet2015/fvar_vs_soilm/fvar_vs_soilm_agg_ALL.pdf"

## [1] "printing fig_nn_fluxnet2015/aligned_cluster/aligned_cluster_3rows.pdf"
```

Evaluate clusters (climate, vegetation type, etc.)

Overview "heat table" (Fig. S5)

The script `plot_overview_nn_fluxnet2015.R` combines the overview table (`data/overview_data_fluxnet2015_L5.Rdata`) with complementary site data extracted from global datasets:

- Water table depth from Fan & Miguez-Macho (2013). Dataframe `df_wtd` from `./data/wtd_fluxnet2015.Rdata`. Data prepared by `get_watertable_fluxnet2015.R`. Original files downloaded on May 16, 2017 from glowasis.deltares.nl, selecting `/thredds/fileServer/opendap/opendap/Equilibrium_Water_Table/Eurasia_model_w`
- Water table depth from (Graaf et al. 2015). Dataframe `df_wtd_degraaf` from `data/wtd_degraaf_fluxnet2015.Rdata`. Data prepared by `get_watertable_degraaf_fluxnet2015.R`. Original file provided by I. De Graaf (29 May 2017).
- Budyko regime (energy limited, water limited, transitional) from (Greve et al. 2014–10AD). Dataframe `df_greve` from `./data/greve_fluxnet2015.Rdata`. Data prepared by `get_greve.R`. Original file provided by P. Greve.

- Aridity index. Dataframe `df_ai` from `./data/ai_fluxnet2015.Rdata`. Data prepared by `get_aridityindex.R` based on SPLASH model outputs from simulation s13 (see above).
- Average actual over potential evapotranspiration (AET/PET). Dataframe `df_alpha` from `./data/alpha_fluxnet2015.Rdata`. Data prepared by `get_alpha.R` based on SPLASH model outputs from simulation s11 (see above).
- Soil parameters from HWSD, data by (Shangguan et al. 2014). Dataframe `df_soil` from `./data/soilparams_fluxnet2015.Rdata` prepared by `get_soilparams.R`. Original files downloaded from http://globalchange.bnu.edu.cn/research/soilwd.jsp (26 May 2017). Complemented overview table is saved as `data/overview_data_fluxnet2015_L6.Rdata`.

The script produces the following figures:

- `fig_nn_fluxnet2015/overview_clusterX.pdf` where `X` is 1, 2, 3 and 4. This is the colored overview table of all sites, grouped by cluster. Fig. S5 in Supplementary Information

This script also evaluates the correlation of water table depth site data from (Fan, Li, and Miguez-Macho 2013) compared with (Graaf et al. 2015).

```
source("plot_overview_nn_fluxnet2015.R")
```

```
## [1] "Correlation of water table depth site data from Fan & Miguez-Macho (2013) compared with De Graa
## [1] "adjusted R2: 0.224755326360854"
## [1] "Correlation of fLUE_0 with water table depth, site data from Fan & Miguez-Macho (2013)"
## [1] "adjusted R2: -0.0196119167708804"
## [1] "Correlation of fLUE_0 with water table depth, site data from De Graaf et al. (2015)"
## [1] "adjusted R2: 0.0109571701227021"
```

Sites across climate space (Fig. 9)

The script `plot_sites_climatespace.R` creates a panel plot, illustrating the locations of sites in Budyko space and the relationship of annual percent GPP loss and maximum LUE reduction with AET/PET and the change in greenness. The figure `sites_climatespace.pdf` is used in the paper as Fig. 9

```
source("plot_sites_climatespace.R")
```

```
## [1] "plotting fig_nn_fluxnet2015/sites_climatespace.pdf"
```

Map of PET/P and sites locations (Fig. 10)

The script `plot_map_greve.R` creates a global map of PET/P (=aridity index) with symbols illustrating site locations, including distributions of the aridity index and AET/PET by clusters. This is Fig. 10 in paper. Plotting the map requires loading the NetCDF file that contains global data on PET/P from (Greve et al. 2014–10AD). However, this is not made publicly available. Therefore, the script just plots a constant field with value 1 as a map. The figure `map_greve.pdf` is used in the paper as Fig. 10.

```
source("plot_map_greve.R")
```

```
## [1] "plotting ./fig_nn_fluxnet2015/map_greve.pdf"
```

```
## [1] "MYCOLORBAR: assuming explicit margins provided"
```

Evaluate NN sensitivity to VPD

The script `evaluate_sensitivity_soilm_vpd.R` evaluates the sensitivity of NNs to VPD and the bias of their predictions of LUE under moist and dry conditions. Figure `vpd_soilmoisture_test.pdf` is created and used in the paper as Fig. 3. Execution of this script requires the full profile files. Due to their large size, they are not distributed here and the script thus cannot be executed.

```
source("evaluate_sensitivity_soilm_vpd.R")
```

References

Davis, T. W., I. C. Prentice, B. D. Stocker, R. T. Thomas, R. J. Whitley, H. Wang, B. J. Evans, A. V. Gallego-Sala, M. T. Sykes, and W. Cramer. 2017. "Simple Process-Led Algorithms for Simulating Habitats (Splash V.1.0): Robust Indices of Radiation, Evapotranspiration and Plant-Available Moisture." *Geoscientific Model Development* 10 (2): 689–708. doi:10.5194/gmd-10-689-2017.

Fan, Y., H. Li, and G. Miguez-Macho. 2013. "Global Patterns of Groundwater Table Depth." *Science* 339 (6122). American Association for the Advancement of Science: 940–43. doi:10.1126/science.1229881.

Graaf, I. E. M. de, E. H. Sutanudjaja, L. P. H. van Beek, and M. F. P. Bierkens. 2015. "A High-Resolution Global-Scale Groundwater Model." *Hydrology and Earth System Sciences* 19 (2): 823–37. doi:10.5194/hess-19-823-2015.

Greve, Peter, Boris Orlowsky, Brigitte Mueller, Justin Sheffield, Markus Reichstein, and Sonia I. Seneviratne. 2014–10AD. "Global Assessment of Trends in Wetting and Drying over Land." *Nature Geosci* 7 (10). Nature Publishing Group: 716–21. http://dx.doi.org/10.1038/ngeo2247.

Orth, Rene, Randal D. Koster, and Sonia I. Seneviratne. 2013. "Inferring Soil Moisture Memory from Streamflow Observations Using a Simple Water Balance Model." *Journal of Hydrometeorology* 14 (6): 1773–90. doi:10.1175/JHM-D-12-099.1.

Prentice, I. Colin, Ning Dong, Sean M. Gleason, Vincent Maire, and Ian J. Wright. 2014. "Balancing the Costs of Carbon Gain and Water Transport: Testing a New Theoretical Framework for Plant Functional Ecology." *Ecology Letters* 17 (1): 82–91. doi:10.1111/ele.12211.

Shangguan, Wei, Yongjiu Dai, Qingyun Duan, Baoyuan Liu, and Hua Yuan. 2014. "A Global Soil Data Set for Earth System Modeling." *Journal of Advances in Modeling Earth Systems* 6 (1): 249–63. doi:10.1002/2013MS000293.
